# Synthesis and *In Vitro* Biological Evaluation of Quinolinyl Pyrimidines Targeting Type II NADH-Dehydrogenase (NDH-2)

**DOI:** 10.1021/acsinfecdis.1c00413

**Published:** 2022-02-21

**Authors:** Lu Lu, Linda Åkerbladh, Shabbir Ahmad, Vivek Konda, Sha Cao, Anthony Vocat, Louis Maes, Stewart T. Cole, Diarmaid Hughes, Mats Larhed, Peter Brandt, Anders Karlén, Sherry L. Mowbray

**Affiliations:** † Department of Cell and Molecular Biology, BMC, Uppsala University, Box 596, SE-751 24 Uppsala, Sweden; ‡ Department of Medicinal Chemistry, Organic Pharmaceutical Chemistry, BMC, Uppsala University, Box 574, SE-751 23 Uppsala, Sweden; § Department of Medical Biochemistry and Microbiology, BMC, Uppsala University, Box 582, SE-751 23 Uppsala, Sweden; ∥ École Polytechnique Fédérale de Lausanne, EPFL SV/GHI/UPCOL, Global Health Institute, Station no. 19, CH-1015 Lausanne, Switzerland; ⊥ Laboratory of Microbiology, Parasitology and Hygiene (LMPH), University of Antwerp, Universiteitsplein 1, B-2610 Antwerp, Belgium; # Department of Medicinal Chemistry, Science for Life Laboratory, BMC, Uppsala University, Box 574, SE-751 23 Uppsala, Sweden; ∇ Department of Cell and Molecular Biology, Science for Life Laboratory, BMC, Uppsala University, Box 596, SE-751 24 Uppsala, Sweden

**Keywords:** antimicrobials, NDH-2, quinolinyl pyrimidines, tuberculosis, ESKAPE pathogens

## Abstract

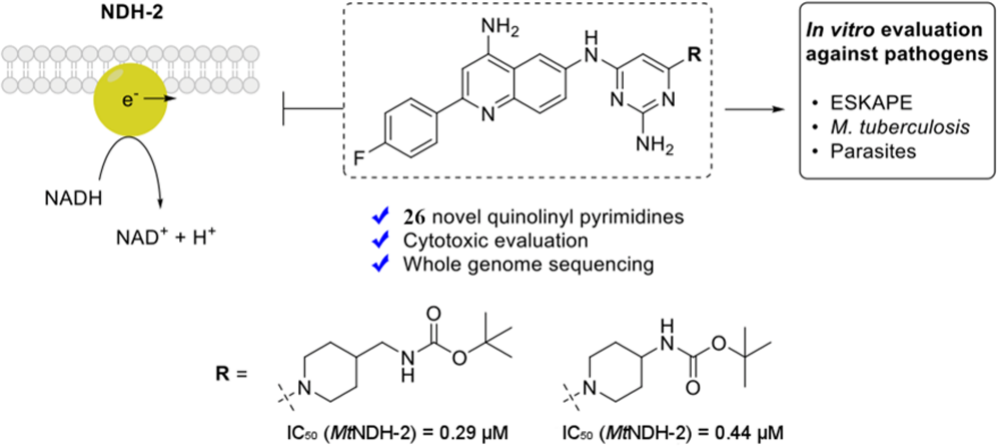

Type II NADH dehydrogenase (NDH-2) is an essential component of electron transfer in many microbial pathogens but has remained largely unexplored as a potential drug target. Previously, quinolinyl pyrimidines were shown to inhibit *Mycobacterium tuberculosis* NDH-2, as well as the growth of the bacteria [ShirudeP. S.; ACS Med. Chem. Lett.2012, 3, 736−7402490054110.1021/ml300134bPMC4025736]. Here, we synthesized a number of novel quinolinyl pyrimidines and investigated their properties. In terms of inhibition of the NDH-2 enzymes from *M. tuberculosis* and *Mycobacterium smegmatis*, the best compounds were of similar potency to previously reported inhibitors of the same class (half-maximal inhibitory concentration (IC_50_) values in the low-μM range). However, a number of the compounds had much better activity against Gram-negative pathogens, with minimum inhibitory concentrations (MICs) as low as 2 μg/mL. Multivariate analyses (partial least-squares (PLS) and principle component analysis (PCA)) showed that overall ligand charge was one of the most important factors in determining antibacterial activity, with patterns that varied depending on the particular bacterial species. In some cases (*e.g.*, mycobacteria), there was a clear correlation between the IC_50_ values and the observed MICs, while in other instances, no such correlation was evident. When tested against a panel of protozoan parasites, the compounds failed to show activity that was not linked to cytotoxicity. Further, a strong correlation between hydrophobicity (estimated as clog *P*) and cytotoxicity was revealed; more hydrophobic analogues were more cytotoxic. By contrast, antibacterial MIC values and cytotoxicity were not well correlated, suggesting that the quinolinyl pyrimidines can be optimized further as antimicrobial agents.

Infectious diseases have an enormous impact on the health and welfare of the global population. Recent trends of increasing resistance to existing antimicrobial drugs are therefore alarming, and new drugs are urgently needed to replace older ones as they lose their potency. At the moment, however, not enough compounds are entering development pipelines to ensure that new treatment options will become available in the next decade. In addition, most drugs currently used share similar mechanisms of action, inhibiting bacterial cell wall biosynthesis or affecting protein synthesis on ribosomes, leaving them vulnerable to further issues of resistance. The recent discovery of bedaquiline and other inhibitors targeting bacterial energy-generating systems opened up new avenues for the development of effective treatment strategies.^1^

In our work, we make extensive use of a same-target-other-pathogen (STOP) strategy, where studies of homologous enzymes from different pathogens give added value to the information gained and compounds synthesized. Where feasible, structural information is obtained by X-ray crystallography and used together with biochemical and medicinal chemistry studies to achieve better inhibition of the target enzymes. A priority is to test for action against relevant pathogens very early on, as this is a major stumbling block in finding new antibiotics. An effective enzyme inhibitor is not necessarily active against any given pathogen (because of problems with uptake, degradation, efflux, *etc.*), so it is essential to broadly profile compounds against multiple pathogens, to find those that are susceptible. As selectivity of action is vital, cytotoxicity and other (physicochemical) properties are also used to help identify potential problems *in vivo*.

The pathogens covered in this work cause a variety of important diseases. Even now, in the midst of the COVID-19 pandemic, they are still huge killers and, in fact, account for a large proportion of deaths nominally attributed to the coronavirus.^2^ Tuberculosis killed about 1.4 million people in 2019.^3^ Approximately one-third of the world’s population is infected with *Mycobacterium tuberculosis*, although most of those infected do not develop the active form of the disease. Co-infection by *M. tuberculosis* and HIV is particularly deadly, and combined with drug resistance, treatment of either infection becomes greatly complicated. Malaria (resulting from infection with one of several *Plasmodium* species) is also an immense problem, especially in the developing world. There are currently about 229 million new cases each year, and 409 000 deaths, mostly among African children (www.who.int). With increasing drug resistance, and climate change, the developed world is expected to become increasingly affected. Additional pathogens have been designated as ESKAPE bacteria (*Enterococcus faecium*, *Staphylococcus aureus* (causing MRSA), *Klebsiella* species, *Acinetobacter baumannii*, *Pseudomonas aeruginosa*, and *Enterobacter* sp.).^4^ These largely Gram-negative organisms cause most nosocomial (hospital-acquired) infections, resulting in many deaths (about 25 000 in Europe alone), much suffering, and massive economic loss (about 1.5 billion euros) each year.^5^ As the ESKAPE bacteria are rapidly becoming resistant to existing drugs, there is an urgent need for new treatment options.

All of these pathogens have an essential type II NADH-dehydrogenase (NDH-2, EC 1.6.5.3), a monotopic membrane protein^6,7^ that takes part in electron transport. A representative electron acceptor is menadione (2-methyl-1,4-naphthoquinone); however, the most common electron carriers are menaquinone derivatives, including ubiquinones, which can also be reduced.^8^ In some pathogens, NDH-2 is the only dehydrogenase in the respiratory chain, while in others, multiple NADH dehydrogenases are known. In *M. tuberculosis*, NDH-2, encoded by the *ndh* gene, is the most favored NADH dehydrogenase of the three enzymes present; it is essential for growth and persistence.^9^ Although the specifics vary with the particular pathogen and its metabolic state, compounds that target NDH-2 are of interest as new options for therapy, perhaps in combination with compounds that block other pathways. The absence of mammalian homologues further adds to NDH-2’s potential interest.

Phenothiazines, including chlorpromazine and triflupromazine, are known inhibitors of the NDH-2 of *M. tuberculosis* (*Mt*NDH-2) with *in vitro* and *in vivo* activity.^10−12^ However, the doses required for antimycobacterial activity are much higher than those eliciting CNS effects,^13^ so they are unsuitable for the treatment of tuberculosis. Furthermore, more recent work suggests that phenothiazines do not inhibit membrane-bound NDH-2 but instead function by disrupting pH gradients across bacterial membranes.^14^ NDH-2 is also known to be sensitive to flavones,^12,15^ but their low potency (half-maximal inhibitory concentrations (IC_50_s) of the order of 750 μM) does not suggest that these are useful starting points for improved compounds. More recently, polymyxin B was identified as an inhibitor of NDH-2,^16^ but this is not its primary mode of action, and such a complex molecule does not offer helpful ideas for the development of small-molecule drugs. A breakthrough was provided when Shirude et al.^17^ discovered that quinolinyl pyrimidines such as **1** (Figure 1) are potent inhibitors of *Mt*NDH-2 (IC_50_s as low as 40 nM) with good *in vitro* antimycobacterial activity (minimum inhibitory concentrations (MICs) as low as 0.8 μM). NDH-2 has also been shown to be targeted by 2-phenylquinolones such as CK-2-68, RYL-552 and RYL-552S,^18^ and MTC420^19^ (see Figure 1).

**Figure 1:**
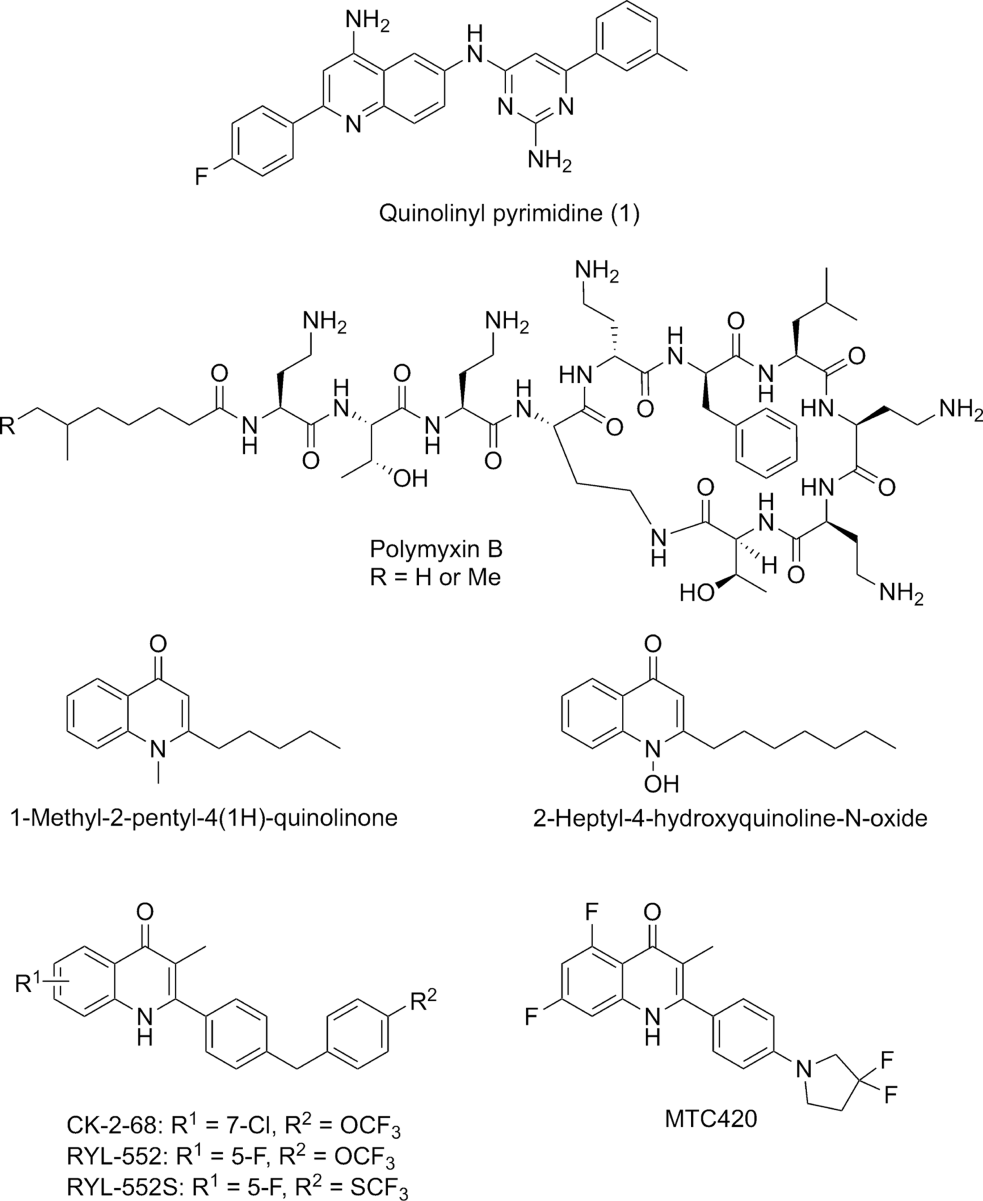
Inhibitors of NDH-2 discussed in the text.

In the present study, we synthesized 26 novel quinolinyl pyrimidines, which were evaluated against mycobacterial NDH-2 enzymes, as well as a number of bacterial and protozoan pathogens. The strategy for choosing compounds to be synthesized can best be described as exploratory. The primary goal was to determine whether optimization would be feasible, and we therefore made a diverse set of compounds to study the effects on various properties. Tests of cytotoxicity and whole genome sequencing of resistant mutants provided additional information to guide future work.

## Results and Discussion

### Chemistry

**Scheme 1:**
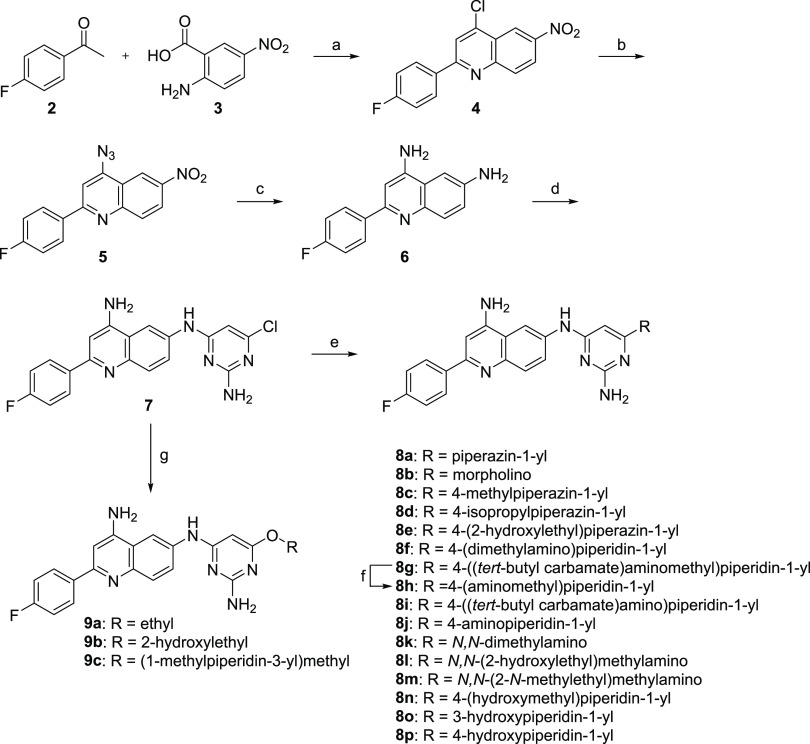
Synthesis of Compounds **8a**–**p** and **9a**–**c** Reagents and conditions: (a) POCl_3_, 90 °C, 5 h, 48%; (b) NaN_3_, *N*-methyl-2-pyrrolidone (NMP), 60 °C, 18 h; (c) SnCl_2_·H_2_O, EtOAc/EtOH (2:1), reflux, 2 h, 75% from **4**; (d) 2-amino-4,6-dichloropyrimidine, HCl (4 M in dioxane), NMP, 100 °C, overnight, 76%; (e) amine, *N*,*N*-diisopropylethylamine (DIEA), absolute EtOH, microwave heating 150 °C, 90 min, 30–69%; (f) mixture of absolute EtOH and 4 M HCl in 1,4-dioxane (1:1), room temperature (rt), 2 h, quantitative yield; (g) alcohol, KOH, microwave heating 150 °C, 30 min or 100–120 °C, 70 min 13–63%.

The quinolone scaffold was synthesized by reacting 4-fluoroacetophenone (**2**) with 2-amino-5-nitrobenzoic acid (**3**) in hot POCl_3_ (Scheme 1).^20^ The resulting 4-chloro-2-(4-fluorophenyl)-6-nitroquinoline (**4**) was then subjected to sodium azide to convert the 4-chloro group into the corresponding azide (**5**). The azide and nitro groups were reduced by tin(II) chloride monohydrate in refluxing ethyl acetate and ethanol to afford 2-(4-fluorophenyl)quinoline-4,6-diamine (**6**). Next, the 6-amino group of compound **6** selectively displaced 2-amino-4,6-dichloropyrimidine to yield key intermediate *N*^6^-(2-amino-6-chloropyrimidin-4-yl)-2-(4-fluorophenyl)quinoline-4,6-diamine (**7**) in 76% yield.

Knowing from previous reports^17^ that the chosen class of compounds was plagued by poor solubility, we replaced the *m*-tolyl group of the reported inhibitor **1** with more hydrophilic groups. Furthermore, we postulated that solubility would be increased by incorporating functionalities that are charged at physiological pH, *e.g.*, amino groups. We saw that amines and alcohols could serve as nucleophiles to substitute the 6-chloro on the pyrimidine ring. To explore the chemical space around the 6-position of the pyrimidine, a diverse set of secondary amines and primary alcohols was selected.

Secondary amines were heated with compound **7** and *N*,*N*-diisopropylethylamine using microwave irradiation^21^ at 150 °C for 90 min, or until all starting material had been consumed, to yield final products **8a**–**l**. Compound **8h** was obtained after Boc-deprotection from compound **8g**. Alkoxy-substituted final compounds were furnished from compound **7** by heating in neat alcohol with potassium hydroxide. This approach afforded compounds **9a**–**c** as the trifluoroacetic acid (TFA) salts in 13–63% yield after semipreparative high-performance liquid chromatography (HPLC) purification.

**Scheme 2:**
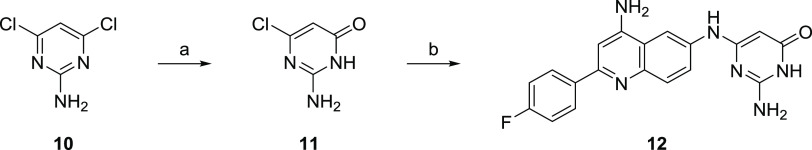
Synthesis of Compound **12** Reagents and conditions: (a) 1 M NaOH, reflux, 2 h, then AcOH, 90%; (b) **6**, HCl (4 M in dioxane), NMP, 100 °C, overnight, 38%.

We also wanted to investigate whether the less-substituted pyrimidone retained activity on NDH-2 enzymes and targeted pathogens. To achieve this, a suspension of 2-amino-4,6-dichloropyrimidine (**10**) was heated at reflux in 1 M sodium hydroxide (aq) to provide 2-amino-6-chloropyrimidin-4(3*H*)-one (**11**) in 90% isolated yield (Scheme 2). Diamine **6** was then heated with compound **11** and hydrochloric acid (4 M in dioxane) in *N*-methylpyrrolidinone at 100 °C overnight to afford 2-amino-6-((4-amino-2-(4-fluorophenyl)quinolin-6-yl)amino)pyrimidin-4(3*H*)-one (**12**) in 38% yield.

**Scheme 3:**
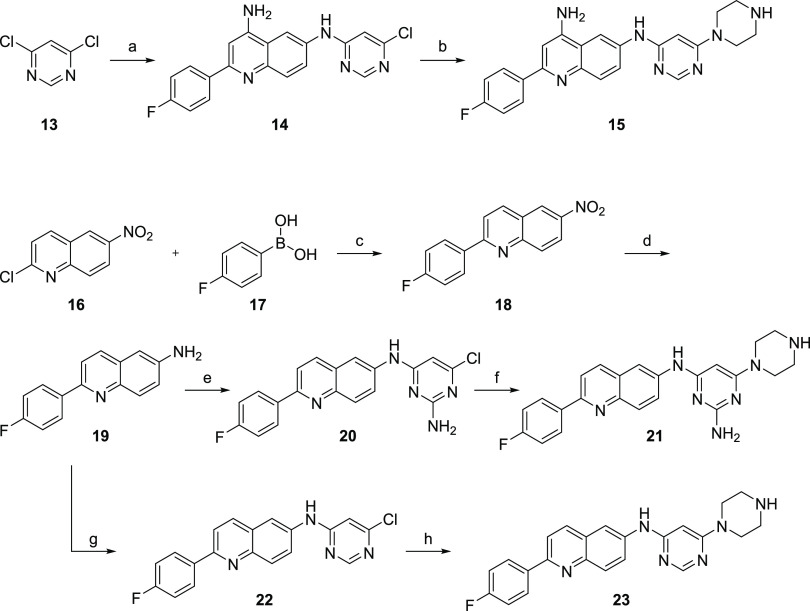
Synthesis of Compounds **15**, **21**, and **23** Reagents and conditions: (a) **6**, HCl (4 M in dioxane), NMP, 100 °C, 6 h, 30%; (b) piperazine, DIEA, absolute EtOH, microwave heating 150 °C, 90 min, 59%; (c) Pd(PPh_3_)_4_, K_2_CO_3_, dimethoxyethane/H_2_O (8:1), microwave heating 150 °C, 30 min, 85%; (d) H_2_, Pd/C, MeOH/EtOAc (1:1), rt, 8 h; (e) 2-amino-4,6-dichloropyrimidine, HCl (4 M in dioxane), NMP, 100 °C, overnight, 79% from **18**; (f) piperazine, DIEA, absolute EtOH, microwave heating 150 °C, 2.5 h, 42%; (g) 4,6-dichloropyrimidine (**13**), HCl (4 M in dioxane), NMP, 110 °C, overnight; (h) piperazine, DIEA, absolute EtOH, microwave heating 150 °C, 90 min, 51%.

To further explore the structure–activity relationship (SAR) of the 4-quinoline and 2-pyrimidine amines, analogues of **8a** were synthesized without one or both of the exocyclic amines (compounds **15**, **21**, and **23**; Scheme 3). Compound **6** was reacted with 4,6-dichloropyrimidine (**13**) to afford *N*^6^-(6-chloropyrimidin-4-yl)-2-(4-fluorophenyl)quinoline-4,6-diamine (**14**) in 30% isolated yield. Displacement of the pyrimidine chloride with piperazine furnished final compound 2-(4-fluorophenyl)-*N*^6^-(6-(piperazin-1-yl)pyrimidin-4-yl)quinoline-4,6-diamine (**15**) in 59% yield.

To prepare compounds lacking the 4-quinoline amine group, a different quinoline intermediate was prepared. A microwave-heated Suzuki coupling reaction^22^ with 2-chloro-6-nitroquinoline (**16**) and 4-fluorophenylboronic acid (**17**) yielded 2-(4-fluorophenyl)-6-nitroquinoline (**18**). After reduction of the nitro group and subsequent substitution of the 6-amino group (**19**), 6-chloro-*N*^4^-(2-(4-fluorophenyl)quinolin-6-yl)pyrimidine-2,4-diamine (**20**) was obtained. Substitution with piperazine produced the final compound (**21**). To prepare the analogue lacking both amines, compound **19** was reacted with pyrimidine **13** to yield *N*-(6-chloropyrimidin-4-yl)-2-(4-fluorophenyl)quinolin-6-amine (**22**). Final compound 2-(4-fluorophenyl)-*N*-(6-(piperazin-1-yl)pyrimidin-4-yl)quinolin-6-amine (**23**) was obtained after subsequent substitution with piperazine.

### Biology and SAR

#### Inhibition of Mycobacterial NDH-2s

Inhibition of two mycobacterial enzymes by quinolinyl pyrimidines is reported in Table 1. One represents *Mt*NDH-2 expressed in *Mycobacterium smegmatis*, while the other is *M. smegmatis* NDH-2 (*Ms*NDH-2) expressed in *Escherichia coli*. Both enzymes contain a C-terminal His-tag. The *Ms*NDH-2 IC_50_s give much the same picture as those for *Mt*NDH-2, despite being on average about 4-fold higher, suggesting that this is a reasonable alternative for ranking compounds when expression in *M. smegmatis* is not a good option. The two sequences are, however, only ∼80% identical, which could account for some of the differences.

**Table 1:**
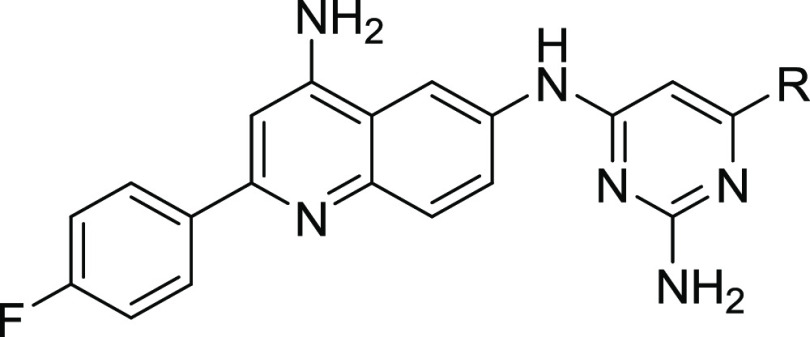

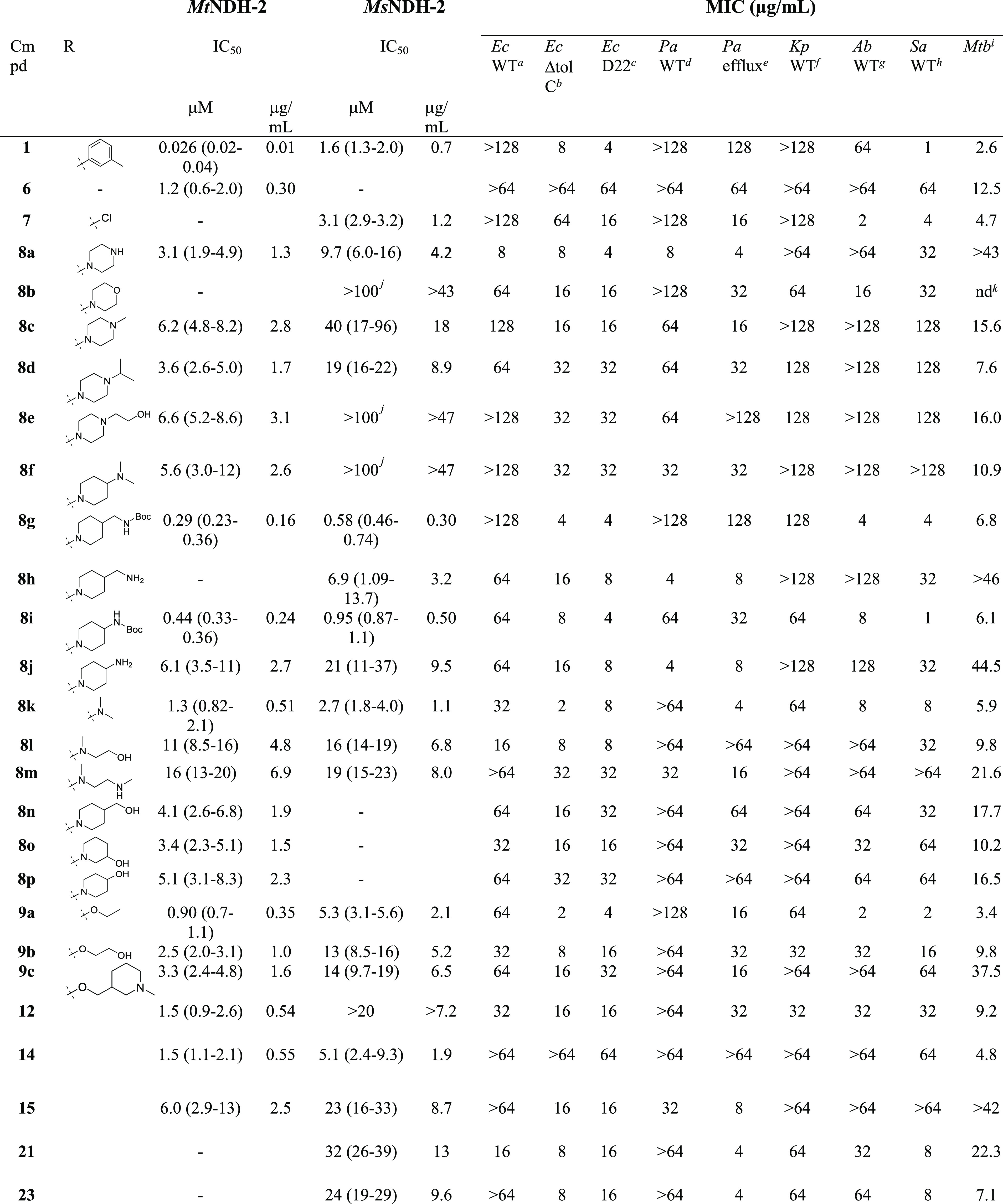
Inhibition of *Mt*NDH-2 and *Ms*NDH-2, and Measured Minimum Inhibitory Concentration (MIC) Values on a Panel of Gram-Negative Bacteria, the Gram-Positive *S. aureus*, and *M. tuberculosis* (*Mtb*)^l^

a *E. coli* (ATCC 25922, wild-type).

b *E. coli* (ΔtolC efflux-defective mutant, isogenic to ATCC 25922).

c *E. coli* (D22, *lpxC* mutant, drug-hypersensitive).

d *P. aeruginosa* (PAO1, wild-type).

e *P. aeruginosa* (PAO750 efflux-defective mutant, isogenic to PAO1).

f *K. pneumonia* (ATCC 13833, wild-type).

g *A. baumannii* (ATCC 19606, wild-type).

h *S. aureus* (ATCC 29213, wild-type).

i *M. tuberculosis* strain H37Rv (ATCC 25618).

j No significant inhibition was observed at 100 μM.

k nd = not determined.

l IC_50_ values are given in μM, along with the 95% confidence intervals in parentheses, followed by values in μg/mL to assist comparisons with MICs.

Compound **1** had been reported earlier to inhibit *Mt*NDH-2 with an IC_50_ of 96 nM;^17^ under our standardized assay conditions, the IC_50_ was 26 nM. When the *m*-tolyl group of **1** was replaced with the more hydrophilic piperazine, morpholine, and 4-methyl-piperazine (**8a**–**c**), inhibition was significantly poorer. Substitution of piperazine and piperidine with some bulkier groups (**8d**–**f**) also appeared to be unfavorable, although bulky Boc-protected **8g** and **8i** were relatively good inhibitors. The corresponding deprotected amines (**8h** and **8j**) had IC_50_s approximately 10-fold higher than their Boc-protected counterparts, suggesting that hydrophobic interactions may be required in that part of the structure or that hydrophilic interactions are not desirable. The introduction of a small hydrophilic chain in **8l** and **8m** gave IC_50_s of ∼10 μM for both enzymes. The additional changes in **8n**–**p** gave only modest improvement of inhibition. When a small alkoxy group was instead introduced on the pyrimidine ring (**9a**), a lower IC_50_ was observed. When the ethoxy group in **9a** was replaced with 2-hydroxylethoxy (**9b**) or (1-methylpiperidine-3-yl)methoxy (**9c**), inhibition was roughly 3-fold weaker. Exchanging the pyrimidine ring for a pyrimidone (**12**) did not change potency significantly, which shows that changes in this part of the structure are possible. Removal of the 2-pyrimidine amine (**15**) gave a similar IC_50_ value to that of the corresponding compound **8a**, suggesting that this functionality is not essential for activity on the enzyme. Additionally, removal of the 4-quinoline amine (**21**), or both the 4-quinoline and 2-pyrimidine amines (**23**), resulted in activity on the *Ms*NDH-2 enzyme equivalent to that of the corresponding compound **8a (***Mt*NDH-2 was not tested in this case).

A number of X-ray structures have been solved for NDH-2-like enzymes in complex with different kinds of inhibitors (summarized in Petri et al.^23^); none involves a quinolinyl pyrimidine. The most similar protein is the enzyme from *Caldalkalibacillus thermarum*,^23^ with 31% amino acid sequence identity to *Mt*NDH-2, bound to 2-heptyl-4-hydroxyquinoline-*N*-oxide (Figure 1). Docking of the present compounds based on this structure is problematic because the inhibitors are not very similar, and the sequence identity is not very high, particularly in the C-terminal regions relevant for binding. However, if the mode of docking the quinolinyl pyrimidines into *Mt*NDH-2 proposed by Petri et al. is correct, a C-terminal hydrophobic groove would be interacting with the phenyl pyrimidine group of our present compounds. The essential primary amine on the pyrimidine^17^ would then be interacting with some *Mt*NDH-2 group within this groove. The 4-fluorophenyl group would be placed in a hydrophobic pocket near the fused-ring system of the flavin adenine dinucleotide (FAD); the essential primary amino group of the quinoline^17^ could, for instance, form a hydrogen bond with the carbonyl group at C4 of the flavin. Clearly, more X-ray structures will be required to understand inhibitor binding in the various enzymes.

#### MIC Evaluation on ESKAPE Bacteria and *M. tuberculosis*

The new compounds were evaluated for antibacterial activity against a panel of ESKAPE bacteria, as well as *M. tuberculosis* (using a resazurin reduction microplate assay (REMA)^24^), see Table 1. The quinolinyl pyrimidine inhibitor **1**, reported earlier as an inhibitor of *M. tuberculosis* growth, was essentially inactive on Gram-negative bacteria with the exception of the efflux-defective (Δ*tolC*) and drug-hypersensitive (*lpxC*) *E. coli* strains. However, the MIC for this compound on the mycobacterium was found to be in line with the value previously reported.^17^

**Figure 2:**
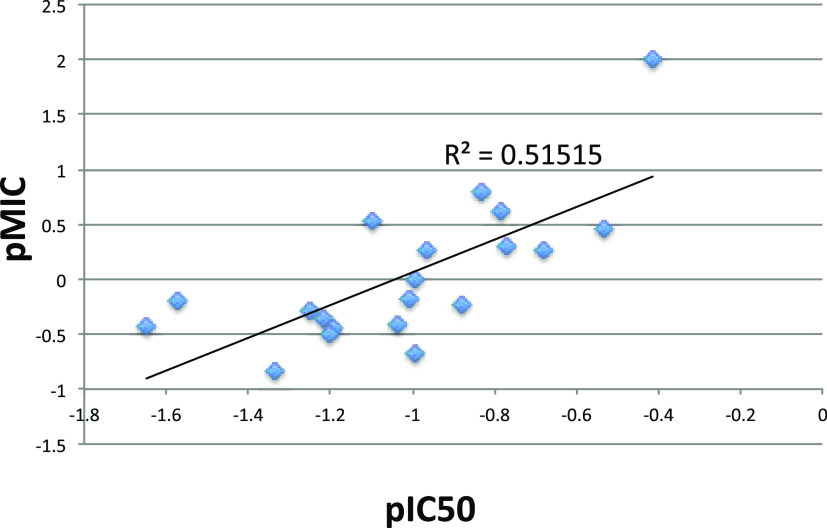
Relationship between inhibition of *Mt*NDH-2 (expressed as pIC_50_) and whole-cell activity against *M. tuberculosis* (expressed as pMIC), both in terms of μg/mL.

Although the IC_50_s for *Mt*NDH-2 generally correlate well with MICs on *M. tuberculosis* (see Figure 2), a few exceptions were noted (**8a**, **8j**, **9c**, and **15**). Compounds **8a**, **8d**, and **9c** have very similar IC_50_s (approx. 1.5 μg/mL), but widely varying MICs. The same is true for **8c**, **8j**, and **15**. Note that **15** is an analogue of **8a**, as indeed are **21** and **23**. In terms of MIC, removing both exocyclic amines as in **23** was preferable; in terms of IC_50_, the reverse was the case. The differences in MIC could arise from variations in the ability to penetrate the notably thick and impermeable mycobacterial cell wall.

**Figure 3:**
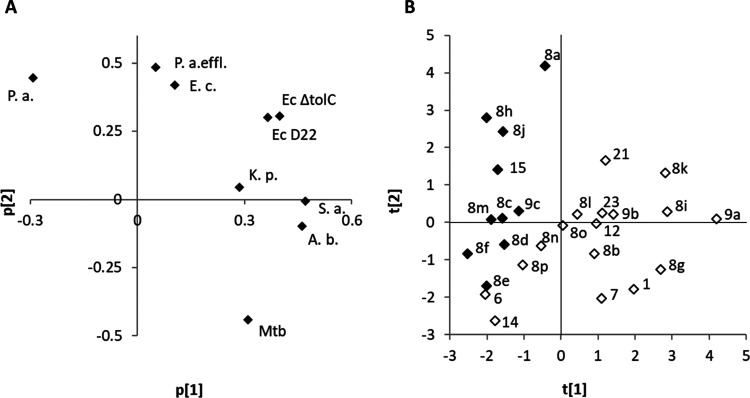
Principal component analysis showing (A) the correlation structure in the MIC values and (B) the scores for all tested inhibitors, highlighting how the number of positive charges influences the antibacterial profile. Filled diamonds have two positive charges, and open diamonds have one positive charge.

To gain a better understanding of how structural changes modulate the MIC values in general, and the relative MIC values for various strains, a partial least-squares (PLS) analysis was performed including also a few standard molecular descriptors (see the Methods for details). This analysis revealed that the overall ligand charge is one of the most important factors affecting the MICs. Figure 3 shows a principal component analysis based on MIC data only, highlighting how the overall charge affects the antibacterial patterns. Thus, good MICs on wild-type *P. aeruginosa* are dependent on the presence of a charged group on the pyrimidine (*e.g.*, **8a**, **8h**,and **8j**) whereas MICs on *A. baumannii* and *M. tuberculosis* are improved by neutral groups. Moreover, replacing the *m*-tolyl group of **1** with both smaller (−Cl and −OEt, **7** and **9a**, respectively) and larger (Boc-protected amines, *e.g.*, **8g** and **8i**) noncharged substituents was linked to retained activity against *M. tuberculosis* and *A. baumannii* (see Table 1).

Interestingly, compounds containing a primary (**8h** and **8j**) or secondary amine (**8a**) inhibited both *P. aeruginosa* wild-type and mutated strains at a similar level. By contrast, when a less basic tertiary amine (*e.g.*, **8c**–**e**) or a neutral substituent (*e.g*., **8b** and **9b**) is introduced, the *P. aeruginosa* wild-type activity is lost (MIC ≥ 64 μg/mL). This suggests that a positive charge in this part of the structure is essential for activity on wild-type *P. aeruginosa*, although the role of this amine is as yet unclear.

For some of the strains tested (*E. coli* D22, *A. baumannii*, *S. aureus*, and *M. tuberculosis*) correlations between pIC_50_ and pMIC values (*r* between 0.48 and 0.69) were observed. For the bacteria least susceptible to the tested class of compounds (*E. coli*, *P. aeruginosa*, and *Klebsiella pneumoniae* wild types), such correlations were not seen (*r* between −0.30 and 0.03). The tested compounds generally show little inhibition of wild-type *E. coli* growth, although several did inhibit the Δ*tolC* and *lpxC* strains. Together, these observations suggest that this class of compounds needs optimization in terms of efflux and permeability.

#### Evaluation of Cytotoxicity

Cytotoxicity was assessed against MRC-5_SV2_ (human lung fibroblast) cells as well as a human hepatoblastoma cell line, HepG2 (Table 2). Where both cell lines were tested, the trends in toxicity correlate well; although MRC-5 IC_50_s were always higher than HepG2 TD_50_s, compounds that were toxic to HepG2 cells were also toxic to MCR-5 cells. Compound **9a** stands alone as an example where toxicity against MRC-5 cells is greater. In general, the cytotoxicity of the various compounds was striking, and furthermore, inhibitors were relatively cytotoxic at concentrations similar to the corresponding IC_50_ values. However, there was no clear correlation between high cytotoxicity and the IC_50_ values, suggesting that these two properties could be optimized independently.

**Table 2:** Calculated log *P* and Results from Cytotoxicity Assays on Human Hepatoblastoma (HepG2) and Human Lung Fibroblast Cells (MRC-5)^c^

		cytotoxicity		
		HepG2^a^		MRC-5
compound	clog *P*^b^	TD_99_	TD_50_	IC_50_
**1**	6.0	0.4 (0.2)	0.2 (0.1)	1.3 (0.6)
**6**	2.7	50 (13)	17 (4.3)	nd^d^
**7**	4.3	0.8 (0.3)	0.4 (0.2)	1.1 (0.4)
**8a**	3.6	nd	nd	24 (10)
**8b**	3.9	nd	nd	nd
**8c**	4.0	3.1 (1.4)	1.5 (0.7)	nd
**8d**	4.8	1.6 (0.8)	0.9 (0.4)	nd
**8e**	3.3	13 (6.2)	4.4 (2.1)	nd
**8f**	4.2	6.3 (3.0)	2.8 (1.3)	nd
**8g**	5.3	0.8 (0.4)	0.4 (0.2)	1.4 (0.8)
**8h**	3.8	nd	nd	8.0 (3.7)
**8i**	4.8	0.4 (0.2)	0.2 (0.1)	1.3 (0.7)
**8j**	3.4	25 (11)	8.8 (3.9)	22.0 (9.8)
**8k**	4.1	1.6 (0.6)	0.9 (0.4)	1.8 (0.7)
**8l**	3.5	6.3 (2.6)	3.3 (1.4)	7.8 (3.3)
**8m**	3.8	3.1 (1.3)	1.8 (0.8)	7.6 (3.3)
**8n**	3.9	6.3 (2.6)	2.5 (1.1)	3.7 (1.7)
**8o**	3.9	6.3 (2.8)	1.5 (0.7)	nd
**8p**	3.5	6.3 (2.8)	3.4 (1.5)	nd
**9a**	4.2	0.8 (0.3)	0.4 (1.6)	1.6 (0.6)
**9b**	3.2	6.3 (2.6)	3.1 (1.3)	8.0 (3.3)
**9c**	4.4	1.6 (0.8)	0.9 (0.4)	nd
**12**	2.3	50 (18)	29 (11)	nd
**14**	4.4	1.6 (0.6)	0.7 (0.3)	nd
**15**	3.8	nd	nd	1.7 (0.7)
**21**	4.4	6.3 (2.6)	2 (0.8)	8.0 (3.3)
**23**	4.6	6.3 (2.5)	2.3 (0.9)	7.9 (3.2)

a Rifampicin (TD_99_ ≥ 100 μM; TD_50_ = 75 μM) and bedaquiline (TD_99_ ≥ 100 μM; TD_50_ = 50 μM) were used as reference compounds.

b clog *P* values were calculated by Instant JChem (version 15.9.14.0, ChemAxon).

c Values are given in μM, with values in μg/mL in parentheses to assist comparisons.

d nd = not determined.

**Figure 4:**
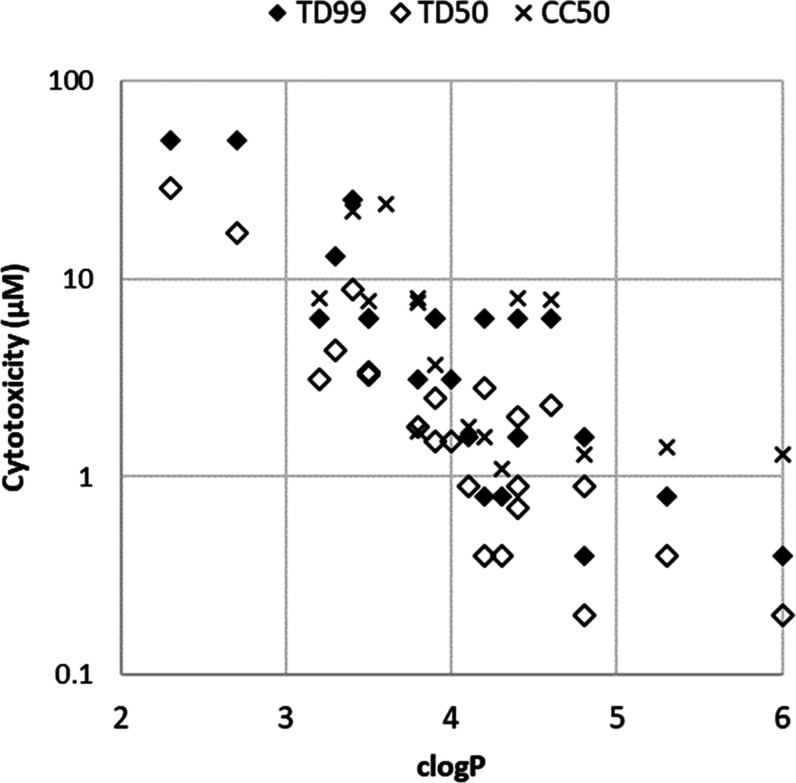
Relationship between calculated log *P* (clog *P*) and cytotoxicity: TD_99_ HepG2 (filled diamonds), TD_50_ HepG2 (open diamonds), CC_50_ MRC-5 (crosses).

Conversely, the SAR reveals that there is a strong positive correlation between hydrophobicity (estimated as clog *P*) and cytotoxicity (Figure 4). This trend is particularly clear for the more hydrophobic Boc-protected **8g** and **8i**, which have considerably lower CC_50_ on MRC-5 cells than the deprotected amines **8h** and **8j**. Furthermore, comparing **9a** with **9b** (ethyl *vs* hydroxyethyl), the 1 log reduction of clog *P* results in more than 5-fold reduction of cytotoxicity. Similarly, the compounds with the lowest clog *P*s, **6** and **12**, have the lowest toxicity. The less-substituted pyrimidone **12** stands out as an acceptable inhibitor of *Mt*NDH-2 with reasonable activity against *M. tuberculosis*, although its effects against the ESKAPE pathogens are more modest.

We also sought to determine whether the anilinic amines of the quinoline and pyrimidine scaffolds were giving rise to cytotoxicity. By comparing the substitution patterns of **8a**, **15**, **21**, and **23**, and considering the clog *P* values, it can be concluded that the pyrimidine and quinoline NH_2_ groups are not the key drivers for cytotoxicity. By means of PLS, a model for the cytotoxicity was generated, indicating a positive correlation between cytotoxicity and molecular weight (MW), and a negative correlation to descriptors contributing to the decrease of the hydrophobicity (see Figure S1).

#### Evaluation on Parasites

Comparison of the cytotoxicity results and the effects on *in vitro* growth of the multidrug-resistant *P. falciparum* K1 strain, *Trypanosoma brucei brucei* Squib 427, *Trypanosoma cruzi* Tulahuen LacZ (clone C4), and *Leishmania infantum* MHOM/MA(BE)/67 strains (Table S1) suggested only nonspecific parasite growth inhibition (maximum concentration tested was 64 μM).

#### Selection and Sequence Identification of Resistant Mutants of ESKAPE Pathogens

Resistant mutants were raised against **8a**, **8g**, **8j**, and **9a**. The results obtained when these were analyzed by whole-genome sequencing are summarized in Table 3. The best of the new NDH-2 inhibitors, **8g**, does not give rise to mutations in the *ndh* gene that codes for NDH-2, instead producing primarily mutations in *ackA* (coding for acetate kinase). The links between this enzyme and NDH-2 are not clear. The poorer inhibitors **8a** and **8j** give rise to mutations in members of a two-component signaling system, *pmrA* (basR) and *pmrB* (*basS*), as was reported earlier for mutations that cause resistance to polymyxin B, a known NDH-2 inhibitor that also has several other biological effects.^25^ The moderate inhibitor **9a** gave rise to yet another pattern of mutations. It is known that polymyxin B has a number of different effects on Gram-negative cells, including disrupting the outer membrane. More work will be required to determine whether quinolinyl pyrimidines have other effects, in addition to inhibiting NDH-2.

**Table 3:** Whole Genome Sequencing of Resistant Mutants Raised against **8a**, **8g**, **8j**, and **9a**^a^

strain	parent strain	compound	MIC mg/L mutant	fold increase	mutation(s)
CH4954	*E. coli* MG1655 Δ*tolC*	**8a**	8	2	*basS* (*pmrB*) Glu121Lys *waaY* (*rfaY*) Asp160fs
CH4955	*E. coli* MG1655 Δ*tolC*	**8a**	16	4	*basR* (*pmrA*) Gly53Glu
CH4956	*E. coli* MG1655 Δ*tolC*	**8a**	8	2	*basS* (*pmrB*) Leu10Arg
CH4957	*E. coli* MG1655 Δ*tolC*	**8a**	16	4	*asmA* Asn295fs *waaP* (*rfaP*) Glu258^b^
CH5084	*E. coli* MG1655 Δ*tolC*	**8a**	16	4	*basS* (*pmrB*) Ala159Pro *preB* Ala13fs
CH5053	*E. coli* MG1655 Δ*tolC*	**8g**	16	4	*ackA* Leu255Arg
CH5054	*E. coli* MG1655 Δ*tolC*	**8g**	16	4	*ackA* Glu375^b^
CH5055	*E. coli* MG1655 Δ*tolC*	**8g**	16	4	*ackA* Thr233Pro
CH5056	*E. coli* MG1655 Δ*tolC*	**8g**	16	4	*ackA* Gly275Asp
CH5057	*E. coli* MG1655 Δ*tolC*	**8g**	8	2	*eno* Pro38Leu
CH5058	*E. coli* MG1655 Δ*tolC*	**8g**	16	4	*ackA* Ile92fs
CH5059	*E. coli* MG1655 Δ*tolC*	**8g**	16	4	*ackA* Arg91Ser
CH5062	*E. coli* MG1655 Δ*tolC*	**8g**	16	4	*ackA* Gly241Asp
CH5063	*E. coli* MG1655 Δ*tolC*	**8g**	16	4	*ackA* Arg91Leu
CH5071	*P. aeruginosa* PAO1	**8j**	32	8	*basS* (*pmrB*) Leu17Gln
CH5072	*P. aeruginosa* PAO1	**8j**	32	8	*phoQ* Leu158fs
CH5073	*P. aeruginosa* PAO1	**8j**	32	8	*basS* (*pmrB*) Ser257Asn
CH5075	*P. aeruginosa* PAO1	**8j**	32	8	*basS* (*pmrB*) Ala29Glu
CH5077	*P. aeruginosa* PAO1	**8j**	32	8	*phoQ* Ser151^b^
CH5080	*A. baumannii* ATCC19606	**9a**	4	2	*coaE* Gly10Asp *omp38* (upstream)

a fs, frameshift.

b Stop codon.

## Conclusions

Twenty-six novel quinolinyl pyrimidines were designed, synthesized, and evaluated as NDH-2 inhibitors, as well as for their effects on bacteria and parasitic protozoa, and cytotoxicity. Most of the compounds were cytotoxic. Those that were not were also relatively poor inhibitors of NDH-2. However, it is worth stressing that cytotoxicity and inhibition are not correlated, and so the properties can be optimized separately. We conclude that, although the enzyme can probably be targeted successfully, this particular series of compounds may not offer the best prospects for systemic drug treatment. However, similar problems for polymyxin B have meant that its primary approved use in Europe is as a highly effective topical drug, and this avenue could be explored for the quinolinyl pyrimidines, as well.

## Methods

### General Information

Reagents and solvents were obtained from Sigma-Aldrich (St. Louis, MO) and Fischer (Pittsburgh, PA) and used without further purification. Middlebrook 7H9 was provided by Difco, albumin dextrose catalase (ADC) was provided by Chemie Brunschwig AG (Switzerland), and Dulbecco’s modified Eagle’s medium (DMEM), trypsin–ethylenediaminetetraacetic acid (EDTA) 0.05%, phosphate-buffered saline (PBS) (1×) pH 7.4, and fetal bovine serum (FBS) (heat-inactivated) were provided by Gibco (Switzerland). Thin-layer chromatography (TLC) was performed on aluminum sheets precoated with silica gel 60 F254 (0.2 mm, Merck KGaA, Darmstadt, Germany). Column chromatography was performed using silica gel 60 (40–63 lm, Merck KGaA, Darmstadt, Germany). Microwave reactions were carried out in a Smith Synthesizer or in an Initiator single-mode microwave cavity producing controlled irradiation at 2450 MHz. Analytical reversed-phase HPLC-mass spectrometry (MS) was performed on a Dionex Ultimate 3000 system using MeCN/H_2_O (0.05% HCOOH) as the mobile phase with MS detection (ion trap), equipped with a C18 (Phenomenex Kinetex SBC18 (4.8 × 50 mm^2^)) column using UV (214 or 254 nm) detection or on a Dionex Ultimate 3000 system using MeCN/H_2_O (0.05% HCOOH) as the mobile phase with MS detection (electrospray ionization (ESI)), equipped with a C18 (Phenomenex Kinetex SB-C18 (4.8 × 50 mm^2^)) column using a UV diode array detector. Semipreparative reversed-phase HPLC was performed on a Gilson-Finnigan ThermoQuest AQA system equipped with a C8 (Zorbax SB-C8 (5 μm, 150 × 21.2 mm^2^)) column using MeCN/H_2_O (0.1% TFA) as the mobile phase with UV or on a Gilson GX-271 system equipped with a C18 (Macherey-Nagel Nucleodur HTec (5 μm, 125 × 21 mm^2^)). Purity analyses were run using a gradient of 5–100% MeCN/H_2_O (0.05% HCOOH) at a flow rate of 1.5 mL/min for 5 min on a C18 (Phenomenex Kinetex SBC18 (4.8 × 50 mm^2^)) column unless otherwise stated. NMR spectra were recorded on a Varian Mercury plus spectrometer (^1^H at 399.8 MHz, ^13^C at 100.5 MHz) at ambient temperature. Chemical shifts (δ) are referenced to tetramethylsilane (TMS) *via* residual solvent signals (^1^H: CDCl_3_ δ 7.26 ppm, MeOD δ 3.31 ppm and dimethyl sulfoxide (DMSO)-*d*_6_ δ 2.50 ppm; ^13^C: CDCl_3_ δ 77.0 ppm, MeOD δ 49.0 ppm and DMSO-*d*_6_ δ 39.5 ppm). High-resolution masses (HRMS) were determined on a mass spectrometer equipped with an ESI source and time-of-flight (TOF) mass analyzer.

### Synthetic Procedures and Characterization of Compounds **1**, **4**–**23**

#### *N*^6^-(2-Amino-6-*m*-tolylpyrimidin-4-yl)-2-(4-fluorophenyl)quinoline-4,6-diamine (**1**)^17^

A mixture of 2-amino-6-*m*-tolylpyrimidin-4-ol (70 mg, 0.36 mmol), triethylamine (0.05 mL, 0.36 mmol), and *N*-phenyl-bis(trifluromethanesulfonamide) (130 mg, 0.36 mmol) in *N*-methyl-2-pyrrolidinone (1.5 mL) was heated at 60 °C for 2 h under nitrogen. 2-(4-Fluorophenyl) quinoline-4,6-diamine (**6**) (90 mg, 0.36 mmol) was dissolved in *N*-methyl-2-pyrrolidinone (1 mL) and added to the above reaction followed by the addition of HCl (4 M in dioxane, 0.36 mL, 1.42 mmol). The mixture was heated at 80 °C for 10 h where upon a solid was formed. The reaction mixture was allowed to cool, and then methanol (10 mL) was added. The resulting solid was collected by filtration and washed with methanol. The crude was purified by preparative HPLC (MeCN/H_2_O (0.1% TFA)). All pure fractions were pooled, pH was set to basic, and the product was extracted with ethyl acetate. The combined organic layers were dried over Na_2_SO_4_, filtered, and concentrated to yield a yellow solid (60 mg, 39%). ^1^H NMR (MeOD) δ 8.89 (s, 1H), 8.14 (dd, *J* = 9.2, 2.3 Hz, 2H), 8.03 (d, *J* = 9.2 Hz, 1H), 7.99–7.90 (m, 2H), 7.66–7.58 (m, 3H), 7.55–7.48 (m, 2H), 7.46–7.37 (m, 2H), 7.05 (s, 1H), 6.67 (s, 1H), 2.48 (s, 3H). ^13^C NMR (DMSO-*d*_6_) δ 164.1 (d, ^1^*J*_CF_ = 250.3 Hz), 161.8, 157.4, 153.6, 149.7, 138.75, 136.8, 135.9, 132.5, 131.3, 130.7 (d, ^3^*J*_CF_ = 9.1 Hz), 129.3, 128.8 (d, ^4^*J*_CF_ = 3.0 Hz), 128.7, 127.3, 124.0, 121.4, 116.5 (d, ^2^*J*_CF_ = 22.2 Hz), 115.7, 112.2, 100.9, 95.7, 20.9. One carbon missing.

#### 4-Chloro-2-(4-fluorophenyl)-6-nitroquinoline (**4**)

2-Amino-5-nitro-benzoic acid (1.00 g, 5.49 mmol) was suspended in phosphorous oxychloride (3.0 mL, 33 mol) at room temperature. 4-Fluoroacetophenone (0.670 mL, 5.49 mmol) was added dropwise, and the resulting mixture was heated at 90 °C for 5 h. After completion of the reaction, most of the POCl_3_ was evaporated under reduced pressure and the resulting residue was then poured into a mixture of ice, 25% ammonium hydroxide, and chloroform (10:4:4 w/v). The reaction mass was stirred at room temperature overnight. The chloroform layer was then separated and washed with brine, dried over Na_2_SO_4_, filtered, and evaporated under vacuum to give the crude product as a yellow powder. The crude product was purified by eluting with 5% EtOAc in hexanes (250 mL) and finally with 100% methylene dichloride to give 4-chloro-2-(4-fluorophenyl)-6-nitroquinoline (**4**) as a yellow solid (800 mg, 48%). ^1^H NMR (DMSO-*d*_6_) δ 8.90 (d, *J* = 2.6 Hz, 1H), 8.59 (s, 1H), 8.52 (dd, *J* = 9.2, 2.6 Hz, 1H), 8.45–8.38 (m, 2H), 8.27 (d, *J* = 9.2 Hz, 1H), 7.45–7.37 (m, 2H). ^13^C NMR (DMSO-*d*_6_) δ 164.1 (d, ^1^*J*_CF_ = 249.6 Hz), 158.6, 150.1, 145.5, 144.1, 133.1 (d, ^4^*J*_CF_ = 2.9 Hz), 131.7, 130.3 (d, ^3^*J*_CF_ = 8.8 Hz), 124.4, 123.7, 120.4, 120.3, 116.0 (d, ^2^*J*_CF_ = 21.6 Hz). HRMS (ESI-TOF) calcd for C_15_H_9_N_2_O_2_ClF [M + H]^+^ 303.0337 *m*/*z*, found 303.0345. LC purity (254 nm) >98%.

#### 2-(4-Fluorophenyl)quinoline-4,6-diamine (**6**)

Sodium azide (2.15 g, 33.0 mmol) was added in one step to a solution of 4-chloro-2-(4-fluorophenyl)-6-nitroquinoline (**4**) (1.00 g, 3.30 mmol) in *N*-methylpyrrolidone (10 mL) in a round-bottom flask fitted with a condenser. The resulting suspension mixture was stirred and warmed to 60 °C for 18 h. At the end of this time, the mixture was cooled to room temperature and poured into a mixture of cold water and 50 mL of ethyl acetate. pH was adjusted to 9 with ammonia (aq), and the layers were separated. The aqueous layer was extracted with ethyl acetate (2 × 50 mL) and the combined organic layers were washed with brine and dried over MgSO_4_ and concentrated under reduced pressure to give dark semiviscous crude product (solidified over 2 h at room temp). The crude product was used in the next step without further purification. Crude 6-nitro-4-azido-2-(3-fluorophenyl)-quinoline (**5**) (1.00 g, 3.23 mmol) obtained from the previous step was dissolved in ethyl acetate (50 mL) and ethanol (25 mL). The stirred mixture was heated to reflux, and SnCl_2_·H_2_O (4.38 g, 19.4 mmol) was cautiously added in portions over 10 min. The reaction mixture was heated for an additional 2 h and then cooled to room temperature. Water (100 mL) was added to the reaction mixture, and pH was adjusted cautiously to 9, after which the solution was filtered. Solids were washed with a minimum amount of ethyl acetate. The filtrates were combined, and the aqueous layer was extracted with ethyl acetate. The combined organic layers were washed with brine, dried over MgSO_4_, and filtered before evaporation to give the crude product as a black thick mass. The crude product was chromatographed on a silica gel column, using 10% methanol in ethyl acetate as an eluent. Column purified compound was triturated with petroleum ether to give 4,6-diaminoquinoline (**6**) as a dark tan powder (826 mg, 75%). ^1^H NMR (DMSO-*d*_6_) δ 8.10–7.97 (m, 2H), 7.58 (d, *J* = 8.9 Hz, 1H), 7.33–7.20 (m, 2H), 7.06 (dd, *J* = 8.9, 2.5 Hz, 1H), 6.98–6.92 (m, 2H), 6.25 (br s, NH_2_, exchanges with deuterium), 5.25 (br s, NH_2_, exchanges with deuterium). ^13^C NMR (DMSO-*d*_6_) δ 162.3 (d, ^1^*J*_CF_ = 244.6 Hz), 150.7, 150.2, 145.2, 142.1, 136.8 (d, ^4^*J*_CF_ = 2.9 Hz), 129.9, 128.2 (d, ^3^*J*_CF_ = 8.3 Hz), 121.2, 119.3, 115.2 (d, ^2^*J*_CF_ = 21.4 Hz), 100.7, 98.8. HRMS (ESI-TOF) calcd for C_15_H_13_N_3_F [M + H]^+^ 254.1094 *m*/*z*, found 254.1102. LC purity (254 nm) = 96%.

#### *N*^6^-(2-Amino-6-chloropyrimidin-4-yl)-2-(4-fluorophenyl)quinoline-4,6-diamine (**7**)

Compound **6** (0.62 g, 2.45 mmol) was suspended in *N*-methylpyrrolidone (8 mL) and 2-amino-4,6-dichloropyrimidine was added to the above dark solution in one portion followed by the addition of HCl in dioxane (3.5 mL, 4.0 M solution, 14 mmol). The reaction mixture was heated in a heating block at 100 °C overnight. After this time, LCMS showed full conversion of starting material. The reaction mixture was made basic with saturated NaHCO_3_ and extracted with ethyl acetate (3 × 30 mL). The combined organic layers were washed with water and finally with brine. The organic layer was dried over Na_2_SO_4_, filtered, and concentrated to obtain a crude product. The crude material was purified by silica gel chromatography using a gradient elution with (0.5–5%) methanol in ethyl acetate to obtain the pure product as a tan solid (960 mg, 76%). ^1^H NMR (DMSO-*d*_6_) δ 9.52 (br s, NH, exchanges with deuterium), 8.61 (d, *J* = 2.3 Hz, 1H), 8.20–8.00 (m, 2H), 7.77 (d, *J* = 9.0 Hz, 1H), 7.58 (dd, *J* = 9.1, 2.4 Hz, 1H), 7.37–7.26 (m, 2H), 7.11 (s, 1H), 6.93 (s, 2H), 6.59 (br s, NH_2_, exchanges with deuterium), 6.09 (br s, NH_2_, exchanges with deuterium). ^13^C NMR (DMSO-*d*_6_) δ 163.0, 162.6 (d, ^1^*J*_CF_ = 246.1 Hz), 161.9, 158.1, 153.5, 151.7, 145.1, 136.4 (d, ^4^*J*_CF_ = 3.0 Hz), 135.9, 129.8, 128.6 (d, ^3^*J*_CF_ = 8.4 Hz), 124.2, 117.9, 115.4 (d, ^2^*J*_CF_ = 21.3 Hz), 109.8, 99.3, 93.9. HRMS (ESI-TOF) calcd for C_19_H_13_N_6_ClF [M – H]^−^ 379.0874 *m*/*z*, found 379.0870. LC purity (254 nm) >98%.

#### Method A: General Procedure for the Synthesis of Compounds **8a**–**p**

Amine (0.27 mmol, 2.5 equiv) and *N*,*N*-diisopropylethylamine (0.21 mmol, 1.9 equiv) were added to a solution of pyrimidine chloride (**7**) (40 mg, 0.11 mmol, 1.0 equiv) in absolute ethanol (0.5 mL). The reaction was heated under microwave irradiation at 150 °C for 90 min, or until all starting material had been consumed. Ethanol was removed under reduced pressure, and the crude material was purified by preparative HPLC (MeCN/H_2_O (0.1% TFA)). The combined pure fractions were freeze-dried to yield the pure compound as the TFA-salts unless otherwise stated.

#### *N*^6^-(2-Amino-6-(piperazin-1-yl)pyrimidin-4-yl)-2-(4-fluorophenyl)quinoline-4,6-diamine (**8a**)

Prepared from piperazine (27 mg, 0.32 mmol) and **7** (48 mg, 0.13 mmol) using method A. The crude product was purified by preparative HPLC (MeCN/H_2_O (0.1% TFA)). All pure fractions were pooled and pH was set to basic, and the product was extracted with ethyl acetate. The combined organic layers were dried over Na_2_SO_4_, filtered, and concentrated to yield **8a** as a yellow solid (23 mg, 42%). ^1^H NMR (MeOD) δ 8.39 (d, *J* = 2.2 Hz, 1H), 8.03 (d, *J* = 9.1 Hz, 1H), 7.99–7.92 (m, 3H), 7.46–7.37 (m, 2H), 7.03 (s, 1H), 3.96–3.89 (m, 4H), 3.35–3.32 (m, 4H) C4-H in the pyrimidine ring exchanges with deuterium. ^13^C NMR (MeOD) δ 164.7 (d, ^1^*J*_CF_ = 252.1 Hz), 158.1, 155.6, 151.0, 136.4, 136.0, 129.9 (d, ^3^*J*_CF_ = 9.1 Hz), 129.6, 128.5 (d, ^4^*J*_CF_ = 3.3 Hz), 121.1, 116.2, 116.0 (d, ^2^*J*_CF_ = 22.5 Hz), 114.9, 100.4, 42.4, 41.1 (three carbons missing). HRMS (ESI-TOF) calcd for C_23_H_24_N_8_F [M + H]^+^ 431.2108 *m*/*z*, found 431.2106. LC purity (254 nm) >98%.

#### *N*^6^-(2-Amino-6-morpholinopyrimidin-4-yl)-2-(4-fluorophenyl) quinoline-4,6-diamine (**8b**)

Prepared from morpholine (110 μL, 1.26 mmol) and **7** (48 mg, 0.13 mmol) using method A to yield **8b** as a yellow powder (54 mg, 51%). ^1^H NMR (DMSO-*d*_6_ + D_2_O) δ 8.49 (s, 1H), 8.03 (d, *J* = 9.1 Hz, 1H), 7.99–7.87 (m, 3H), 7.56–7.45 (m, 2H), 7.00 (s, 1H), 3.66 (m, 4H), 3.50 (m, 4H) C4-H in the pyrimidine ring exchanges with deuterium. ^13^C NMR (DMSO-*d*_6_) δ 164.3 (d, ^1^*J*_CF_ = 250.8 Hz), 157.5, 155.5 149.9, 136.8, 135.8, 130.9 (d, ^3^*J*_CF_ = 9.1 Hz), 129.5, 128.8 (d, ^4^*J*_CF_ = 3.1 Hz), 121.7, 118.5, 116.7 (d, ^2^*J*_CF_ = 22.0 Hz), 116.1, 115.6, 100.7, 65.7, 45.0 (two carbons missing). HRMS (ESI-TOF) calcd for C_23_H_23_N_7_OF [M + H]^+^ 432.1948 *m*/*z*, found 432.1959. LC purity (254 nm) >98%.

#### *N*^6^-(2-Amino-6-(4-methylpiperazin-1-yl)pyrimidin-4-yl)-2-(4-fluorophenyl)quinoline-4,6-diamine (**8c**)

Prepared from 4-methylpiperazine (30 μL, 0.27 mmol) and **7** (40 mg, 0.11 mmol) using method A to yield **8c** as a yellow solid (31 mg, 37%). ^1^H NMR (DMSO-*d*_6_) δ 13.62 (br s, exchanges with D_2_O), 10.43 (br s, exchanges with D_2_O), 10.02 (br s, exchanges with D_2_O), 8.97 (br s, exchanges with D_2_O), 8.76 (s, 1H), 8.63 (br s, exchanges with D_2_O), 8.05 (d, *J* = 9.1 Hz, 1H), 8.03–7.97 (m, 2H), 7.91 (dd, *J* = 9.2, 2.2 Hz, 1H), 7.63–7.49 (m, 2H), 7.04 (s, 1H), 5.71 (s, 1H, exchanges with D_2_O), 4.42–4.22 (m, 2H), 3.63–3.41 (m, 2H), 3.41–3.16 (m, 2H), 3.16–3.00 (m, 2H), 2.86 (s, 3H). ^13^C NMR (DMSO-*d*_6_) δ 163.9 (d, ^1^*J*_CF_ = 250.2 Hz), 161.6, 159.4, 157.1, 149.0, 138.3, 134.8, 130.4 (d, ^3^*J*_CF_ = 9.1 Hz), 128.8 (d, ^4^*J*_CF_ = 3.1 Hz), 128.4, 121.0, 117.9, 116.2 (d, ^2^*J*_CF_ = 22.1 Hz), 116.0, 115.0 100.4, 76.4, 51.7, 42.1, 41.1. HRMS (ESI-TOF) calcd for C_24_H_26_N_8_F [M + H]^+^ 445.2264 *m*/*z*, found 445.2266. LC purity (254 nm) = 95%.

#### *N*^6^-(2-Amino-6-(4-isopropylpiperazin-1-yl)pyrimidin-4-yl)-2-(4-fluorophenyl)quinoline-4,6-diamine (**8d**)

Prepared from 4-isopropylpiperazine (38 μL, 0.27 mmol) and **7** (40 mg, 0.11 mmol) using method A to yield **8d** as a yellow solid (56 mg, 65%). ^1^H NMR (DMSO-*d*_6_) δ 13.62 (br s, exchanges with D_2_O), 10.09 (br s, exchanges with D_2_O), 9.99 (br s, exchanges with D_2_O), 8.97 (br s, exchanges with D_2_O), 8.78 (s, 1H), 8.61 (br s, exchanges with D_2_O), 8.05 (d, *J* = 9.2 Hz, 1H), 8.03–7.97 (m, 2H), 7.90 (dd, *J* = 9.2, 2.2 Hz, 1H), 7.62–7.53 (m, 2H), 7.04 (s, 1H), 5.70 (s, 1H, exchanges with D_2_O), 4.45–4.30 (m, 2H), 3.64–3.49 (m, 3H), 3.35–3.18 (m, 2H), 3.17–3.00 (m, 2H), 1.30 (d, *J* = 6.6 Hz, 6H). ^13^C NMR (DMSO-*d*_6_) δ 163.9 (d, ^1^*J*_CF_ = 250.3 Hz), 161.6, 159.5, 157.1, 148.9, 138.5, 134.7, 130.4 (d, ^3^*J*_CF_ = 9.1 Hz), 128.8 (d, ^4^*J*_CF_ = 3.1 Hz), 128.3, 121.0, 118.0, 116.2 (d, ^2^*J*_CF_ = 22.2 Hz), 116.0, 115.0, 100.4, 76.3, 57.2, 47.0, 41.1, 16.3. HRMS (ESI-TOF) calcd for C_26_H_30_N_8_F [M + H]^+^ 473.2577 *m*/*z*, found 473.2584. LC purity (254 nm) >98%.

#### 2-(4-(2-Amino-6-((4-amino-2-(4-fluorophenyl)quinolin-6-yl)amino)pyrimidin-4-yl)piperazin-1-yl)ethan-1-ol (**8e**)

Prepared from 2-(piperazin-1-yl)ethan-1-ol (33 μL, 0.26 mmol) and **7** (47 mg, 0.12 mmol) using method A to yield **8e** as a yellow solid (57 mg, 57%). ^1^H NMR (DMSO-*d*_6_) δ 13.62 (br s, exchanges with D_2_O), 9.99 (br s, exchanges with D_2_O), 8.97 (br s, exchanges with D_2_O), 8.79 (s, 1H), 8.60 (br s, exchanges with D_2_O), 8.04 (d, *J* = 9.1 Hz, 1H), 8.03–7.97 (m, 2H), 7.91 (dd, *J* = 9.2, 2.2 Hz, 1H), 7.61–7.53 (m, 2H), 7.05 (s, 1H), 5.70 (s, 1H, exchanges with D_2_O), 4.48–4.08 (m, 2H), 3.86–3.73 (m, 2H), 3.71–3.51 (m, 2H), 3.47–3.01 (m, 6H). ^13^C NMR (DMSO-*d*_6_) δ 163.9 (d, ^1^*J*_CF_ = 250.2 Hz), 161.4, 159.1, 157.2, 149.1, 138.1, 135.0, 130.4 (d, ^2^*J*_CF_ = 9.1 Hz), 128.8 (d, ^4^*J*_CF_ = 3.1 Hz), 128.5, 121.1, 118.1, 116.2 (d, ^2^*J*_CF_ = 22.1 Hz), 116.0, 115.1, 100.5, 76.3, 57.5, 54.8, 50.6, 40.9. HRMS (ESI-TOF) calcd for C_25_H_28_N_8_OF [M + H]^+^ 475.2370 *m*/*z*, found 445.2374. LC purity (254 nm) = 98%.

#### *N*^6^-(2-Amino-6-(4-(dimethylamino)piperidin-1-yl)pyrimidin-4-yl)-2-(4-fluorophenyl)quinoline-4,6-diamine (**8f**)

Prepared from *N*,*N*-dimethylpiperidin-4-amine (70 mg, 0.55 mmol) and **7** (38 mg, 0.10 mmol) using method A to yield **8f** as a yellow solid (56 mg, 69%). ^1^H NMR (DMSO-*d*_6_) δ 13.64 (br s, exchanges with D_2_O), 10.09 (br s, exchanges with D_2_O), 8.99 (br s, exchanges with D_2_O), 8.70 (s, 1H), 8.06 (d, *J* = 9.1 Hz, 1H), 8.04–7.97 (m, 2H), 7.91 (dd, *J* = 9.2, 2.2 Hz, 1H), 7.61–7.53 (m, 2H), 7.05 (s, 1H), 5.72 (s, 1H), 4.53–4.16 (m, 2H), 3.48 (m, 1H), 3.05–2.91 (m, 2H), 2.79 (d, *J* = 4.1 Hz, 6H), 2.16–2.05 (m, 2H), 1.68–1.50 (m, 2H). ^13^C NMR (DMSO-*d*_6_) δ 163.9 (d, ^1^*J*_CF_ = 250.3 Hz), 157.2, 149.2, 137.9, 135.1, 130.4 (d, ^3^*J*_CF_ = 9.1 Hz), 128.8 (d, ^4^*J*_CF_ = 3.2 Hz), 128.7, 121.2, 118.1, 116.2 (d, ^2^*J*_CF_ = 22.1 Hz), 116.0, 115.1, 100.5, 75.8, 62.1, 42.7, 39.2, 25.2 (two carbons missing). HRMS (ESI-TOF) calcd for C_26_H_30_N_8_F [M + H]^+^ 473.2577 *m*/*z*, found 473.2572. LC purity (254 nm) = 96%.

#### *tert*-Butyl ((1-(2-Amino-6-((4-amino-2-(4-fluorophenyl)quinolin-6-yl)amino)pyrimidin-4-yl)piperidin-4-yl)methyl)carbamate (**8g**)

Prepared from *tert*-butyl (piperidin-4-ylmethyl)carbamate (45 mg, 0.21 mmol) and **7** (40 mg, 0.11 mmol) using method A to yield **8g** as a yellow solid (47 mg, 57%). ^1^H NMR (MeOD) δ 8.45 (s, 1H), 8.09–7.86 (m, 4H), 7.45–7.36 (m, 2H), 7.02 (s, 1H), 4.34–4.02 (m, 2H), 3.13–2.92 (m, 4H), 1.91–1.68 (m, 3H), 1.44 (s, 9H), 1.35–1.14 (m, 2H) C4-H in the pyrimidine ring exchanges with deuterium. ^13^C NMR (DMSO-*d*_6_) δ 164.1 (d, ^1^*J*_CF_ = 250.2 Hz), 157.4, 155.8, 149.6, 137.2 (detected by heteronuclear multiple bond correlation (HMBC)), 135.5 (detected by HMBC), 130.7 (d, ^3^*J*_CF_ = 9.0 Hz), 128.9 (d, ^4^*J*_CF_ = 2.8 Hz), 121.5, 118.5, 116.5 (d, ^2^*J*_CF_ = 22.1 Hz), 116.1, 115.5, 112.5, 100.7, 77.5 (2C, two peaks overlapping), 45.0 (3C, two peaks overlapping), 35.9, 28.9, 28.3. HRMS (ESI-TOF) calcd for C_30_H_36_N_8_O_2_F [M + H]^+^ 559.2945 *m*/*z*, found 559.2933. LC purity (254 nm) = 98%.

#### *N*^6^-(2-Amino-6-(4-(aminomethyl)piperidin-1-yl)pyrimidin-4-yl)-2-(4-fluorophenyl)quinoline-4,6-diamine (**8h**)

Compound **8g** (25 mg, 0.032 mmol) was suspended in a 1:1 mixture of absolute ethanol and 4 M HCl in 1,4-dioxane. The reaction mixture was stirred at room temperature for 2 h. The solvent was removed under reduced pressure to yield the pure compound **8h** as a tan solid (23 mg, quantitative yield). ^1^H NMR (CDCl_3_/MeOD, 1:3) δ 8.67 (br s, 1H), 8.25–7.81 (m, 4H), 7.39–7.26 (m, 2H), 6.98 (s, 1H), 5.77 (br s, 1H), 4.30–3.97 (m, 2H), 3.20–2.96 (m, 2H), 2.84 (d, *J* = 6.5 Hz, 2H), 2.11–1.85 (m, 3H), 1.45–1.26 (m, 2H). ^13^C NMR (DMSO-*d*_6_) δ 164.1 (d, ^1^*J*_CF_ = 250.2 Hz), 157.4, 155.8, 149.6, 137.2 (detected by HMBC), 135.5 (detected by HMBC), 130.7 (d, ^3^*J*_CF_ = 9.0 Hz), 128.9 (d, ^4^*J*_CF_ = 2.8 Hz), 121.5, 118.5, 116.5 (d, ^2^*J*_CF_ = 22.1 Hz), 116.1, 115.5, 112.5, 100.7, 77.5 (2C, two peaks overlapping), 45.0 (3C, two peaks overlapping), 35.9, 28.9, 28.3. HRMS (ESI-TOF) calcd for C_25_H_28_N_8_F [M + H]^+^ 459.2421 *m*/*z*, found 459.2422. LC purity (254 nm) = 96%.

#### *tert*-Butyl (1-(2-Amino-6-((4-amino-2-(4-fluorophenyl)quinolin-6-yl)amino)pyrimidin-4-yl)piperidin-4-yl)carbamate (**8i**)

Prepared from *tert*-butyl piperidin-4-ylcarbamate (79 mg, 0.39 mmol) and **7** (67 mg, 0.17 mmol) using method A to yield **8i** as a yellow solid (40 mg, 30%). ^1^H NMR (MeOD) δ 8.44 (s, 1H), 8.04–7.91 (m, 4H), 7.47–7.36 (m, 2H), 7.02 (s, 1H), 4.23–4.06 (m, 2H), 3.65 (m, 1H), 3.26–3.08 (m, 2H), 2.03–1.90 (m, 2H), 1.56–1.39 (m, 11H). ^13^C NMR (DMSO-*d*_6_) δ 164.1 (d, ^1^*J*_CF_ = 250.2 Hz), 157.4, 155.0, 149.5, 130.8 (d, ^3^*J*_CF_ = 9.1 Hz), 129.1–128.9 (m, 2C), 121.6, 116.6 (d, ^2^*J*_CF_ = 22.1 Hz), 116.2, 100.7, 77.9, 47.1, 43.7, 31.3, 28.3 (seven carbons missing). HRMS (ESI-TOF) calcd for C_29_H_34_N_8_O_2_F [M + H]^+^ 545.2789 *m*/*z*, found 545.2778. LC purity (254 nm) = 95%.

#### *N*^6^-(2-Amino-6-(4-aminopiperidin-1-yl)pyrimidin-4-yl)-2-(4-fluorophenyl)quinoline-4,6-diamine (**8j**)

Prepared from 4-aminopiperidine (23 mg, 0.22 mmol) and **7** (38 mg, 0.10 mmol) using method A to yield **8j** as a yellow solid (42 mg, 53%). ^1^H NMR (DMSO-*d*_6_) δ 13.65 (br s, exchanges with D_2_O), 10.16 (br s, exchanges with D_2_O), 9.01 (br s, exchanges with D_2_O), 8.69 (s, 1H), 8.11 (br s, exchanges with D_2_O), 8.07 (d, *J* = 9.1 Hz, 1H), 8.04–7.97 (m, 2H), 7.91 (dd, *J* = 9.1, 2.2 Hz, 1H), 7.65–7.50 (m, 2H), 7.05 (s, 1H), 5.71 (s, 1H), 4.25–4.11 (m, 2H), 3.37 (m, 1H), 3.15–2.98 (m, 2H), 2.07–1.94 (m, 2H), 1.60–1.40 (m, 2H). ^13^C NMR (DMSO-*d*_6_) δ 164.0 (d, ^1^*J*_CF_ = 250.4 Hz), 157.3, 149.4, 137.2, 135.4, 130.5 (d, ^3^*J*_CF_ = 9.1 Hz), 128.9, 128.7 (d, ^4^*J*_CF_ = 3.1 Hz), 121.3, 118.2, 116.3 (d, ^2^*J*_CF_ = 22.1 Hz), 115.9, 115.3, 100.5, 75.8, 46.9, 42.8, 28.8. Two carbons missing. HRMS (ESI-TOF) calcd for C_24_H_26_N_8_F [M + H]^+^ 445.2264 *m*/*z*, found 445.2258. LC purity (254 nm) >98%.

#### *N*^4^-(4-Amino-2-(4-fluorophenyl)quinolin-6-yl)-*N*^6^,*N*^6^-dimethylpyrimidine-2,4,6-triamine (**8k**)

Compound **7** (40 mg, 0.11 mmol) was added to a solution of KOH (29 mg, 0.53 mmol) in 10 mL of dimethylformamide (DMF)/water (1:1). The reaction mixture was heated in a sealed microwave vial (Biotage 10–20 mL) under microwave heating at 150 °C for 90 min. Ethanol was removed under reduced pressure and the crude material was purified by preparative HPLC (MeCN/H_2_O (0.1% TFA)). The combined pure fractions were freeze-dried to yield the TFA salt of the pure compound **8k** as a yellow solid (15 mg, 23%). ^1^H NMR (400 MHz, DMSO-*d*_6_) δ 13.56 (br s, exchanges with D_2_O), 10.04 (br s, exchanges with D_2_O), 8.94 (br s, 1H), 8.73 (br s, exchanges with D_2_O), 8.05 (d, *J* = 9.1 Hz, 1H), 8.03–7.97 (m, 2H), 7.91 (dm, *J* = 9.1 Hz, 1H), 7.63–7.51 (m, 2H), 7.02 (s, 1H), 5.50 (s, 1H), 3.09 (s, 6H). ^13^C NMR (DMSO-*d*_6_) δ 164.0 (d, ^1^*J*_CF_ = 250.2 Hz), 157.3, 149.5 (detected by heteronuclear single quantum coherence (HSQC)), 138.3 (detected by HMBC), 136.1 (detected by HMBC), 130.7 (d, ^3^*J*_CF_ = 9.1 Hz), 129.0-128.8 (m), 121.5, 118.6, 116.5 (d, ^2^*J*_CF_ = 22.1 Hz), 116.0, 100.6, 74.9, 37.8 (three carbons missing). HRMS (ESI-TOF) calcd for C_21_H_21_N_7_F [M + H]^+^ 390.1842 *m*/*z*, found 390.1858. LC purity (254 nm) = 96%.

#### 2-((2-Amino-6-((4-amino-2-(4-fluorophenyl)quinolin-6-yl)amino)pyrimidin-4-yl)(methyl)amino)ethan-1-ol (**8l**)

Prepared from 2-(methylamino)-ethanol (48 mg, 0.63 mmol) and **7** (38 mg, 0.10 mmol) using method A and heating for 2.5 h to yield **8l** as a yellow solid (35 mg, 54%). ^1^H NMR (MeOD) δ 8.49 (br s, 1H), 8.04–7.88 (m, 4H), 7.45–7.33 (m, 2H), 7.02 (s, 1H), 3.80 (t, *J* = 5.2 Hz, 2H), 3.71–3.60 (m, 2H), 3.17 (s, 3H). ^13^C NMR (DMSO-*d*_6_) δ 164.5 (d, ^1^*J*_CF_ = 250.0 Hz), 157.8, 149.9, 138.1 (detected by HMBC), 136.0 (detected by HMBC), 131.1 (d, ^3^*J*_CF_ = 9.1 Hz), 129.4, 121.9, 117.0 (d, ^2^*J*_CF_ = 22.1 Hz), 116.5, 101.1, 75.7, 58.7, 52.7, 37.6 (five carbons missing). HRMS (ESI-TOF) calcd for C_22_H_23_N_7_OF [M + H]^+^ 420.1948 *m*/*z*, found 420.1935. LC purity (254 nm) = 97%.

#### *N*^4^-(4-Amino-2-(4-fluorophenyl)quinolin-6-yl)-*N*^6^-methyl-*N*^6^-(2-(methylamino)ethyl)pyrimidine-2,4,6-triamine (**8m**)

Prepared from *N*,*N*-dimethylethyleneamine (93.5 mg, 1.05 mmol) and **7** (40 mg, 0.105 mmol) using method A and heating for 3.0 h to yield **8m** as a yellow solid (46 mg, 57%). ^1^H NMR (400 MHz, DMSO-*d*_6_) δ 8.93 (s, NH, exchanges with deuterium), 8.58 (d, *J* = 2.2 Hz, 1H), 8.25 (s, 1H), 8.02 (dd, *J* = 8.7, 5.7 Hz, 2H), 7.64 (d, *J* = 9.0 Hz, 1H), 7.49 (dd, *J* = 8.9, 2.2 Hz, 1H), 7.24 (t, *J* = 8.9 Hz, 2H), 7.02 (s, 1H), 6.43 (br s, 2H, exchanges with deuterium), 6.11 (br s, 2H, exchanges with deuterium), 5.32 (br s, 1H, exchanges with deuterium), 3.60 (s, 2H), 2.96 (s, 2H), 2.85 (s, 3H), 2.49 (s, 3H). ^13^C NMR (101 MHz, DMSO-*d*_6_) δ 164.21, 162.99 (d, *J*_CF_ = 123.1 Hz), 162.81, 161.77, 153.12, 151.89, 144.76, 138.15, 136.98 (d, *J*_CF_ = 2.9 Hz), 129.86, 128.96 (d, *J*_CF_ = 8.3 Hz), 124.43, 118.56, 115.78 (d, *J*_CF_ = 21.3 Hz), 108.53, 99.56, 76.08, 47.99, 46.24, 36.28, 33.63. HRMS (ESI-TOF) calcd for C_23_H_25_N_8_F [M + H]^+^ 432.2186 *m*/*z*, found 432.2182. LC purity (254 nm) >98%.

#### (1-(2-Amino-6-((4-amino-2-(4-fluorophenyl)quinolin-6-yl)amino)pyrimidin-4-yl)piperidin-4-yl)methanol (**8n**)

Prepared from 4-piperidylmethanol (24.2 mg, 0.210 mmol) and **7** (40 mg, 0.105 mmol) using method A and heating for 3.0 h to yield **8n** as a yellow solid (40 mg, 55%). ^1^H NMR (400 MHz, DMSO-*d*_6_) δ 8.90 (s, NH, exchanges with deuterium), 8.70 (d, *J* = 2.2 Hz, 1H), 8.16 (s, 1H), 8.07 (dd, *J* = 8.7, 5.7 Hz, 2H), 7.73 (d, *J* = 9.0 Hz, 1H), 7.56 (dd, *J* = 9.0, 2.2 Hz, 1H), 7.33 (t, *J* = 8.8 Hz, 2H), 7.06 (s, 1H), 6.73 (br s, 2H, exchanges with deuterium), 6.09 (br s, 2H, exchanges with deuterium), 5.44 (br s, 1H, exchanges with deuterium), 4.21 (d, *J* = 13.1 Hz, 1H), 3.26 (d, *J* = 5.9 Hz, 2H), 2.74 (t, *J* = 12.7 Hz, 2H), 1.68 (m, 3H), 1.08 (m, 2H). ^13^C NMR (101 MHz, DMSO-*d*_6_) δ 164.38, 163.78, 163.63, 163.07 (d, *J*_CF_ = 112.2 Hz), 161.93, 152.53 (d, *J*_CF_ = 4.5 Hz), 143.35, 138.59, 136.08, 129.22 (d, *J*_CF_ = 8.3 Hz), 128.77, 124.81, 118.34, 115.91 (d, *J*_CF_ = 21.5 Hz), 108.29, 99.71, 76.33, 66.24, 44.22, 39.27, 28.63. HRMS (ESI-TOF) calcd for C_25_H_26_N_7_FO [M + H]^+^ 460.2183 *m*/*z*, found 460.2185. LC purity (254 nm) >98%.

#### 1-(2-Amino-6-((4-amino-2-(4-fluorophenyl)quinolin-6-yl)amino)pyrimidin-4-yl)piperidin-3-ol (**8o**)

Prepared from 3-hydroxypiperidine (21.2 mg, 0.210 mmol) and **7** (40 mg, 0.105 mmol) using method A and heating for 3.0 h to yield **8o** as a yellow solid (40 mg, 56%). ^1^H NMR (400 MHz, DMSO-*d*_6_) δ 8.88 (s, NH, exchanges with deuterium), 8.65 (d, *J* = 2.3 Hz, 1H), 8.09 (s, 1H), 7.99 (dd, *J* = 8.7, 5.7 Hz, 2H), 7.69 (d, *J* = 9.0 Hz, 1H), 7.52 (dd, *J* = 9.1, 2.3 Hz, 1H), 7.29 (t, *J* = 8.9 Hz, 2H), 6.99 (s, 1H), 6.85 (br s, 2H, exchanges with deuterium), 6.04 (br s, 2H, exchanges with deuterium), 5.38 (s, 1H, exchanges with deuterium), 3.97 (m, 2H), 3.36 (dt, *J* = 9.3, 4.8 Hz, 1H), 2.75 (td, *J* = 10.5, 5.1 Hz, 1H), 2.57 (dd, *J* = 12.5, 9.3 Hz, 1H), 1.84 (dd, *J* = 9.0, 5.1 Hz, 1H), 1.61 (dd, *J* = 10.7, 5.4 Hz, 1H), 1.29 (td, *J* = 10.4, 10.0, 5.7 Hz, 2H). ^13^C NMR (101 MHz, DMSO-*d*_6_) δ 164.51, 163.67, 163.03 (d, *J*_CF_ = 116.0 Hz), 163.01, 162.06, 153.05, 152.15, 138.80, 135.42, 129.40 (d, *J*_CF_ = 8.4 Hz), 128.03, 125.14, 118.16, 116.01 (d, *J*_CF_ = 21.5 Hz), 108.32, 99.82, 76.25, 65.62, 51.65, 44.06, 34.16, 23.26. HRMS (ESI-TOF) calcd for C_24_H_24_N_7_FO [M + H]^+^ 446.2026 *m*/*z*, found 446.2022. LC purity (254 nm) >98%.

#### 1-(2-Amino-6-((4-amino-2-(4-fluorophenyl)quinolin-6-yl)amino)pyrimidin-4-yl)piperidin-4-ol (**8p**)

Prepared from 4-hydroxypiperidine (34 mg, 0.336 mmol) and **7** (40 mg, 0.105 mmol) using method A and heating for 3.0 h to yield **8p** as a yellow solid (39 mg, 67%). ^1^H NMR (400 MHz, DMSO-*d*_6_) δ 8.86 (s, NH, exchanges with deuterium), 8.63 (d, *J* = 2.3 Hz, 1H), 8.09 (s, 1H), 8.00 (m, 2H), 7.67 (d, *J* = 9.0 Hz, 1H), 7.51 (dd, *J* = 9.1, 2.3 Hz, 1H), 7.27 (t, *J* = 8.9 Hz, 1H), 6.99 (s, 1H), 6.72 (br s, 2H, exchanges with deuterium), 6.04 (br s, 2H, exchanges with deuterium), 5.39 (s, 1H, exchanges with deuterium), 3.85 (dt, *J* = 13.6, 4.5 Hz, 2H), 3.62 (dt, *J* = 8.8, 4.6 Hz, 1H), 2.96 (ddd, *J* = 13.2, 10.0, 3.1 Hz, 2H), 1.68 (dq, *J* = 8.0, 3.9 Hz, 2H), 1.25 (ddt, *J* = 13.9, 9.1, 4.5 Hz, 2H). ^13^C NMR (101 MHz, DMSO-*d*_6_) δ 164.42, 163.76, 163.09, 163.04 (d, *J*_CF_ = 100.3 Hz), 161.97, 152.56 (d, *J*_CF_ = 28.0 Hz), 143.09, 138.63, 135.88, 129.28 (d, *J*_CF_ = 8.4 Hz), 128.55, 124.91, 118.28, 115.94 (d, *J*_CF_ = 21.5 Hz), 108.31, 99.75, 76.28, 66.78, 41.98, 34.26. HRMS (ESI-TOF) calcd for C_24_H_24_N_7_FO [M + H]^+^ 446.2026 *m*/*z*, found 446.2024. LC purity (254 nm) >98%.

#### *N*^6^-(2-Amino-6-ethoxypyrimidin-4-yl)-2-(4-fluorophenyl)quinoline-4,6-diamine (**9a**)

Compound **7** (40 mg, 0.11 mmol) and KOH (30 mg, 0.54 mmol) were suspended in absolute ethanol (0.5 mL). The reaction was heated under microwave irradiation at 150 °C for 30 min. Ethanol was removed under reduced pressure and the crude was purified by preparative HPLC. The combined pure fractions were freeze-dried to yield the pure compound **9a** as a yellow solid (8 mg, 13%). ^1^H NMR (DMSO-*d*_6_) δ 13.53 (br s, exchanges with D_2_O), 9.84 (br s, exchanges with D_2_O), 8.92 (d, *J* = 2.2 Hz, 1H), 8.43 (br s, exchanges with D_2_O), 8.04–7.97 (m, 3H), 7.91 (dd, *J* = 9.2, 2.2 Hz, 1H), 7.61–7.53 (m, 2H), 7.02 (s, 1H), 5.58 (s, 1H, exchanges with D_2_O), 4.27 (q, *J* = 7.0 Hz, 2H), 1.32 (t, *J* = 7.0 Hz, 3H). ^13^C NMR (DMSO-*d*_6_) δ 168.0, 164.1 (d, ^1^*J*_CF_ = 250.0 Hz), 162.3, 160.7, 157.2, 149.1, 138.9, 134.7, 130.7 (d, ^3^*J*_CF_ = 9.0 Hz), 129.0 (d, ^4^*J*_CF_ = 3.1 Hz), 128.2, 121.1, 116.5 (d, ^2^*J*_CF_ = 22.1 Hz), 116.0, 109.8, 100.6, 78.5, 62.4, 14.5. HRMS (ESI-TOF) calcd for C_21_H_20_N_6_OF [M + H]^+^ 391.1683 *m*/*z*, found 391.1681. LC purity (254 nm) = 98%.

#### 2-((2-Amino-6-((4-amino-2-(4-fluorophenyl)quinolin-6-yl)amino)pyrimidin-4-yl)oxy)ethan-1-ol (**9b**)

Compound **7** (33 mg, 0.09 mmol) and KOH (24 mg, 0.43 mmol) were suspended in ethyleneglycol (0.70 mL). The reaction was heated under microwave irradiation at 100–120 °C for 70 min. The crude reaction mixture was purified by preparative HPLC. The combined pure fractions were freeze-dried to yield the pure compound **9b** as a tan solid (35 mg, 63%). ^1^H NMR (DMSO-*d*_6_) δ 13.50 (br s, 1H), 9.74 (br s, 1H), 8.93 (d, *J* = 2.2 Hz, 1H), 8.87 (br s, 1H), 8.41 (br s, 1H), 8.04–7.94 (m, 3H), 7.91 (dd, *J* = 9.2, 2.2 Hz, 1H), 7.62–7.51 (m, 2H), 7.01 (s, 1H), 5.58 (s, 1H), 4.33–4.12 (m, 2H), 3.72–3.68 (m, 2H). ^13^C NMR (DMSO-*d*_6_) δ 168.1, 164.0 (d, ^1^*J*_CF_ = 250.0 Hz), 162.1, 160.6, 157.2, 149.0, 138.8, 134.7, 130.6 (d, ^3^*J*_CF_ = 9.1 Hz), 129.0, 128.1, 121.1, 116.5 (d, ^2^*J*_CF_ = 22.1 Hz), 116.0, 109.7, 100.5, 78.5, 68.3, 59.2. HRMS (ESI-TOF) calcd for C_21_H_20_N_6_O_2_F [M + H]^+^ 407.1626 *m*/*z*, found 407.1644. LC purity (254 nm) = 97%.

#### *N*^6^-(2-Amino-6-((1-methylpiperidin-3-yl)methoxy)pyrimidin-4-yl)-2-(4-fluorophenyl)quinoline-4,6-diamine (**9c**)

Compound **7** (50 mg, 0.13 mmol) and KOH (37 mg, 0.66 mmol) were suspended in 1-methyl-3-piperidinemethanol (0.70 mL). The reaction was heated under microwave irradiation at 100 °C for 2.5 h. The crude reaction mixture was purified by preparative HPLC. The combined pure fractions were freeze-dried to yield the pure compound **9c** as a yellow solid (62 mg, 58%). ^1^H NMR (DMSO-*d*_6_) δ 13.55 (br s, exchanges with deuterium), 9.69 (br s, exchanges with deuterium), 8.95 (d, *J* = 2.2 Hz, 1H), 8.89 (br s, exchanges with deuterium), 8.39 (br s, exchanges with deuterium), 8.06–7.90 (m, 4H), 7.64–7.51 (m, 2H), 7.03 (s, 1H), 6.75 (br s, exchanges with D_2_O), 5.55 (s, 1H), 4.22 (dd, *J* = 10.7, 4.9 Hz, 1H), 4.05 (dd, *J* = 10.7, 7.3 Hz, 1H), 3.58–3.37 (m, 2H), 2.97–2.73 (m, 5H), 2.23 (m, 1H), 1.99–1.61 (m, 3H), 1.25 (m, 1H). ^13^C NMR (DMSO-*d*_6_) δ 169.5, 164.0 (d, ^1^*J*_CF_ = 249.7 Hz), 162.4, 162.3, 157.1, 148.7, 139.5, 134.2, 130.6 (d, ^3^*J*_CF_ = 9.0 Hz), 129.0 (d, ^4^*J*_CF_ = 3.1 Hz), 127.8, 121.0, 116.5 (d, ^2^*J*_CF_ = 22.1 Hz), 116.0, 108.7, 100.5, 78.8, 66.6, 55.7, 53.6, 43.1, 34.1, 24.0, 22.2. HRMS (ESI-TOF) calcd for C_26_H_29_N_7_OF [M + H]^+^ 474.2418 *m*/*z*, found 474.2415. LC purity (254 nm) >98%.

#### 2-Amino-6-chloropyrimidin-4(3*H*)-one (**11**)

Prepared following a literature procedure^26^ to yield **11** as a white solid (178 mg, 90%). ^1^H NMR (DMSO-*d*_6_) δ 7.13 (br s, 2H), 5.58 (s, 1H).

#### 2-Amino-6-((4-amino-2-(4-fluorophenyl)quinolin-6-yl)amino)pyrimidin-4(3*H*)-one (**12**)

4 M HCl (aq) in dioxane (0.08 mL, 0.32 mmol) was added to a suspension of diamine (**6**) (80 mg, 0,32 mmol) and 2-amino-6-chloro-3*H*-pyrimidin-4-one (**11**) (92 mg, 0.63 mmol) in NMP (0.5 mL) in a microwave vial (Biotage 2–5 mL). The vial was capped, and the reaction mixture was heated at 100 °C overnight. The reaction mixture was poured into saturated NaHCO_3_ (aq), and the precipitate was collected by filtration and washed with water and ethyl acetate to yield **12**, a brown solid (43 mg, 38%). ^1^H NMR (DMSO-*d*_6_) δ 8.82 (s, 1H), 8.32 (s, 1H), 8.22–8.04 (m, 2H), 7.74 (d, *J* = 9.0 Hz, 1H), 7.55 (dd, *J* = 9.2, 1.6 Hz, 1H), 7.39–7.23 (m, 2H), 7.10 (s, 1H), 6.68 (br s, 2H), 6.57 (s, 2H), 4.92 (s, 1H). ^13^C NMR (DMSO-*d*_6_) δ 163.6, 162.6 (d, ^1^*J*_CF_ = 245.2 Hz), 162.3, 155.8, 153.3, 151.6, 145.0, 136.8, 136.5 (d, ^4^*J*_CF_ = 3.0 Hz), 129.6, 128.6 (d, ^3^*J*_CF_ = 8.3 Hz), 125.1, 118.0, 115.3 (d, ^2^*J*_CF_ = 21.4 Hz), 110.3, 99.1, 78.8. HRMS (ESI-TOF) calcd for C_19_H_16_N_6_OF [M + H]^+^ 363.1370 *m*/*z*, found 363.1374. LC purity (254 nm) = 97%.

#### *N*^6^-(6-Chloropyrimidin-4-yl)-2-(4-fluorophenyl)quinoline-4,6-diamine (**14**)

Diamine **6** (100 mg, 0.390 mmol) and 4,6-dichloropyrimidine (118 mg, 0.790 mmol) were suspended in *N*-methylpyrrolidone (0.5 mL) and HCl (0.10 mL, 4 M in dioxane, 0.40 mmol) in a microwave vial (Biotage, 2–5 mL). The vial was capped, and the reaction mixture was stirred at 100 °C for 6 h in a heating block. At the end of this time, the solvent was removed under reduced pressure and the crude product was adsorbed over silica, then loaded onto the column; elution with ethyl acetate/pentane (1:1, 200 mL) removed most of the *N*-methylpyrrolidone. Then, the column was run with a gradient of 0–5% methanol/ethyl acetate. NMR showed that the purified compound still contained *N*-methylpyrrolidone. The compound was purified one more time using 0–10% methanol/ethyl acetate + triethylamine (0.1%) to yield the product **14** as a brown solid (43 mg, 30%). ^1^H NMR (DMSO-*d*_6_) δ 10.04 (s, 1H), 8.51 (m, 1H), 8.25 (d, *J* = 2.3 Hz, 1H), 8.16–8.10 (m, 2H), 7.85 (d, *J* = 9.0 Hz, 1H), 7.73 (dd, *J* = 9.0, 2.3 Hz, 1H), 7.40–7.27 (m, 2H), 7.12 (s, 1H), 6.86 (m, 1H), 6.74 (br s, 2H). ^13^C NMR (DMSO-*d*_6_) δ 162.7 (d, ^1^*J*_CF_ = 245.5 Hz), 161.6, 158.6, 158.2, 154.3, 152.0, 146.0, 136.3 (d, ^4^*J*_CF_ = 2.8 Hz), 134.1, 129.9, 128.7 (d, ^3^*J*_CF_ = 8.4 Hz), 125.4, 117.9, 115.4 (d, ^2^*J*_CF_ = 21.4 Hz), 113.1, 104.2, 99.2. HRMS (ESI-TOF) calcd for C_19_H_14_N_5_ClF [M + H]^+^ 366.0922 *m*/*z*, found 366.0927. LC purity (254 nm) >98%.

#### 2-(4-Fluorophenyl)-*N*^6^-(6-(piperazin-1-yl)pyrimidin-4-yl)quinoline-4,6-diamine (**15**)

Compound **14** (31 mg, 0.085 mmol) and piperazine (15 mg, 0.17 mmol) were suspended in absolute ethanol (2 mL) followed by the addition of *N*,*N*-diisopropylethylamine (30 μL, 0.17 mmol) in a microwave vial (Biotage 2–5 mL). The vial was capped, and the reaction mixture was heated using microwave irradiation at 150 °C for 90 min. Ethanol was removed under reduced pressure, and the crude material was purified by preparative HPLC (MeCN/H_2_O (0.1% TFA)). The combined pure fractions were freeze-dried to yield the TFA salt of the pure product **15** as a yellow solid (38 mg, 59%). ^1^H NMR (DMSO-*d*_6_) δ 13.50 (s, 1H), 9.76 (s, 1H), 8.94 (s, 2H), 8.57 (d, *J* = 2.0 Hz, 1H), 8.36 (s, 1H), 8.09–7.96 (m, 4H), 7.63–7.53 (m, 2H), 6.98 (s, 1H), 6.21 (s, 1H), 3.79–3.75 (m, 4H), 3.31–3.12 (m, 4H). ^13^C NMR (DMSO-*d*_6_) δ 164.0 (d, ^1^*J*_CF_ = 250.0 Hz), 161.8, 160.2, 157.4, 156.9, 149.1, 138.5, 134.8, 130.6 (d, ^3^*J*_CF_ = 9.0 Hz), 129.0 (d, ^4^*J*_CF_ = 3.1 Hz), 128.7, 121.0, 116.5 (d, ^2^*J*_CF_ = 22.1 Hz), 116.1, 111.32, 100.4, 85.1, 42.3, 40.7. HRMS (ESI-TOF) calcd for C_23_H_23_N_7_F [M + H]^+^ 416.1999 *m*/*z*, found 416.2014. LC purity (254 nm) = 95%.

#### 2-(4-Fluorophenyl)-6-nitroquinoline (**18**)

Potassium carbonate (914 mg, 6.62 mmol), water (1 mL), and tetrakis triphenylphosphine palladium (128 mg, 0.110 mmol) were added to a solution of 2-chloro-6-nitroquinoline (460 mg, 2.21 mmol) and (4-fluorophenyl)boronic acid (370 mg, 2.65 mmol) in 1,2-dimethoxyethane (8 mL) under a nitrogen atmosphere. The reaction vial (Biotage 10–20 mL) was sealed and the mixture was stirred at 150 °C for 0.5 h using microwave irradiation. The reaction solution was allowed to return to room temperature, water was added, and the solution was extracted with ethyl acetate. The organic phase was dried over MgSO_4_, filtered, and the solvent was removed under reduced pressure. The residue was purified by silica gel column chromatography (100% dichloromethane) to obtain the title product **18** as a pale yellow solid (590 mg, 85%). ^1^H NMR (CDCl_3_) δ 8.79 (dd, *J* = 2.5, 0.4 Hz, 1H), 8.49 (dd, *J* = 9.3, 2.5 Hz, 1H), 8.39 (ddd, *J* = 8.7, 0.8, 0.4 Hz, 1H), 8.26–8.21 (m, 3H), 8.01 (d, *J* = 8.7 Hz, 1H), 7.44–7.16 (m, 2H). ^13^C NMR (CDCl_3_) δ 164.6 (d, ^1^*J*_CF_ = 251.2 Hz), 159.6, 150.5, 145.4, 138.7, 134.7 (d, ^4^*J*_CF_ = 3.1 Hz), 131.4, 130.0 (d, ^3^*J*_CF_ = 8.8 Hz), 125.9, 124.5, 123.4, 120.4, 116.3 (d, ^2^*J*_CF_ = 21.8 Hz). HRMS (ESI-TOF) calcd for C_15_H_10_N_2_O_2_F [M + H]^+^ 269.0726 *m*/*z*, found 269.0723. LC purity (254 nm) = 98%.

#### 2-(4-Fluorophenyl)quinolin-6-amine (**19**)

To a solution of **18** (0.500 g, 1.86 mmol) in MeOH/EtOAc (30 mL, 1:1) was added 10% palladium on charcoal (50 mg) under nitrogen atmosphere, and the mixture was evacuated and filled with hydrogen gas (repeated three times). Then, the reaction mixture was hydrogenated under hydrogen atmosphere at room temperature for 8 h. Pd/C was filtered off over Celite with suction, and the filtrate was evaporated under reduced pressure. The crude product (yield was quantitative) was used in the next step without any further purification. ^1^H NMR (CDCl_3_) δ 8.13–8.06 (m, 2H), 7.98–7.92 (m, 2H), 7.70 (d, *J* = 8.6 Hz, 1H), 7.22–7.14 (m, 3H), 6.91 (d, *J* = 2.6 Hz, 1H), 3.96 (br s, 2H). ^13^C NMR (CDCl_3_) δ 163.5 (d, ^1^*J*_CF_ = 248.0 Hz), 153.0, 144.7, 143.5, 136.3 (d, ^4^*J*_CF_ = 3.1 Hz), 134.8, 131.0, 129.0 (d, ^3^*J*_CF_ = 8.2 Hz), 128.7, 121.9, 119.1, 115.8 (d, ^2^*J*_CF_ = 21.7 Hz), 107.5. HRMS (ESI-TOF) calcd for C_15_H_12_N_2_F [M + H]^+^ 239.0985 *m*/*z*, found 239.0980. LC purity (254 nm) >98%.

#### 6-Chloro-*N*^4^-(2-(4-fluorophenyl)quinolin-6-yl)pyrimidine-2,4-diamine (**20**)

Compound **19** (300 mg, 1.26 mmol) was suspended in *N*-methylpyrrolidone (1 mL) and 2-amino-4,6-dichloropyrimidine (230 mg, 1.39 mmol) was added, followed by careful addition of HCl in dioxane (0.17 mL, 4.0 M, 5.0 mmol). The reaction mixture was stirred at 110 °C for 24 h. The reaction was cooled to room temperature, and the yellow solid was filtered off. The solid was extracted with EtOAc and saturated NaHCO_3_ solution, dried over MgSO_4_, filtered, and concentrated under reduced pressure to yield **20** as a yellow solid (460 mg, 79%). ^1^H NMR (DMSO-*d*_6_) δ 9.71 (br s, NH, exchanges with deuterium), 8.67 (d, *J* = 2.4 Hz, 1H), 8.36–8.28 (m, 3H), 8.11 (d, *J* = 8.7 Hz, 1H), 7.97 (d, *J* = 9.1 Hz, 1H), 7.85 (dd, *J* = 9.1, 2.5 Hz, 1H), 6.90 (br s, NH_2_, exchanges with deuterium), 6.13 (s, 1H). ^13^C NMR (DMSO-*d*_6_) δ 163.0 (d, ^1^*J*_CF_ = 246.6 Hz), 162.9, 161.7, 158.3, 153.0, 143.8, 138.1, 136.4, 135.3 (d, ^4^*J*_CF_ = 3.0 Hz), 129.4, 129.1 (d, ^3^*J*_CF_ = 8.5 Hz), 127.6, 124.3, 118.7, 115.7 (d, ^2^*J*_CF_ = 21.4 Hz), 114.5, 94.4. HRMS (ESI-TOF) calcd for C_19_H_14_N_5_ClF [M + H]^+^ 366.0922 *m*/*z*, found 366.0926. LC purity (254 nm) >98%.

#### *N*^4^-(2-(4-Fluorophenyl)quinolin-6-yl)-6-(piperazin-1-yl)pyrimidine-2,4-diamine (**21**)

Prepared from piperazine (28 mg, 0.33 mmol) and **20** (48 mg, 0.13 mmol) using method A to yield **21** as an orange solid (20 mg, 42%). ^1^H NMR (DMSO-*d*_6_) δ 8.43 (d, *J* = 8.7 Hz, 1H), 8.32–8.26 (m, 2H), 8.16–8.06 (m, 3H), 7.75 (dd, *J* = 9.0, 2.5 Hz, 1H), 7.42–7.34 (m, 2H), 5.67 (s, 1H, C4-H in the pyrimidine ring exchanges with deuterium), 3.78–3.73 (m, 4H), 3.21–3.16 (m, 4H). ^13^C NMR (DMSO-*d*_6_) δ 163.3 (d, ^1^*J*_CF_ = 247.1 Hz), 155.2, 154.2, 144.7, 137.1, 135.8, 134.9 (d, ^4^*J*_CF_ = 2.9 Hz), 130.1, 129.4 (d, ^3^*J*_CF_ = 8.6 Hz), 127.5, 126.0, 119.1, 118.4, 115.8 (d, ^2^*J*_CF_ = 21.5 Hz), 75.3, 42.2, 41.7 (two carbons missing). HRMS (ESI-TOF) calcd for C_23_H_23_N_7_F [M + H]^+^ 416.1999 *m*/*z*, found 416.2017. LC purity (254 nm) >98%.

#### *N*-(6-Chloropyrimidin-4-yl)-2-(4-fluorophenyl)quinolin-6-amine (**22**)

Compound **19** (300 mg, 1.26 mmol) was suspended in *N*-methylpyrrolidone (1 mL) and 4,6-dichloropyrimidine (210 mg, 1.39 mmol) was added, followed by careful addition of HCl in dioxane (0.17 mL, 4.0 M, 5.0 mmol). The reaction mixture was stirred at 110 °C for 24 h. The reaction was cooled to room temperature, and the yellow solid was filtered off. The solid was extracted with EtOAc and saturated NaHCO_3_ solution, dried over MgSO_4_, filtered, and concentrated under reduced pressure. The crude mixture was purified by column chromatography (5% MeOH in dichloromethane) to yield **22** as a yellow solid (440 mg, 71%). ^1^H NMR (DMSO-*d*_6_) δ 10.27 (s, 1H, exchanges with deuterium), 8.60 (d, *J* = 0.8 Hz, 1H), 8.47–8.40 (m, 2H), 8.35–8.26 (m, 2H), 8.12 (d, *J* = 8.7 Hz, 1H), 8.07 (d, *J* = 9.1 Hz, 1H), 7.93 (dd, *J* = 9.1, 2.4 Hz, 1H), 7.44–7.33 (m, 2H), 6.95 (d, *J* = 0.9 Hz, 1H). ^13^C NMR (DMSO-*d*_6_) δ 163.2 (d, ^1^*J*_CF_ = 247.1 Hz), 161.1, 158.5, 158.1, 153.4, 143.6, 137.2 (two peaks overlapping, detected by HSQC and HMBC), 134.7 (d, ^4^*J*_CF_ = 2.3 Hz), 129.4 (d, ^3^*J*_CF_ = 8.6 Hz), 129.2, 127.4, 125.0, 119.1, 115.8 (d, ^2^*J*_CF_ = 21.5 Hz), 115.4, 105.8. HRMS (ESI-TOF) calcd for C_19_H_13_N_4_ClF [M + H]^+^ 351.0813 *m*/*z*, found 351.0808. LC purity (254 nm) >98%.

#### 2-(4-Fluorophenyl)-*N*-(6-(piperazin-1-yl)pyrimidin-4-yl)quinolin-6-amine (**23**)

Prepared from piperazine (29 mg, 0.33 mmol) and **22** (48 mg, 0.13 mmol) using method A to yield **23** as a yellow solid (30 mg, 51%). ^1^H NMR (DMSO-*d*_6_ + drop D_2_O) δ 8.44 (d, *J* = 8.7 Hz, 1H), 8.39 (s, 1H), 8.32 (d, *J* = 2.4 Hz, 1H), 8.29–8.22 (m, 2H), 8.09 (d, *J* = 8.7 Hz, 1H), 8.06 (d, *J* = 9.1 Hz, 1H), 7.89 (dd, *J* = 9.1, 2.4 Hz, 1H), 7.42–7.34 (m, 2H), 6.19 (s, 1H), 3.84–3.75 (m, 4H), 3.25–3.15 (m, 4H). ^13^C NMR (DMSO-*d*_6_) δ 163.1 (d, ^1^*J*_CF_ = 246.6 Hz), 161.5, 160.1, 156.6, 152.9, 143.4, 138.3, 136.8, 134.9 (d, ^4^*J*_CF_ = 2.8 Hz), 129.2 (two signals overlapping, detected by HSQC), 127.7, 125.0, 118.9, 115.7 (d, ^2^*J*_CF_ = 21.6 Hz), 114.4, 85.2, 42.3, 40.8. HRMS (ESI-TOF) calcd for C_23_H_22_N_6_F [M + H]^+^ 401.1890 *m*/*z*, found 401.1907. LC purity (254 nm) >98%.

#### Production of NDH-2s and Assays

Details of cloning, expression, and purification are provided in the Supporting Information. Briefly, an *Ms*NDH-2 construct with a C-terminal His_6_-tag was expressed in *E. coli* C43(DE3) and purified by TALON metal-affinity chromatography in 8 mM 3-[(3-cholamidopropyl)dimethylammonio]-1-propanesulfonate hydrate (CHAPS). *Mt*NDH-2 with a C-terminal His_6_-tag was expressed in *M. smegmatis* strain mc^2^4517 (the kind gift of Prof. William Jacobs, Albert Einstein College of Medicine) using a T7-promoter-based vector pYUB28b, and purified using TALON resin. The protein was solubilized and purified with 2 and 0.25% BigCHAP, respectively.

NDH-2 oxidizes NADH in the presence of hydrogen acceptor quinones, *e.g.*, menadione, allowing the reaction to be followed spectrophotometrically as a decrease in absorbance at 340 nm. The linear changes observed with an enzyme concentration of 1.25 nM for *Mt*NDH-2 and 50 nM for *Ms*NDH-2, corresponding to substrate reduction, were monitored at 22 °C for 0.5 and 2 h, respectively. For inhibition tests, the enzyme was preincubated with the compound for 10 min at 22 °C, after which the reaction was started by adding 50 μM menadione. Reaction rates were measured as a function of inhibitor concentration (highest concentration 20 or 100 μM, depending on compound solubility), and half-maximal inhibitory concentration (IC_50_) values were determined by a nonlinear regression analysis of the sigmoidal dose–response curves in GraphPad Prism (GraphPad Software, Inc., La Jolla, CA). Polymyxin B was used as a positive control in the assay. The measured IC_50_s, 2.5 μg/mL for *Ms*NDH-2 and 0.41 μg/mL for *Mt*NDH-2, were similar to the value published earlier, 1.6 μg/mL for *Ms*NDH-2.^16^

#### Evaluation of Minimum Inhibitory Concentration (MIC) on ESKAPE Pathogens

Compound prepared in MHII medium was dispensed into a 96-well round-bottom microtiter plate to give final assay concentrations from 128 μg/mL down to 0.25 μg/mL (twofold dilution series in 10 wells, plus two control wells: medium control with no bacteria or compound, and growth control with bacteria added but no compound). Bacteria prepared from fresh colonies (grown on nonselective agar, incubation 18–24 h at 35 ± 2 °C) were suspended in saline to 0.5 McFarland (≅1.5 × 10^8^ CFU/mL). This bacterial suspension (50 μL) was transferred to 10 mL of MHII broth to give a final bacterial concentration ≅5 × 10^5^ CFU/mL (acceptable range (3–7 × 10^5^) CFU/mL). The 50 μL bacterial suspension was pipetted into each well (except medium control well, where 50 μL of MHII was pipetted). The final volume in each well was 100 μL. Plates were covered and incubated without shaking for 16–20 h at 35 ± 2 °C. MIC was read visually, as complete inhibition of growth by the unaided eye, using the medium-only wells as the control.

#### Evaluation of Minimum Inhibitory Concentration (MIC) on *M. tuberculosis* by Resazurin Reduction Microplate Assay (REMA)

Twofold serial dilutions of each test compound were prepared in 96-well plates. Frozen aliquots of replicating tubercle bacilli (reference strain H37Rv) were thawed and diluted to an optical density at 600 nm (OD600) of 0.0001 (3 × 10^4^ cells/mL) and added to the plates to obtain a total volume of 100 μL. Plates were incubated for 6 days at 37 °C before the addition of resazurin (0.025% [w/v] to 1/10 of well volume). After overnight incubation, the fluorescence of the resazurin metabolite, resorufin, was determined (by excitation at 560 nm and emission at 590 nm, measured using a TECAN infinite M200 microplate reader). The MIC was defined visually as the last concentration preventing resazurin turnover from blue to pink and confirmed by the level of fluorescence measured by the microplate reader. MIC was determined using GraphPad Prism version 7.0 software (GraphPad Software, Inc., La Jolla, CA). The experiment was performed twice, and all of the compounds were tested in duplicate. Rifampicin (MIC = 0.0003 μM) and bedaquiline (MIC = 0.37 μM) were used as reference antitubercular agents.

#### Evaluation of the Cytotoxicity of the HepG2 Cell Line

HepG2 cells were cultured in FBS-free medium and were then seeded into 96-well plates in 200 μL of complete culture medium at a final concentration of 5 × 10^4^ cells/well and treated by rifampicin, or by compounds (at a range of concentrations) for 72 h. Redox status was estimated using resazurin reduction (0.025% [w/v] to 1/10 of well volume). The resazurin assay was performed with a fluorometric method according to the procedure described previously for REMA assay. Three hours before the end of incubation, 10 μL of resazurin/well were added, yielding a final concentration of 10% resazurin. Plates were returned to the incubator, and the fluorescence was read after 6 h. The plates were exposed to an excitation wavelength of 535 nm, and emission at 580 nm was recorded on a TECAN infinite M200 microplate reader. The percent viability was expressed as fluorescence emitted by treated cells compared to control (medium or vehicle only). Compounds were run once in the primary screen; repeated evaluation is typically performed only if relevant potency and selectivity are shown. For these compounds, retesting was not done in view of the nonspecificity, and so error estimates cannot be provided.

#### Evaluation of Cytotoxicity on the MRC-5 Cell Line

MRC-5_SV2_ cells were cultured in Earl’s MEM + 5% FCSi. Assays were performed in 96-well microtiter plates, each well containing about 10^4^ cells/well. After 3 days of incubation with or without compounds, cell viability was assessed fluorimetrically after the addition of resazurin (λ_ex_ 550 nm, λ_em_ 590 nm). The results were expressed as % reduction in cell growth/viability compared to untreated control wells and CC_50_ was determined. Testing/retesting strategy was as described above for HepG2 cells.

#### Resistant Mutants and Whole Genome Sequencing

Mutants of *E. coli* Δ*tolC* resistant to compounds **8a** and **8g**, wild-type *P. aeruginosa* resistant to compound **8j**, and *A. baumannii* resistant to compound **9a** were selected by serial passage of independent lineages in Luria broth (LB) at increasing concentrations of compound (up to 4× initial MIC). At the end-point of selection, a single clone was isolated from each resistant culture for sequence analysis, by streaking for single colonies on LB agar and measuring MIC against the selective compound. Mutations were identified by whole-genome sequencing of independently selected mutants with increased MIC values. Genomic DNA for whole-genome sequencing was prepared using the MasterPure DNA Purification Kit (Epicentre, Illumina, Inc., Madison, Wisconsin). Final DNA was resuspended in EB buffer. Genomic DNA concentrations were measured in a Qubit 2.0 Fluorometer (Invitrogen *via* Thermo Fisher Scientific). DNA was diluted to 0.2 ng/mL in water (Sigma-Aldrich, Sweden), and the samples were prepared for whole genome sequencing according to the Nextera XT DNA Library Preparation Guide (Illumina, Inc., Madison, Wisconsin). After the polymerase chain reaction (PCR) clean-up step, the samples were validated for DNA fragment size distribution using the Agilent High Sensitivity D1000 ScreenTape System (Agilent Technologies, Santa Clara, California). Sequencing was performed using a MiSeq desktop sequencer, according to the manufacturer’s instructions (Illumina, Inc., Madison, Wisconsin). The sequencing data were aligned and analyzed in CLC Genomics Workbench version 8.0.3 (CLCbio, Qiagen, Denmark).

#### Multivariate Modeling

The multivariate modeling was performed in Simca, version 13.0.0.0, Umetrics AB (www.umetrics.com). The analyses (principle component analysis (PCA) and PLS) were made using negative logarithms of the MIC, TD99, TD50, and CC50 values; all variables were scaled to unit variance and centered. As MIC values in Table 1 were greater than the maximum concentration tested, twice this value is used in the analyses, to provide more guidance for the PCA/PLS analysis. The following molecular descriptors were used for the PLS modeling: molecular weight (MW), polar surface area (PSA), octanol/water partition coefficient (log *P*), number of hydrogen-bond donors and acceptors of the pyrimidine substituent (#HBD and #HBA), and the total charge of the compound, assuming that all aliphatic amines are protonated as well as all 4-amino quinolones (charge). The PSA and log *P* values were calculated using Instant JChem, version 17.1.9.0.

## Supporting Information Available

NMR spectra and LC chromatograms; cloning, expression, and purification of the NDH-2 proteins; activity assay; IC_50_ determination; minimum inhibitory concentration (MIC) assays on parasites; and quantitative SAR (QSAR) modeling (PDF)

## Abbreviations

*Mt*NDH-2: *M. tuberculosis* type II NADH-dehydrogenase
*Ms*NDH-2: *M. smegmatis* type II NADH-dehydrogenase
IC_50_: half-maximal inhibitory concentration
MIC: minimum inhibitory concentration
SAR: structure–activity relationship
