# Supplementary material for: Synthesis and In Vitro Biological Evaluation of Quinolinyl Pyrimidines Targeting Type II NADH-Dehydrogenase (NDH-2)
Source: ACS Infect Dis. 2022 Feb 21;8(3):482–98. doi: 10.1021/acsinfecdis.1c00413 (PMC8922281; doi:10.1021/acsinfecdis.1c00413)
Supplement: Supplementary file 1 — id1c00413_si_001.pdf [file id1c00413_si_001.pdf]

Supporting Information

Synthesis and *in vitro* biological evaluation of quinolinyl pyrimidines targeting type II NADH-dehydrogenase (NDH-2)

Lu Lu[a,#], Linda Åkerbladh[b,#], Shabbir Ahmad[a,#], Vivek Konda[b], Sha Cao[c], Anthony Vocat[d], Louis Maes[e], Stewart T. Cole[d], Diarmaid Hughes[c], Mats Larhed[f], Peter Brandt[b], Anders Karlén[b,]*, Sherry L. Mowbray[a,g] *

[a] Uppsala University, Department of Cell and Molecular Biology, BMC, Box 596, SE-751 24 Uppsala, Sweden[b] Uppsala University, Department of Medicinal Chemistry, Organic Pharmaceutical Chemistry, BMC, Box 574, SE-751 23 Uppsala, Sweden. [c] Uppsala University, Department of Medical Biochemistry and Microbiology, BMC, Box 582, SE-751 23 Uppsala, Sweden. [d] Global Health Institute, Ecole Polytechnique Fédérale de Lausanne, EPFL SV / GHI / UPCOL, Station nº19, CH-1015 Lausanne, Switzerland. [e] University of Antwerp, Faculty of Pharmaceutical, Biomedical and Veterinary Sciences, Laboratory of Microbiology, Parasitology and Hygiene, CDE-S7.27 Universiteitsplein 1, B-2610 Antwerp – Belgium. [f] Uppsala University, Department of Medicinal Chemistry, Science for Life Laboratory, BMC, Box 574, SE-751 23 Uppsala, Sweden. [g] Uppsala University, Department of Cell and Molecular Biology, Science for Life Laboratory, BMC, Box 596, SE-751 24 Uppsala, Sweden

[#]Lu Lu, Linda Åkerbladh and Shabbir Ahmad contributed equally to this work.

AUTHOR INFORMATION

* Corresponding authors at: Department of Cell and Molecular Biology, Uppsala University, BMC Box 596, SE-751 24 Uppsala, Sweden (S.L. Mowbray) and Department of Medicinal

Chemistry, Organic Pharmaceutical Chemistry, Uppsala University, BMC Box 574, SE-751 23 Uppsala, Sweden (A. Karlén).

Table of Contents

1. Chemistry 4

1.1 NMR spectra and LC chromatograms 4

2. Biology 42

2.1. *In vitro* biology: Determination of half maximal inhibitor concentration (IC_{50}) 42

2.1.1. Cloning, expression and purification of the NDH-2 proteins. 42

2.1.2. Activity assay. 44

2.1.3. IC_{50} determination 45

2.2. In vitro biology: Minimum inhibitory concentration (MIC) assays on parasites 45

3. QSAR Modeling 47

4. References 47

1. Chemistry

1.1 NMR spectra and LC chromatograms

N*6-(2-Amino-6-m-tolylpyrimidin-4-yl)-2-(4-fluorophenyl)quinoline-4,6-diamine (**1**).*

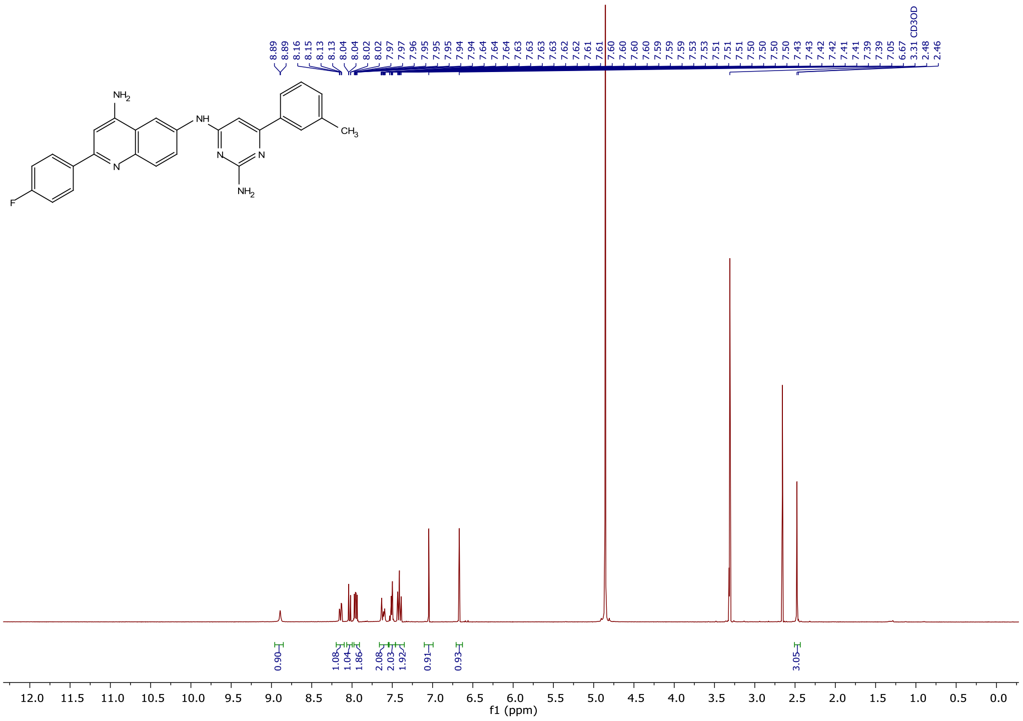

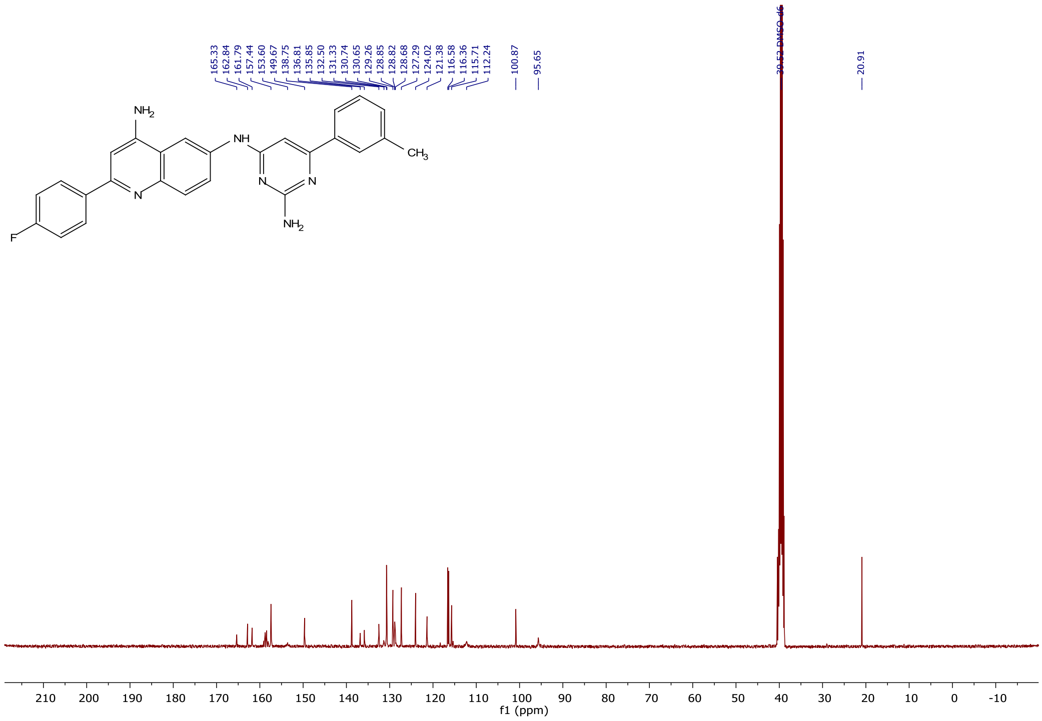

*4-Chloro-2-(4-fluorophenyl)-6-nitroquinoline (**4**).*

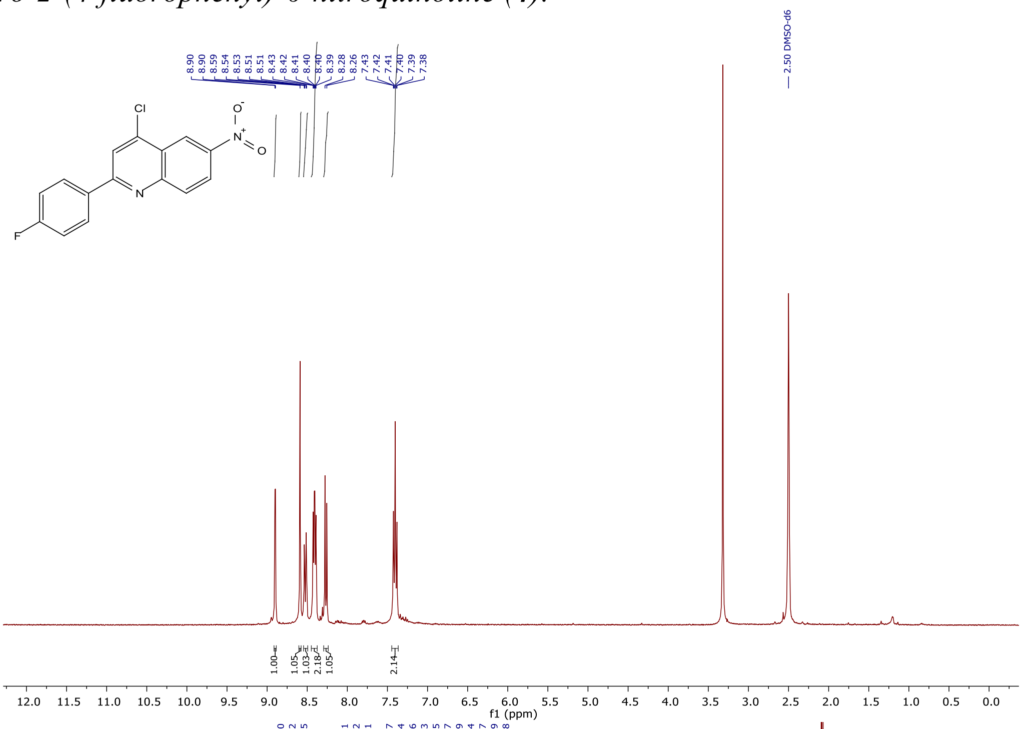

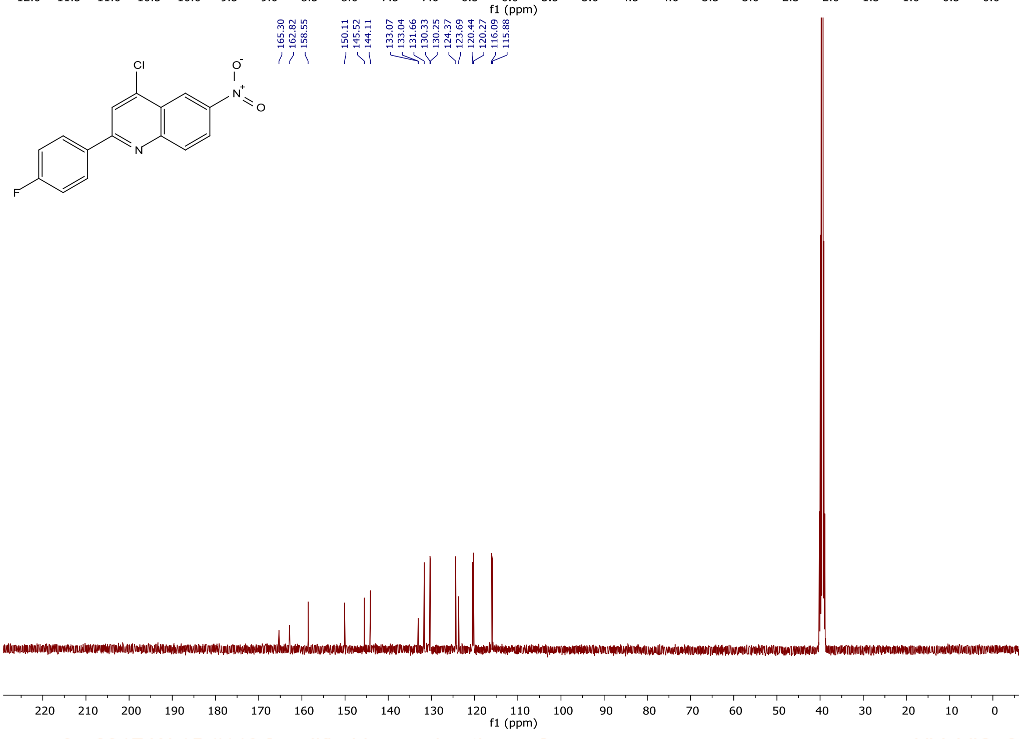

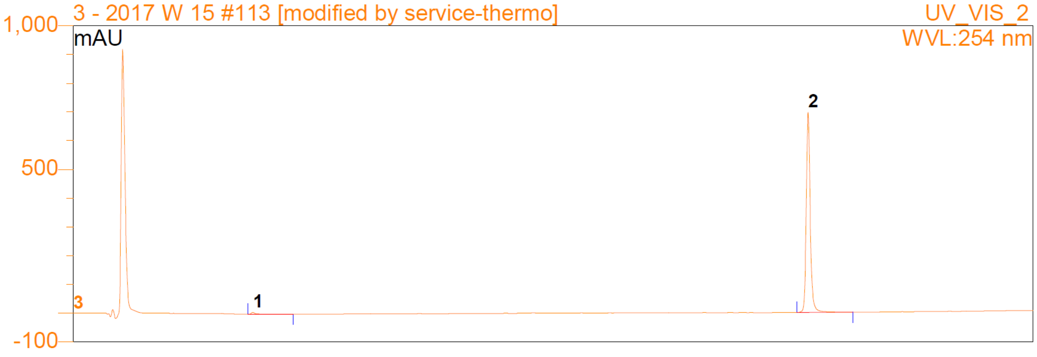

*2-(4-Fluorophenyl)quinoline-4,6-diamine (**6**).*

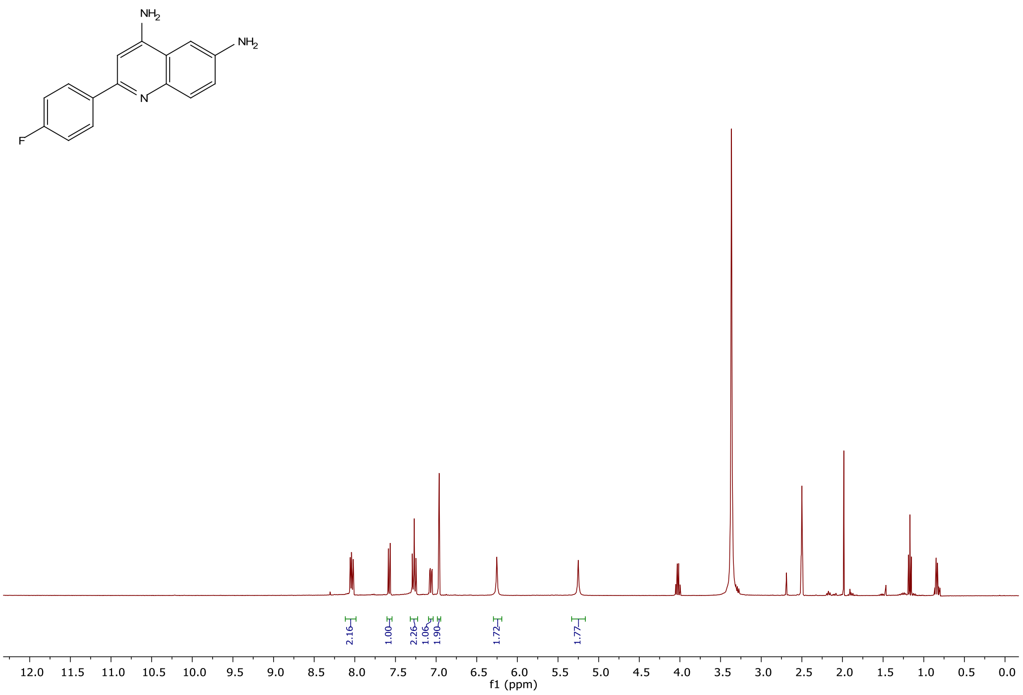

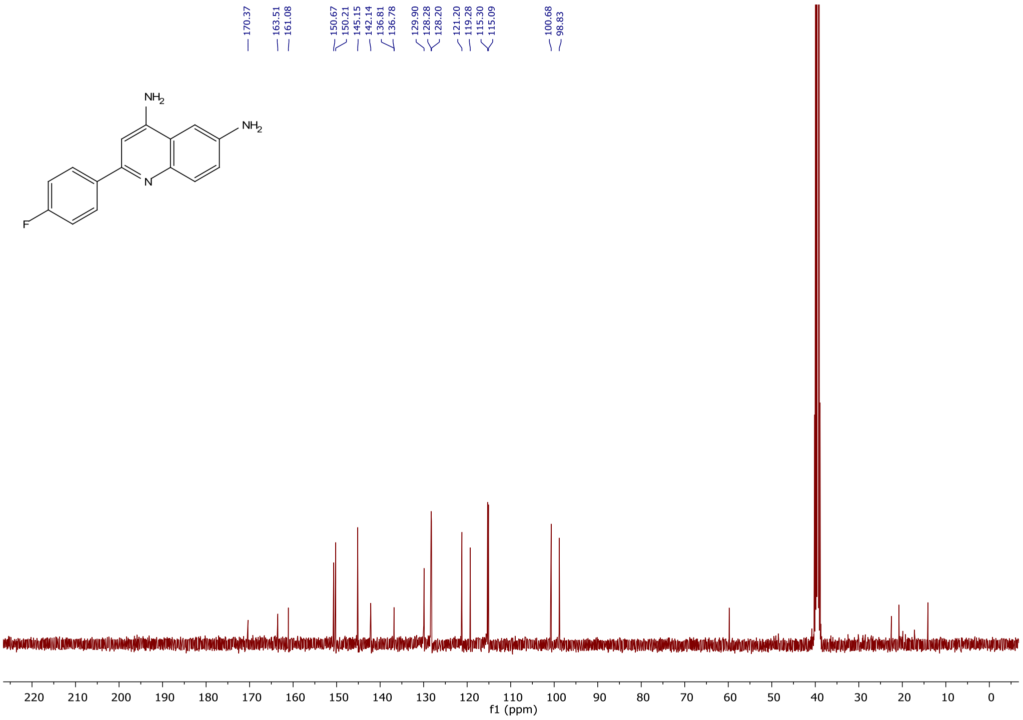

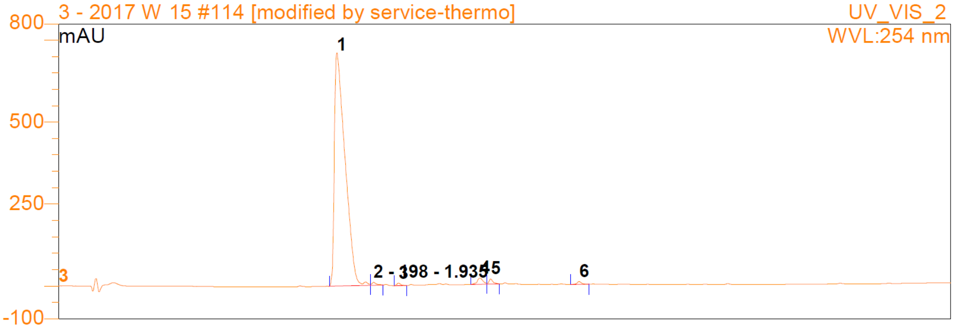

N6-(2-Amino-6-chloropyrimidin-4-yl)-2-(4-fluorophenyl)quinoline-4,6-diamine (7).

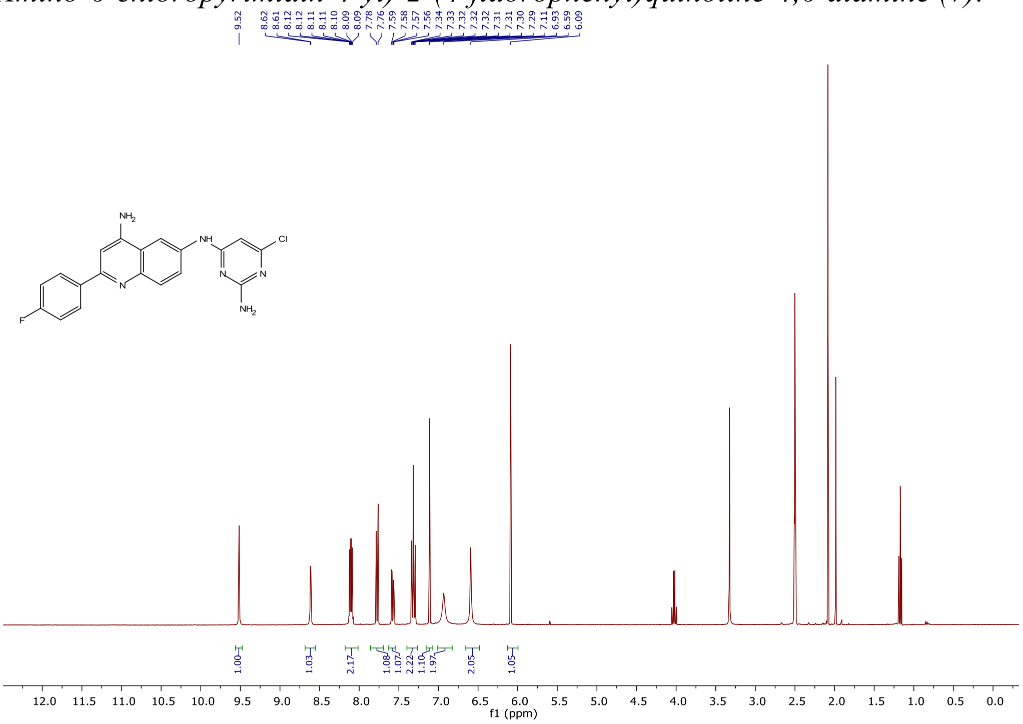

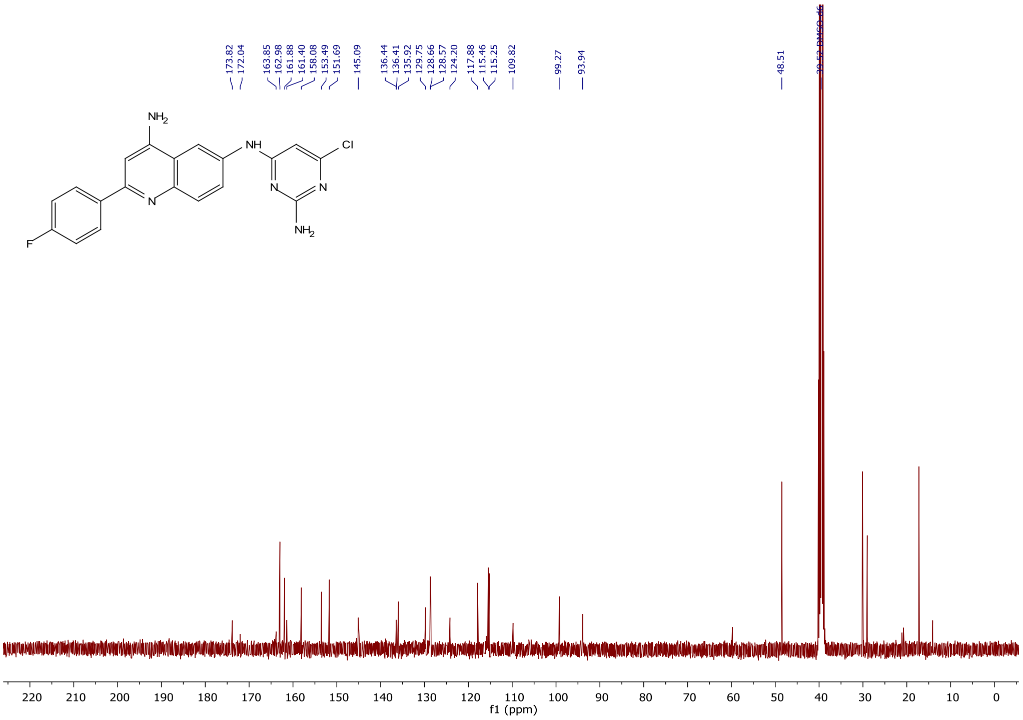

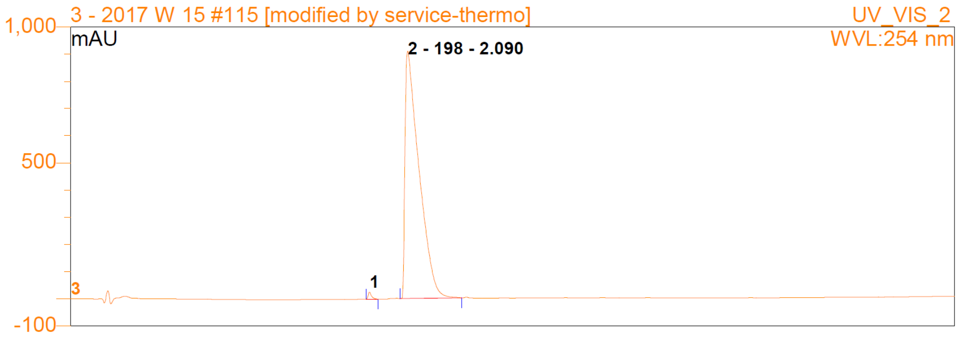

N6-*(2-Amino-6-(piperazin-1-yl)pyrimidin-4-yl)-2-(4-fluorophenyl)quinoline-4,6-diamine* ***(8a)***.

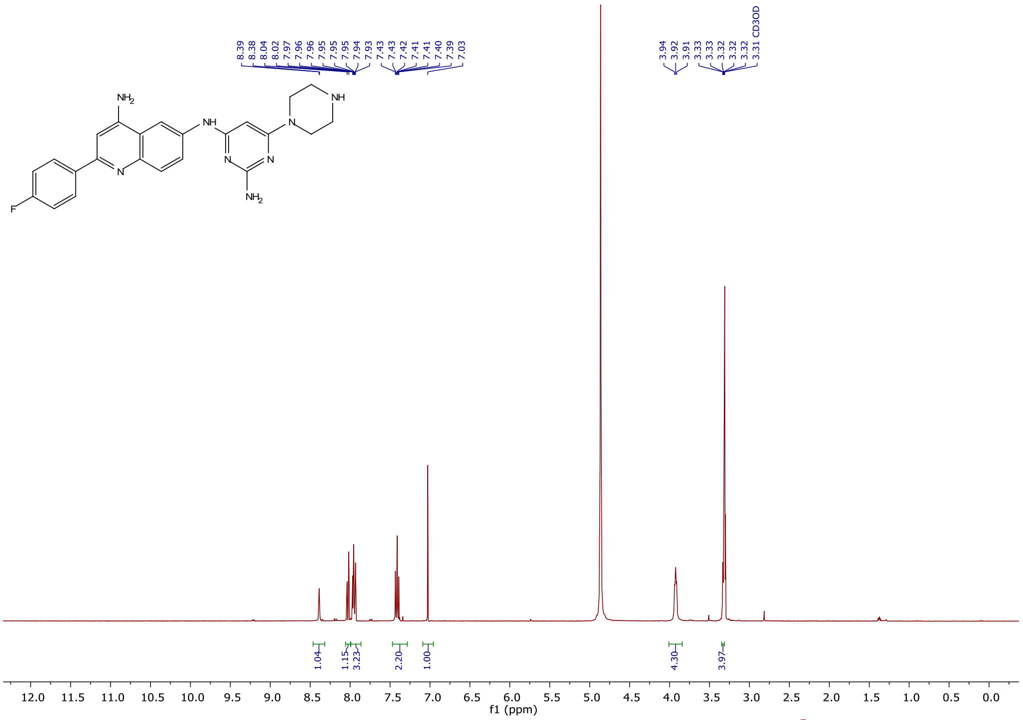

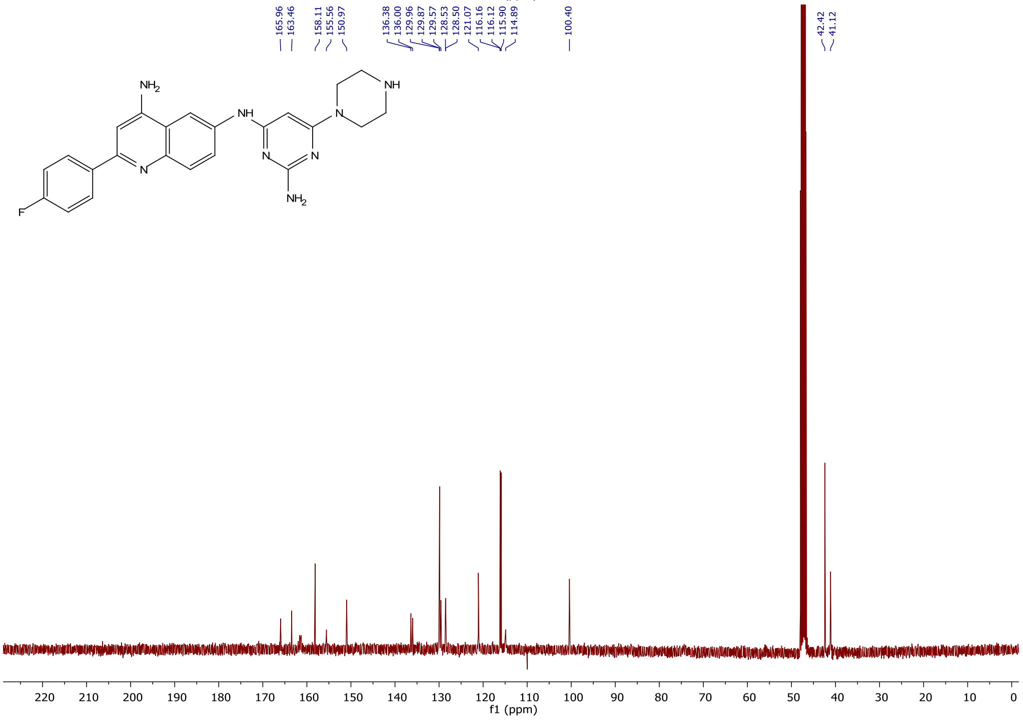

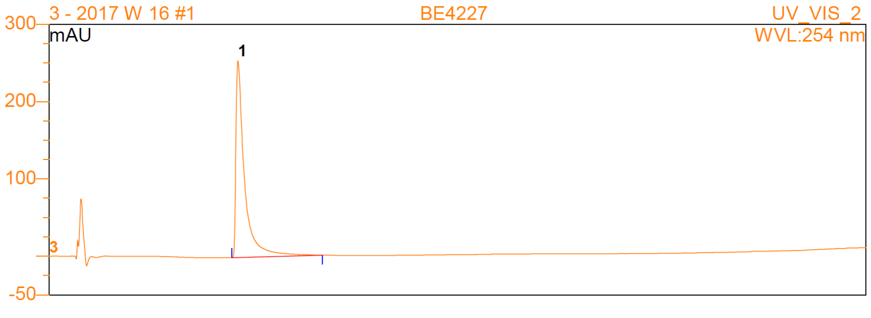

N6-*(2-Amino-6-morpholinopyrimidin-4-yl)-2-(4-fluorophenyl) quinoline-4,6-diamine* (***8b***).

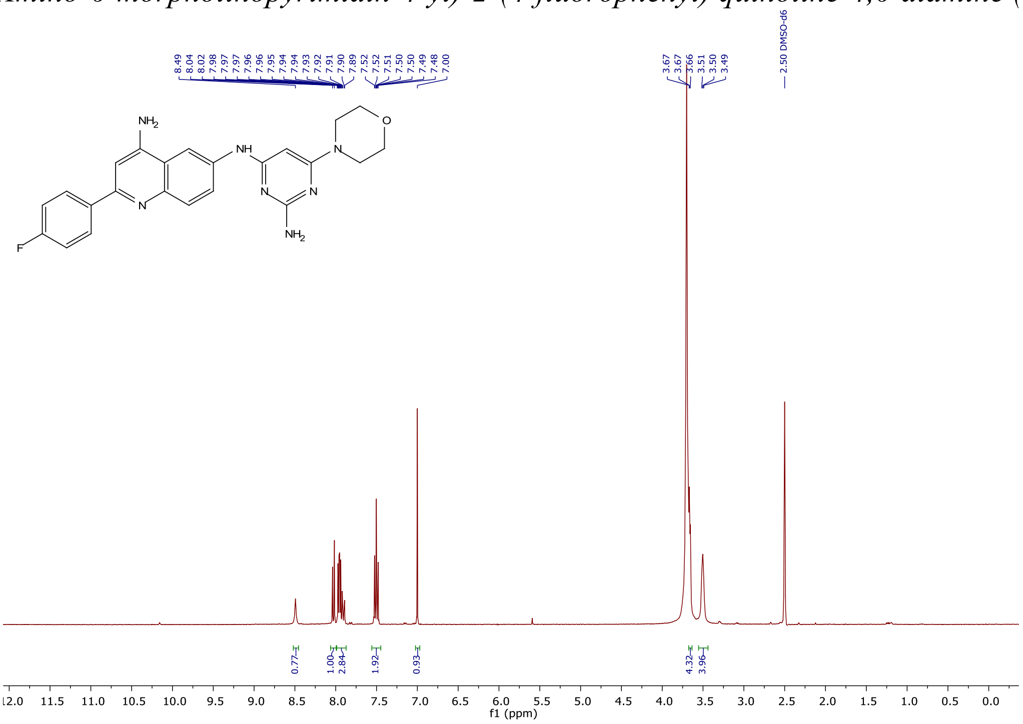

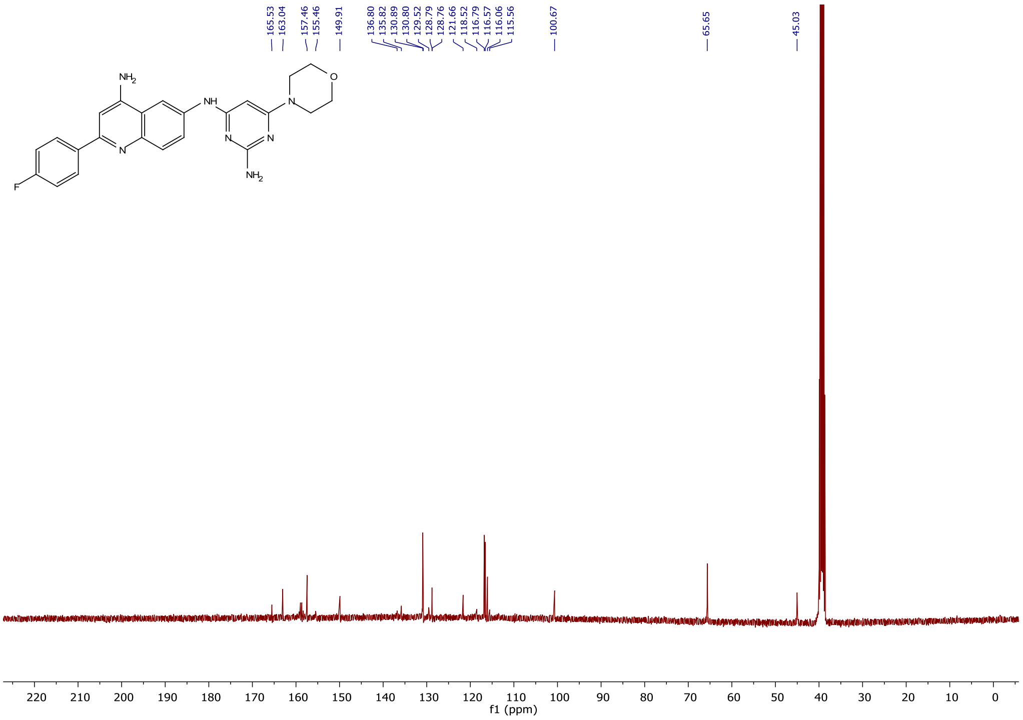

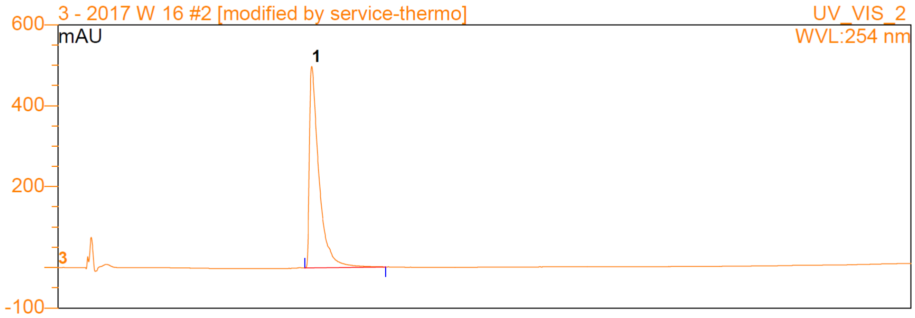

N*6-(2-Amino-6-(4-methylpiperazin-1-yl)pyrimidin-4-yl)-2-(4-fluorophenyl)quinoline-4,6-diamine (***8c***).*

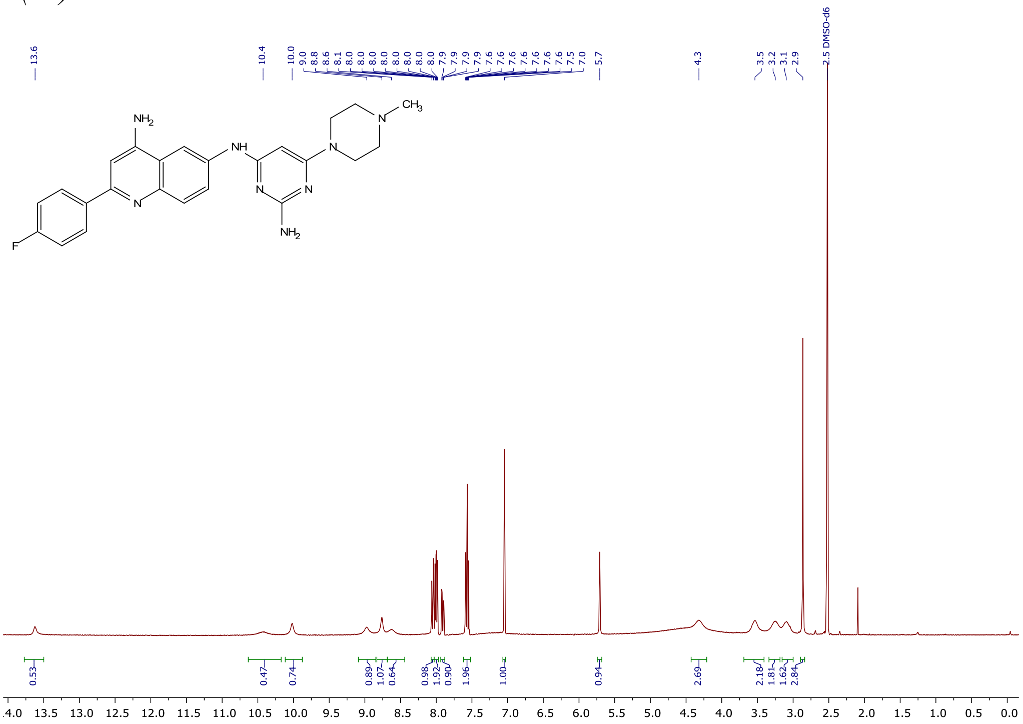

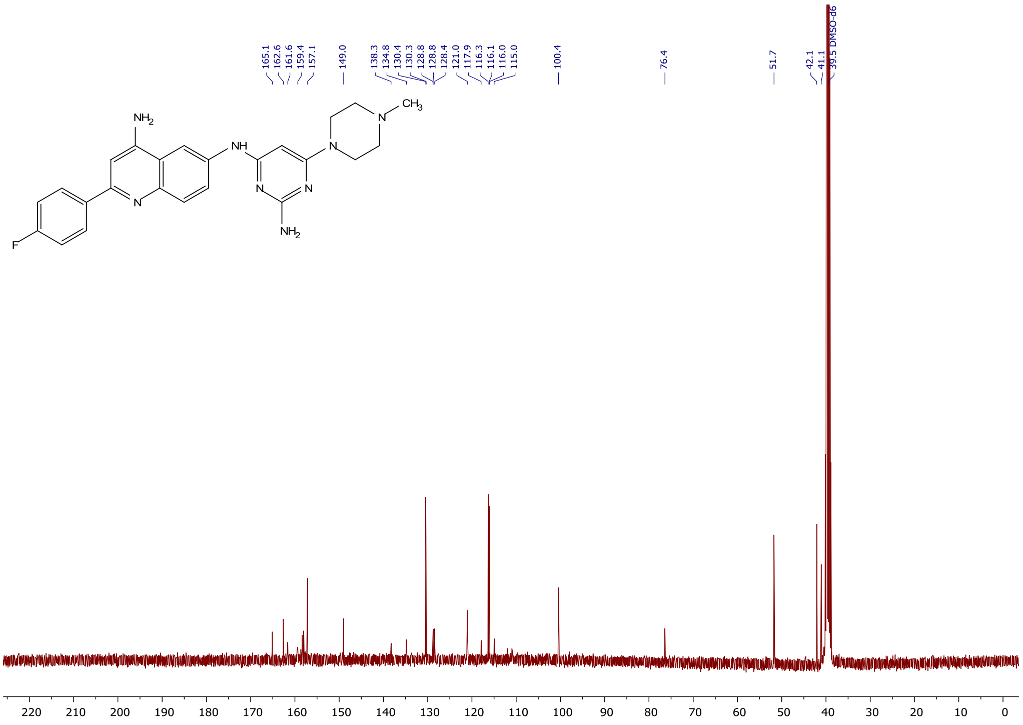

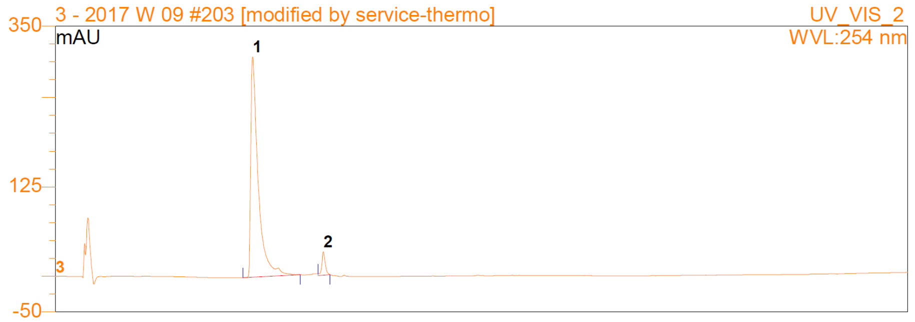

N*6-(2-Amino-6-(4-isopropylpiperazin-1-yl)pyrimidin-4-yl)-2-(4-fluorophenyl)quinoline-4,6-diamine* (***8d***).

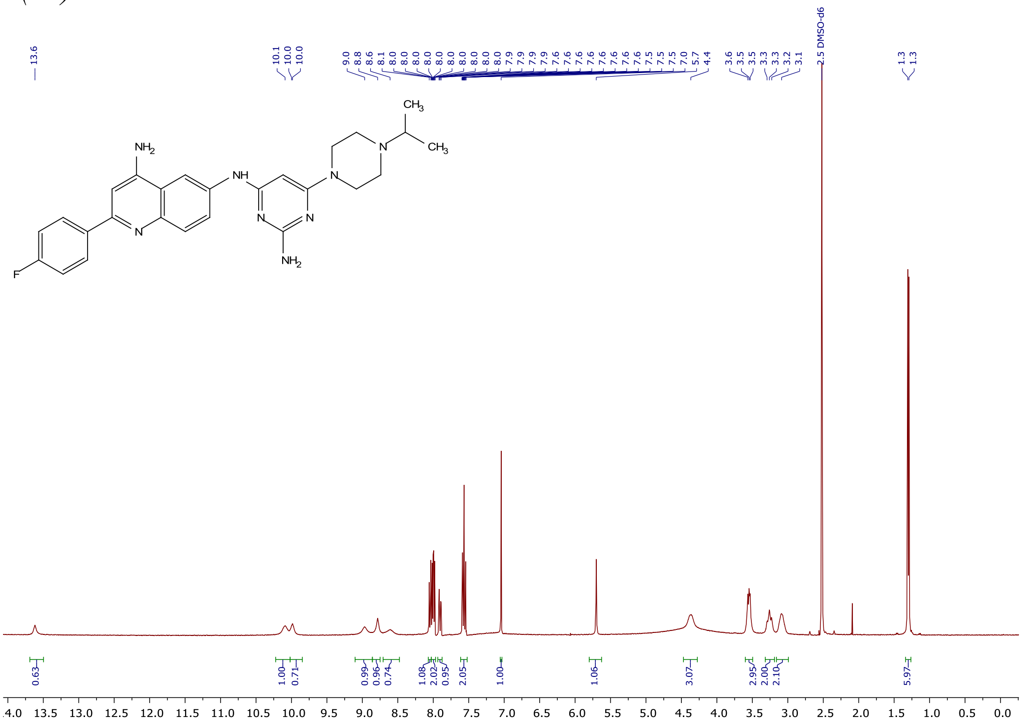

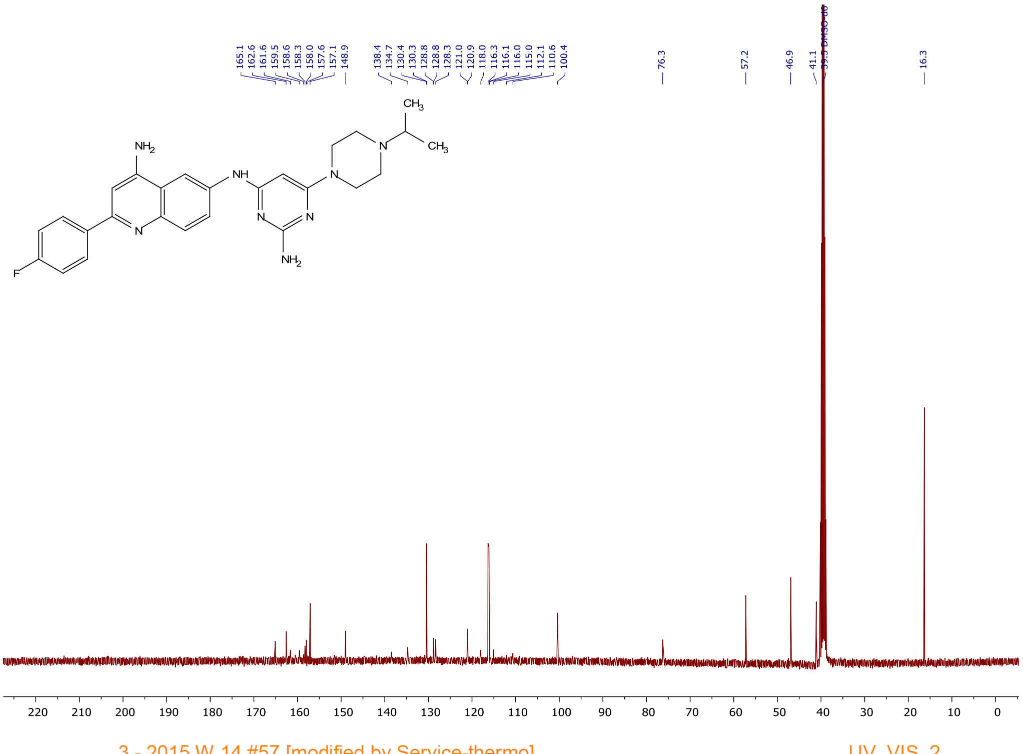

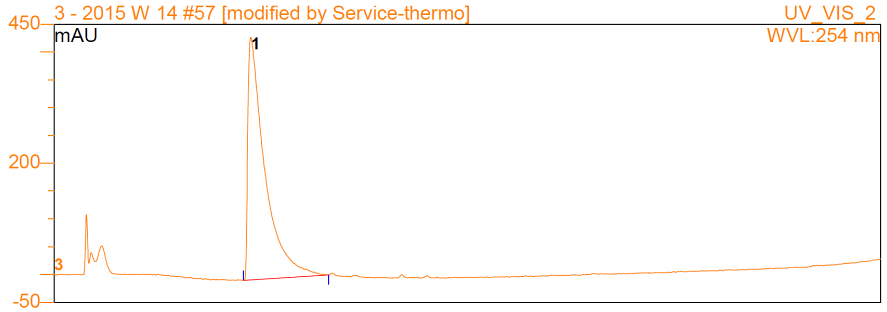

*2-(4-(2-Amino-6-((4-amino-2-(4-fluorophenyl)quinolin-6-yl)amino)pyrimidin-4-yl)piperazin-1-yl)ethan-1-ol (**8e**).*

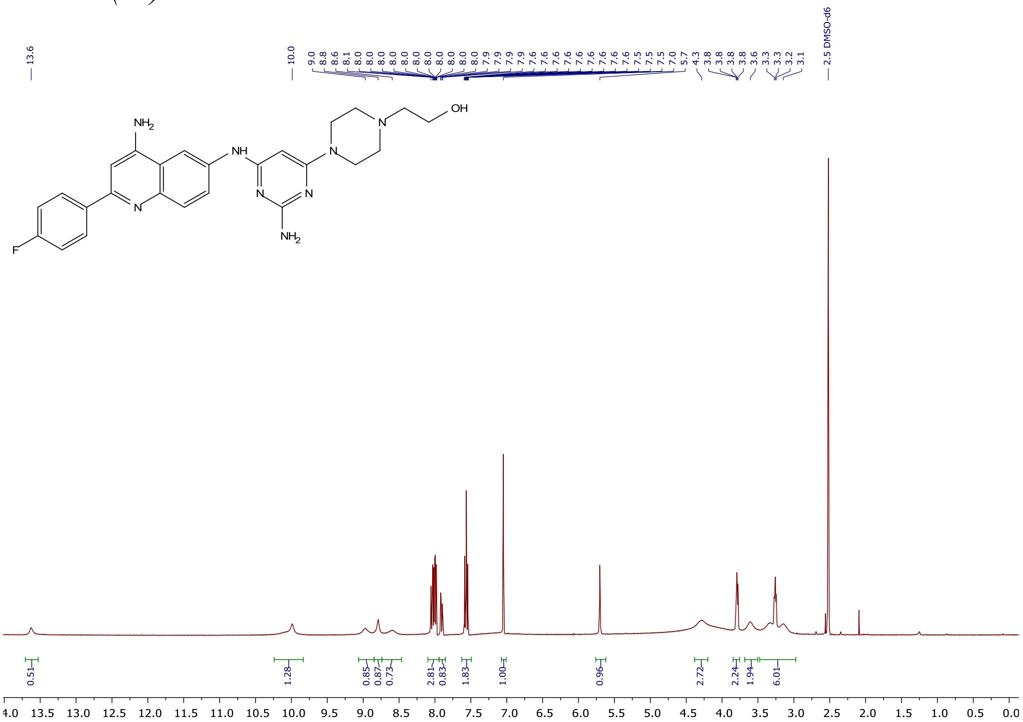

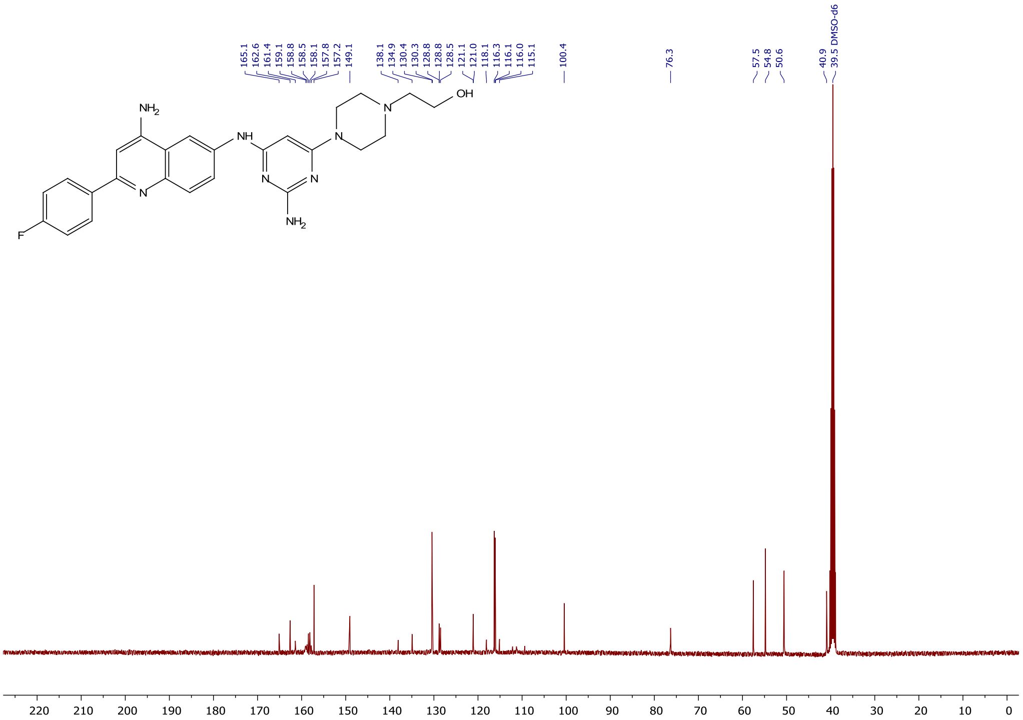

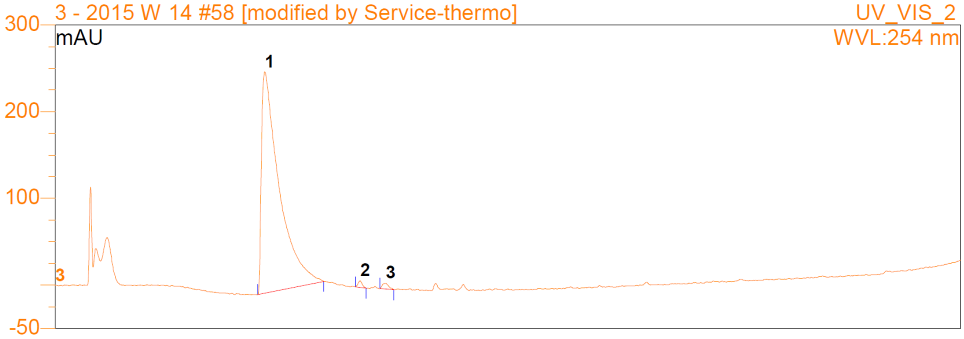

*N6-(2-Amino-6-(4-(dimethylamino)piperidin-1-yl)pyrimidin-4-yl)-2-(4-fluorophenyl)quinoline-4,6-diamine (**8f**).*

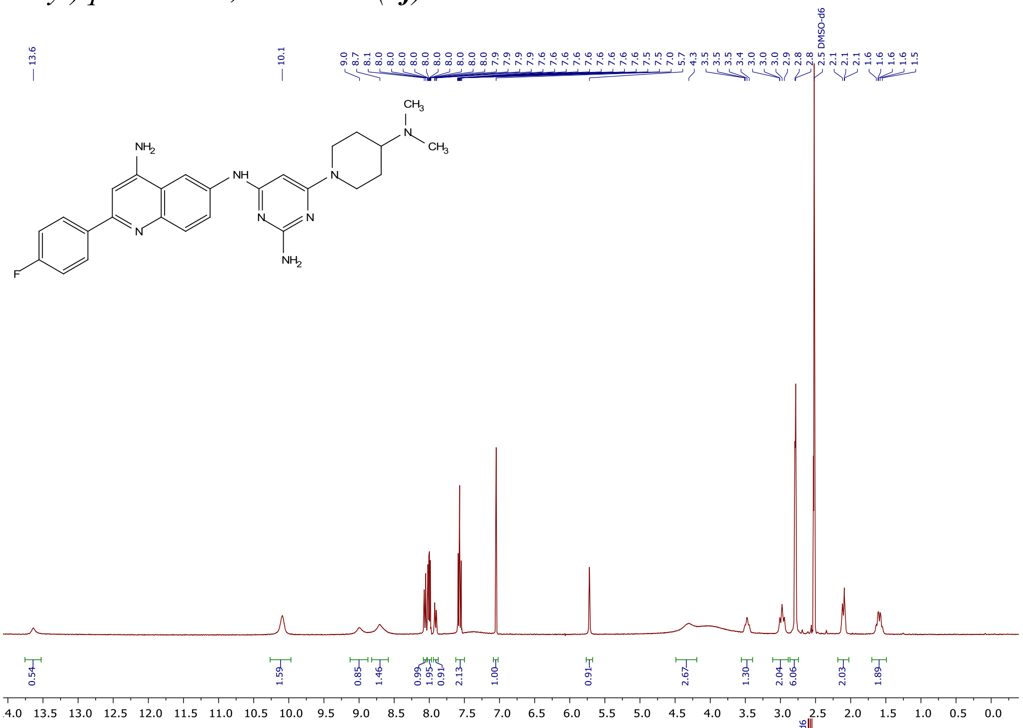

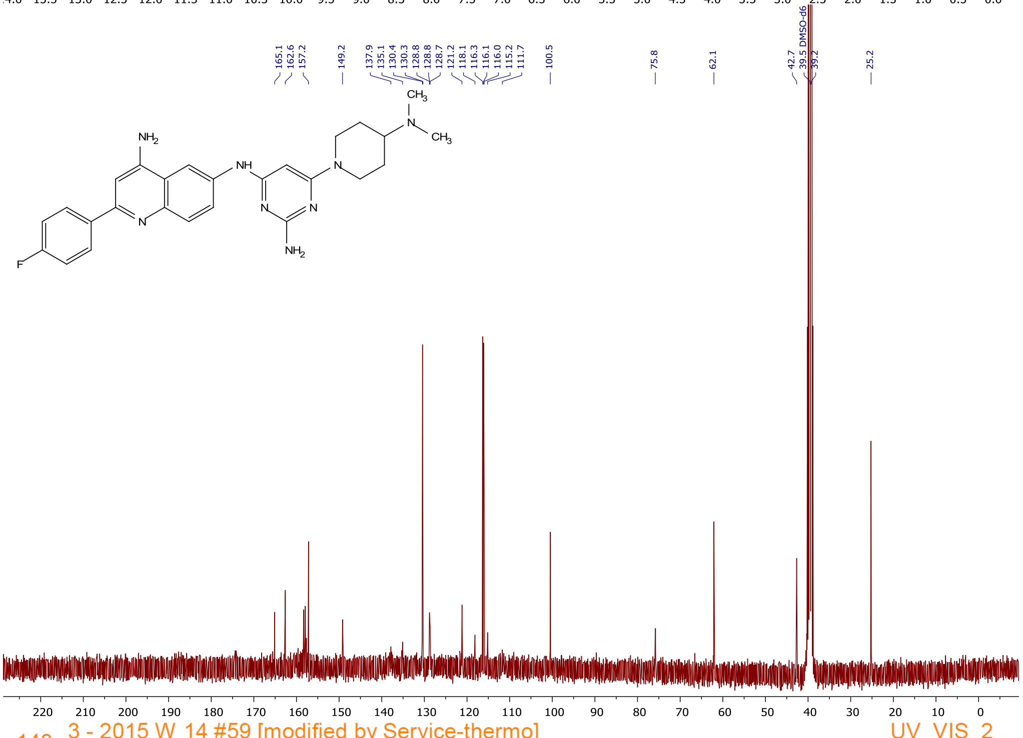

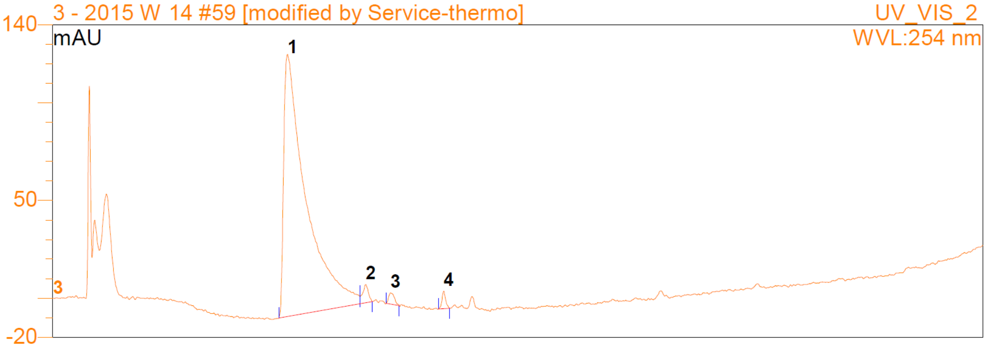

tert-*Butyl ((1-(2-Amino-6-((4-amino-2-(4-fluorophenyl)quinolin-6-yl)amino)pyrimidin-4-yl)piperidin-4-yl)methyl)carbamate (**8g**).*

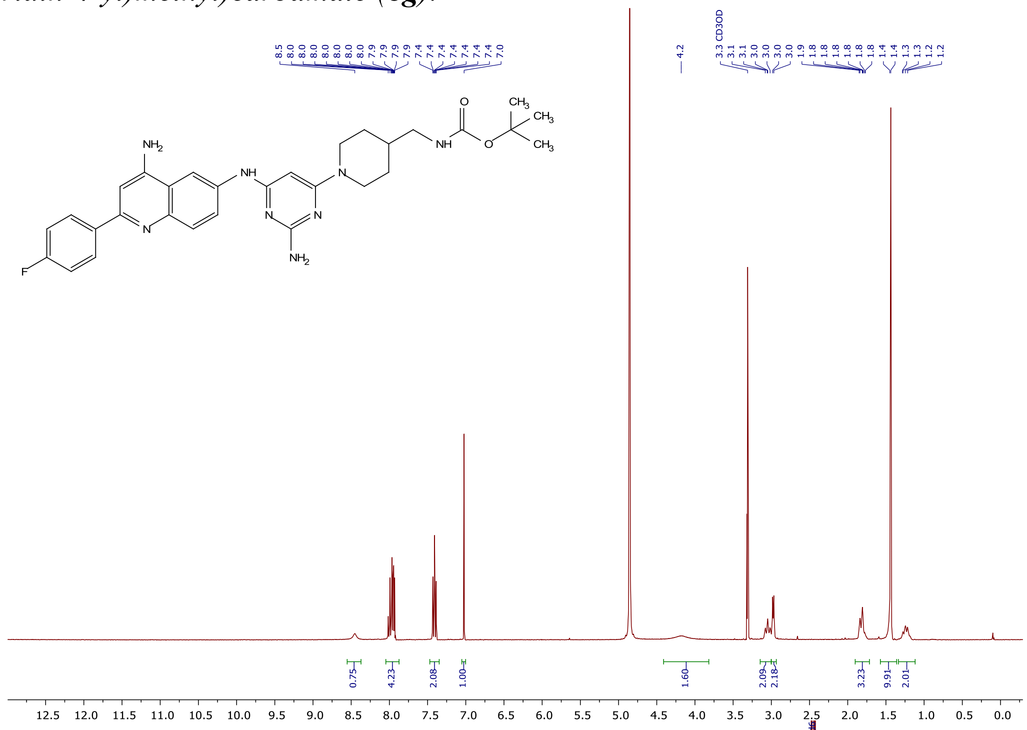

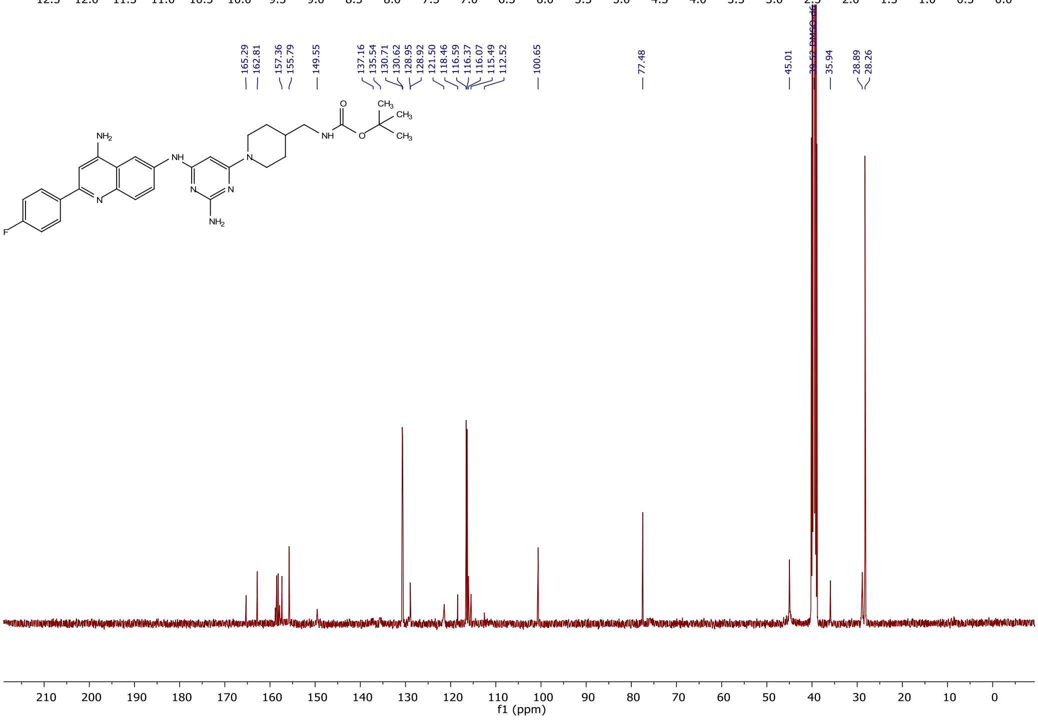

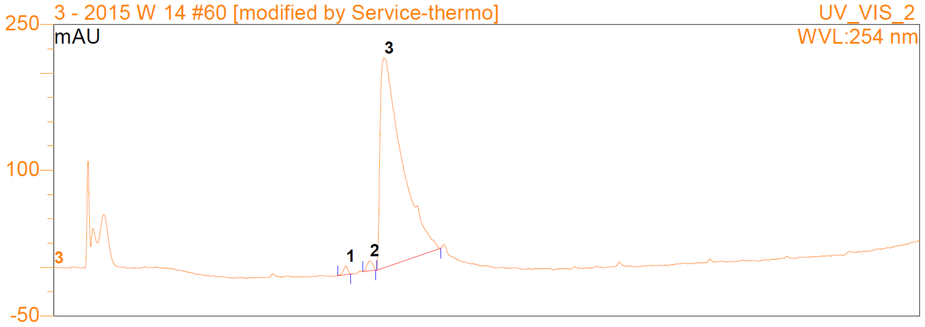

HSQC

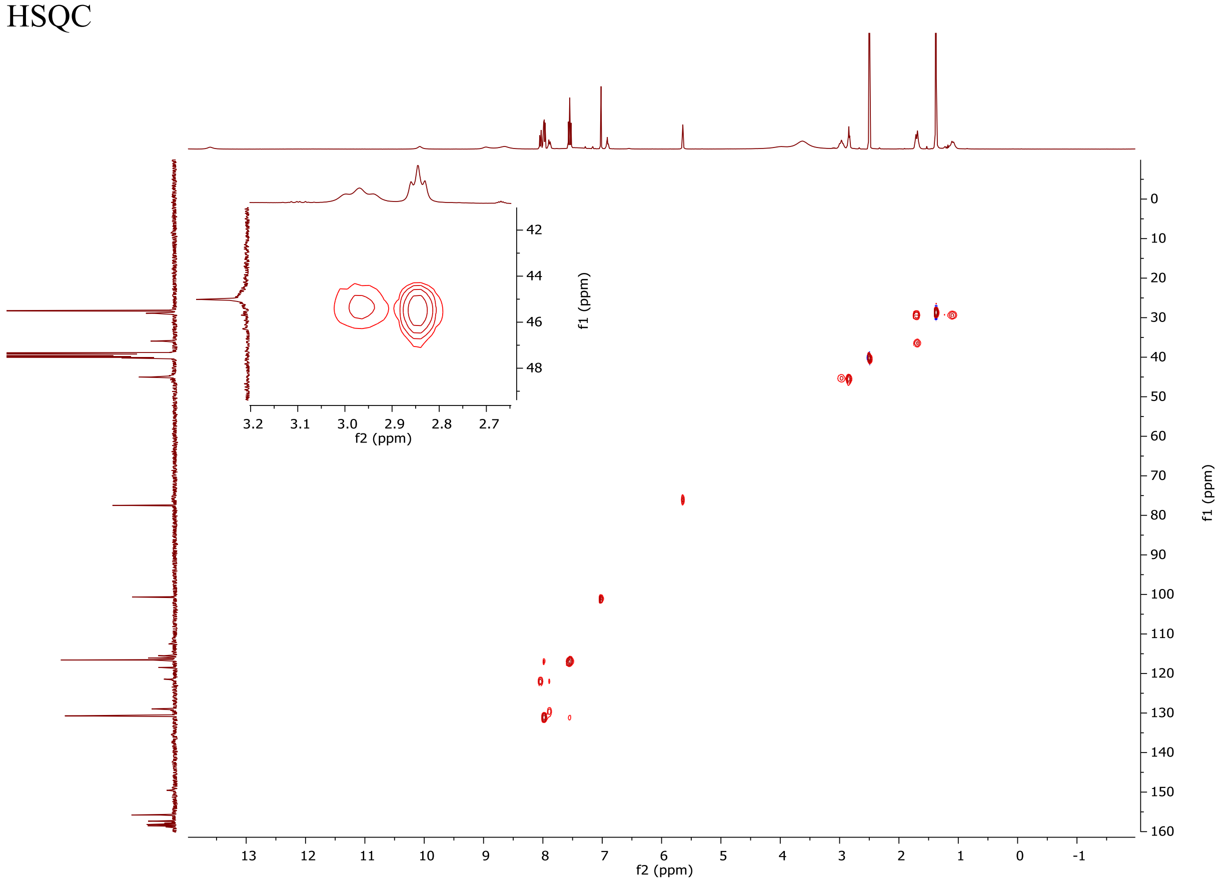

HMBC

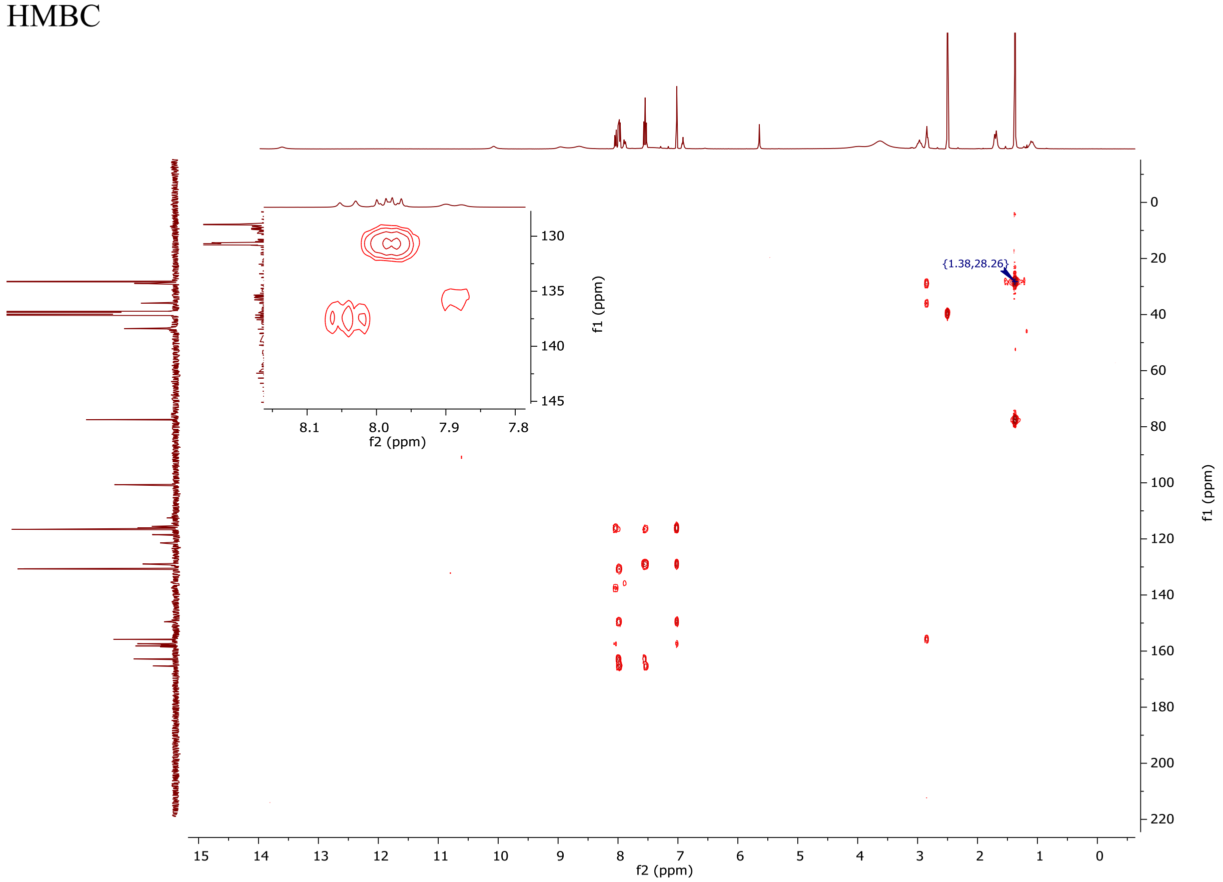

N*6-(2-Amino-6-(4-(aminomethyl)piperidin-1-yl)pyrimidin-4-yl)-2-(4-fluorophenyl)quinoline-4,6-diamine (**8h**).*

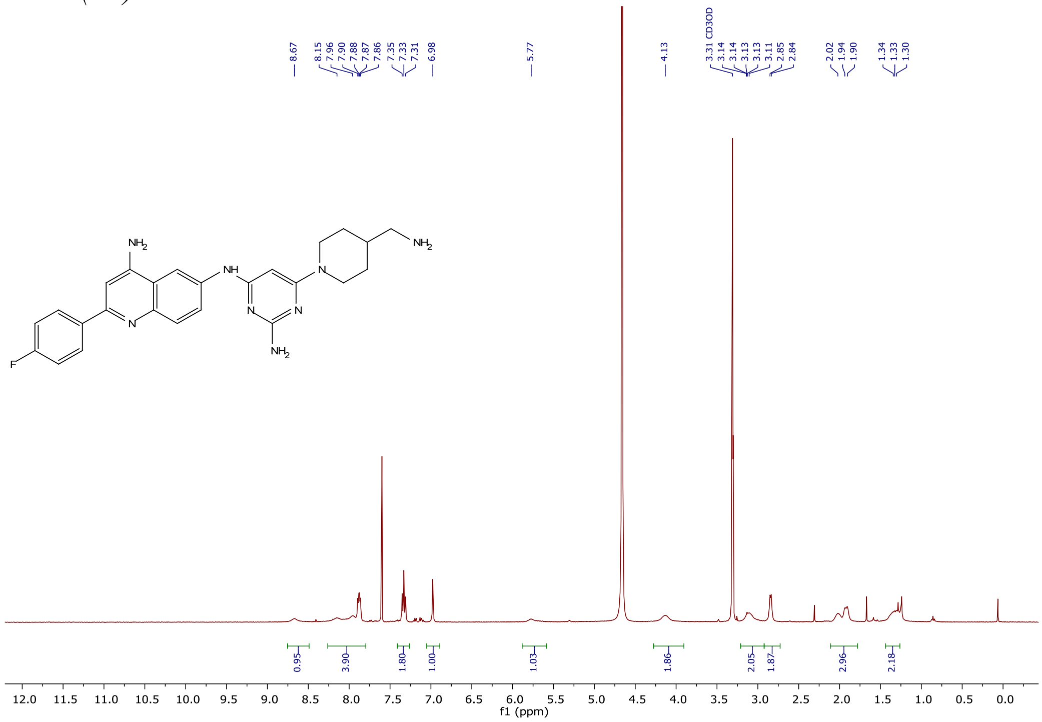

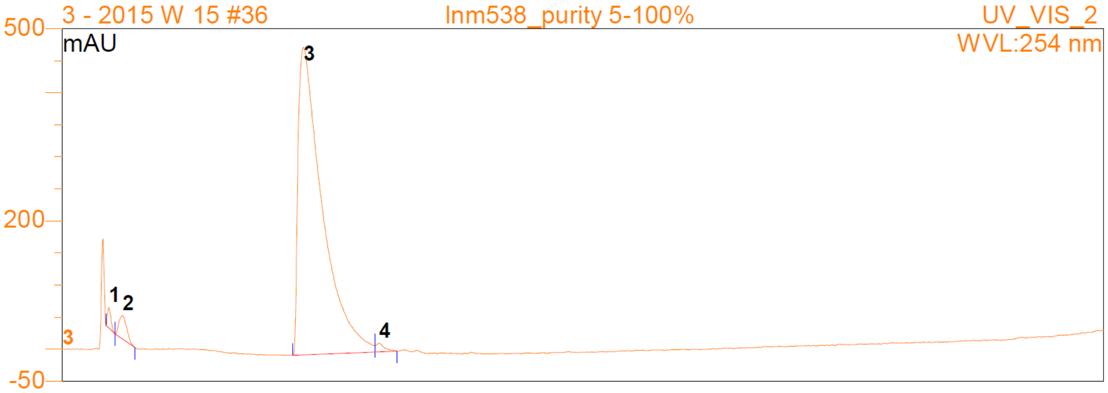

tert-*Butyl (1-(2-Amino-6-((4-amino-2-(4-fluorophenyl)quinolin-6-yl)amino)pyrimidin-4-yl)piperidin-4-yl)carbamate (**8i**).*

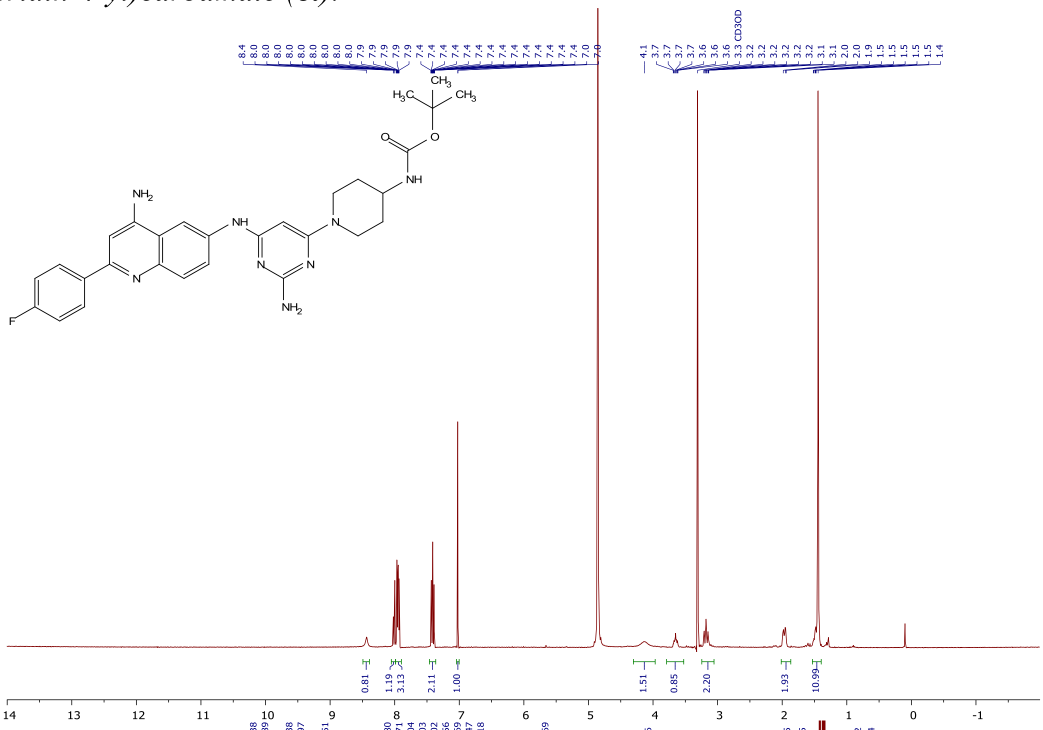

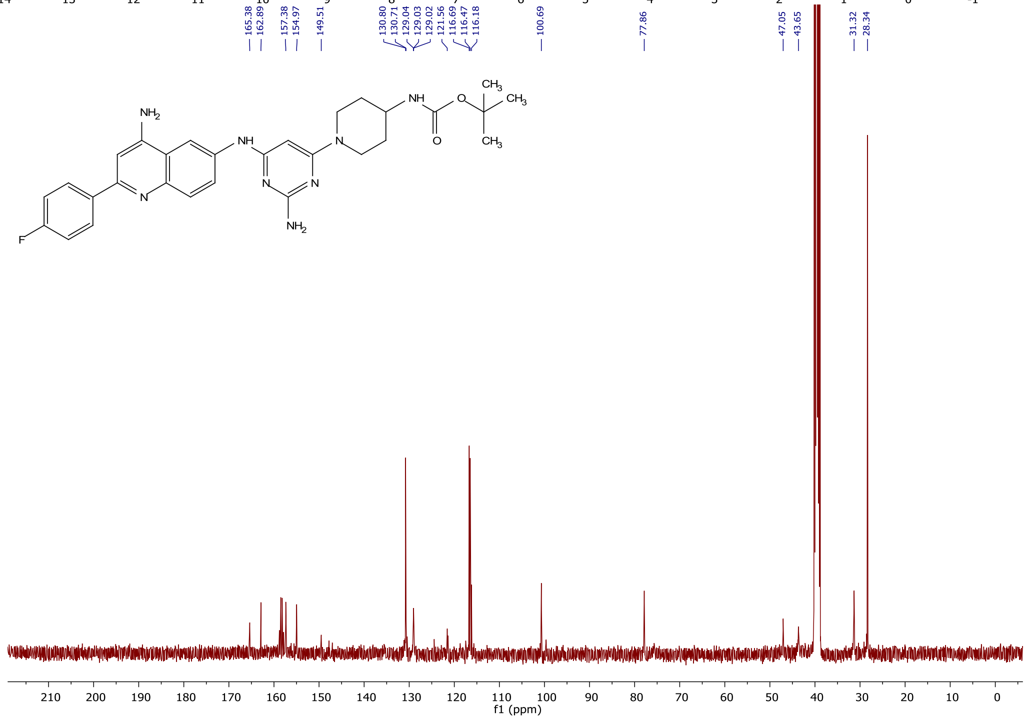

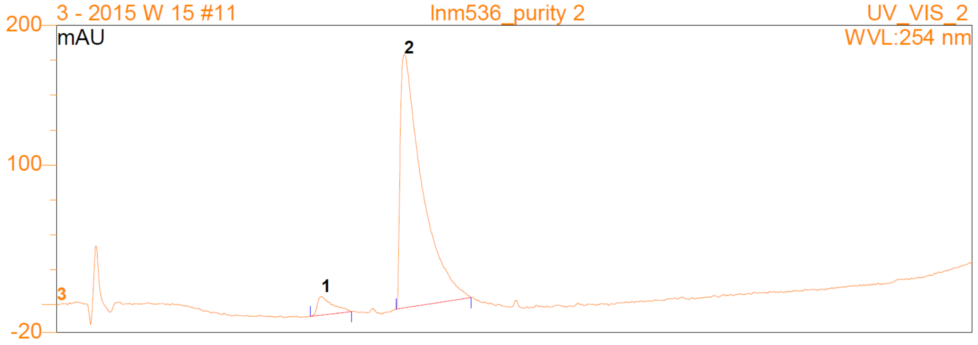

N*6-(2-Amino-6-(4-aminopiperidin-1-yl)pyrimidin-4-yl)-2-(4-fluorophenyl)quinoline-4,6-diamine (**8j**).*

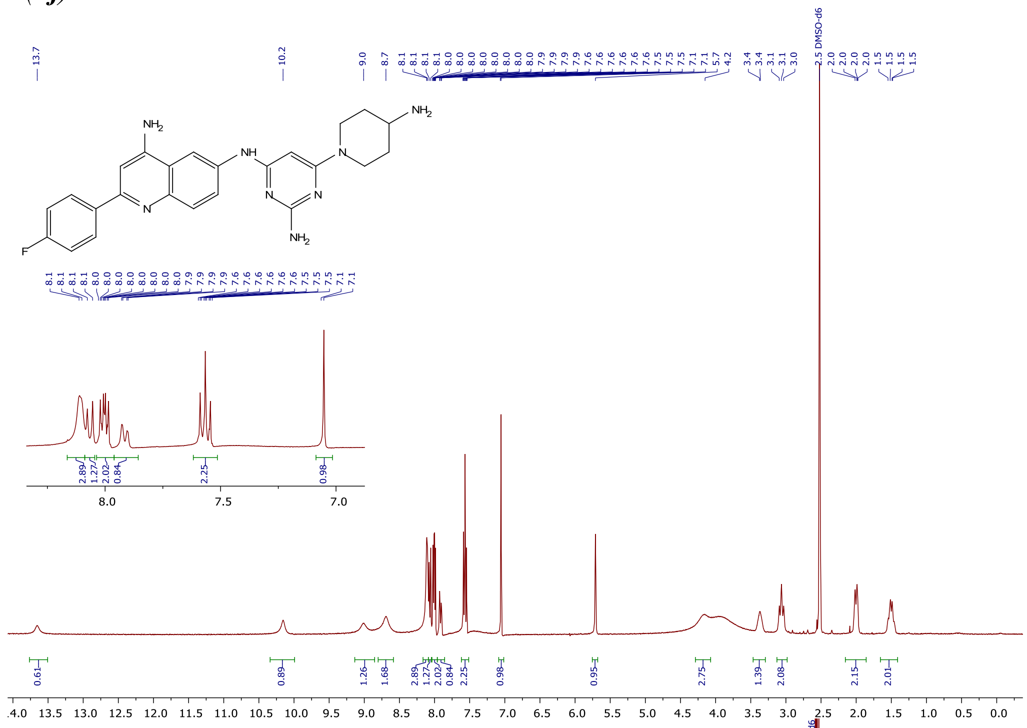

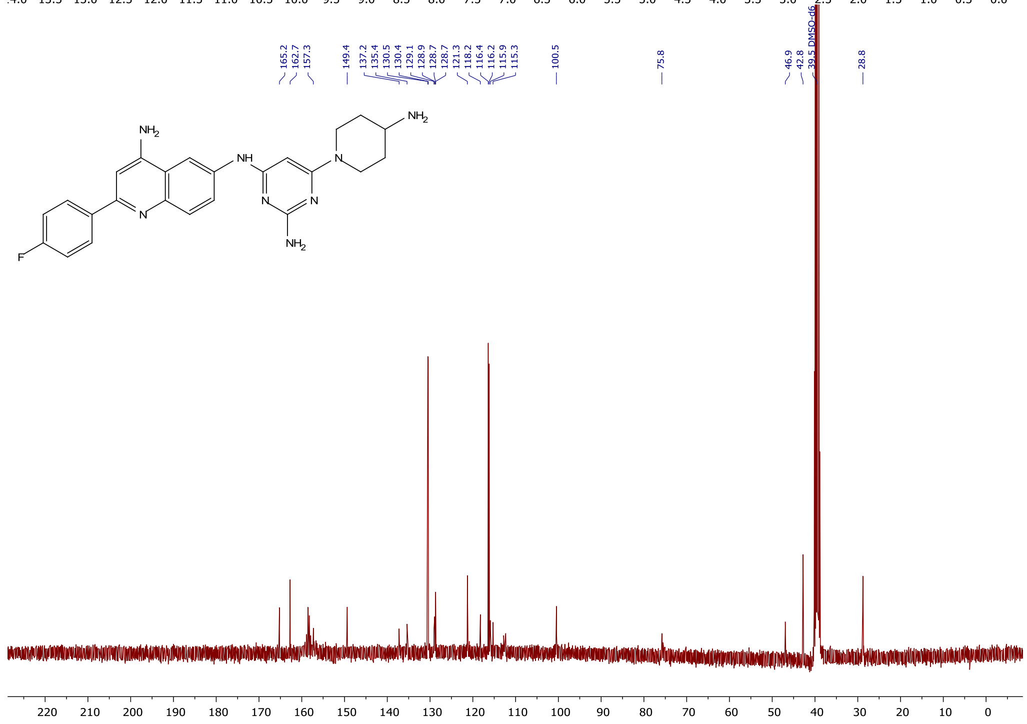

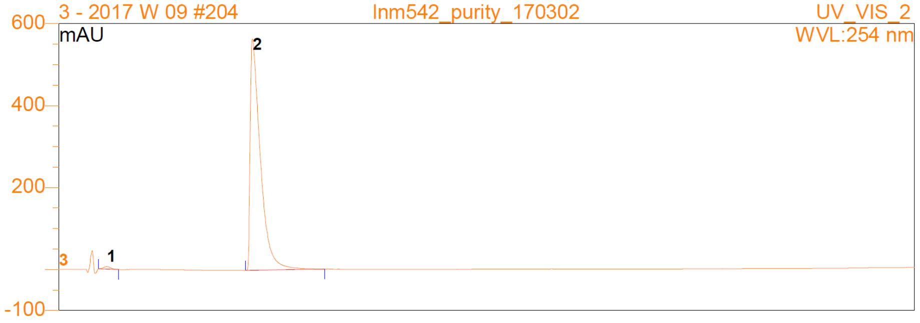

N*4-(4-Amino-2-(4-fluorophenyl)quinolin-6-yl)-*N*6,*N*6-dimethylpyrimidine-2,4,6-triamine* ***(8k)***.

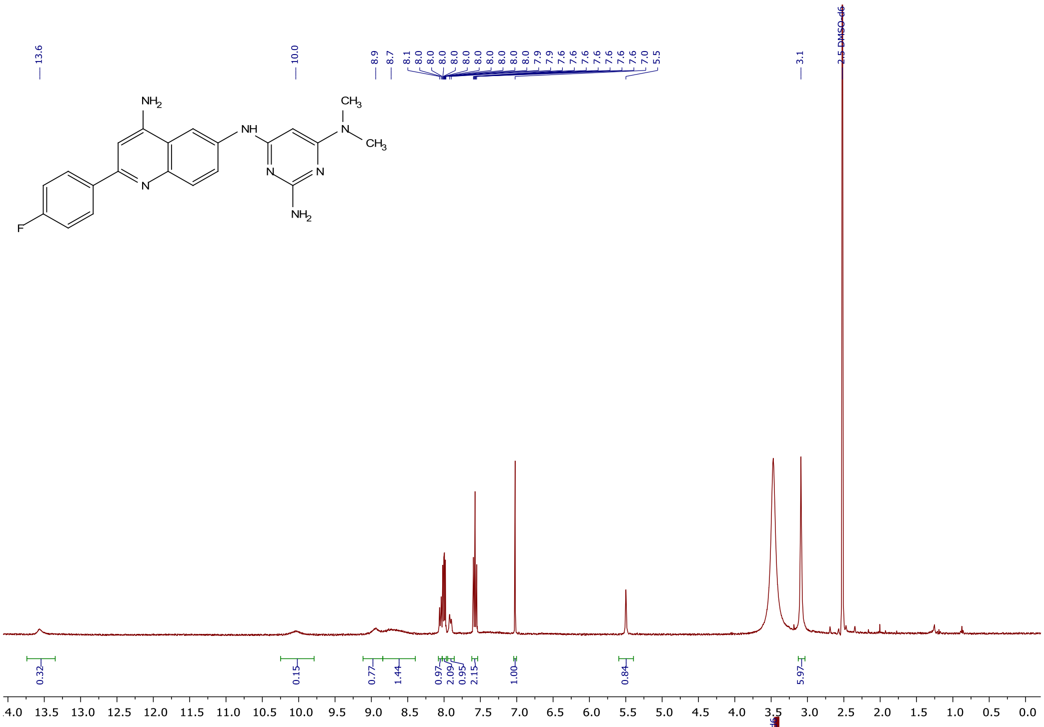

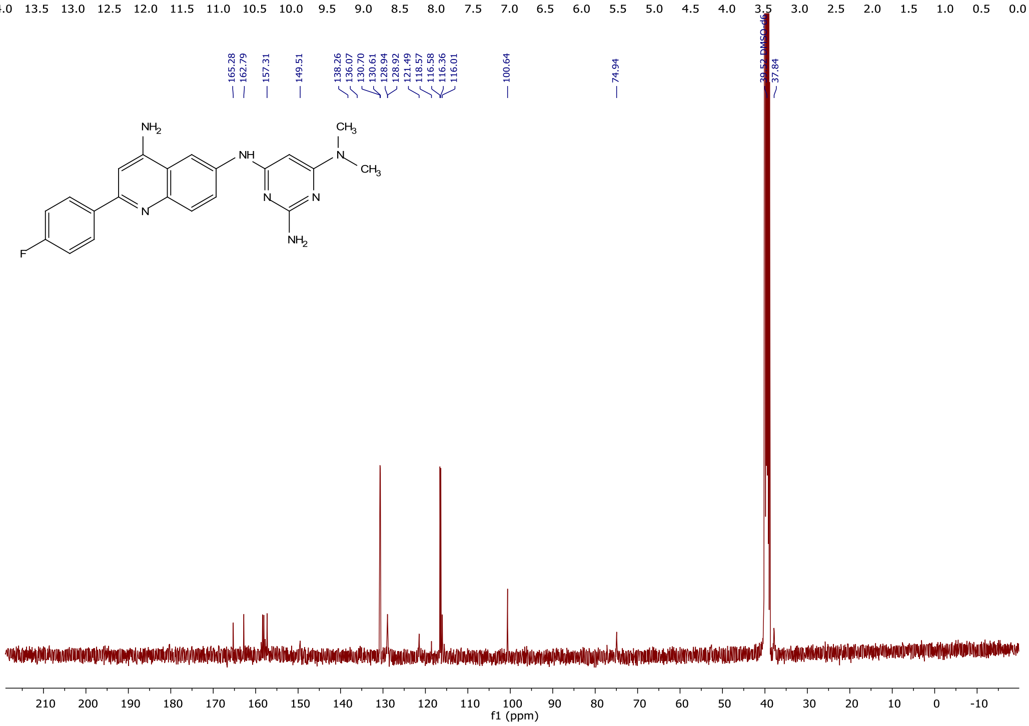

HMBC

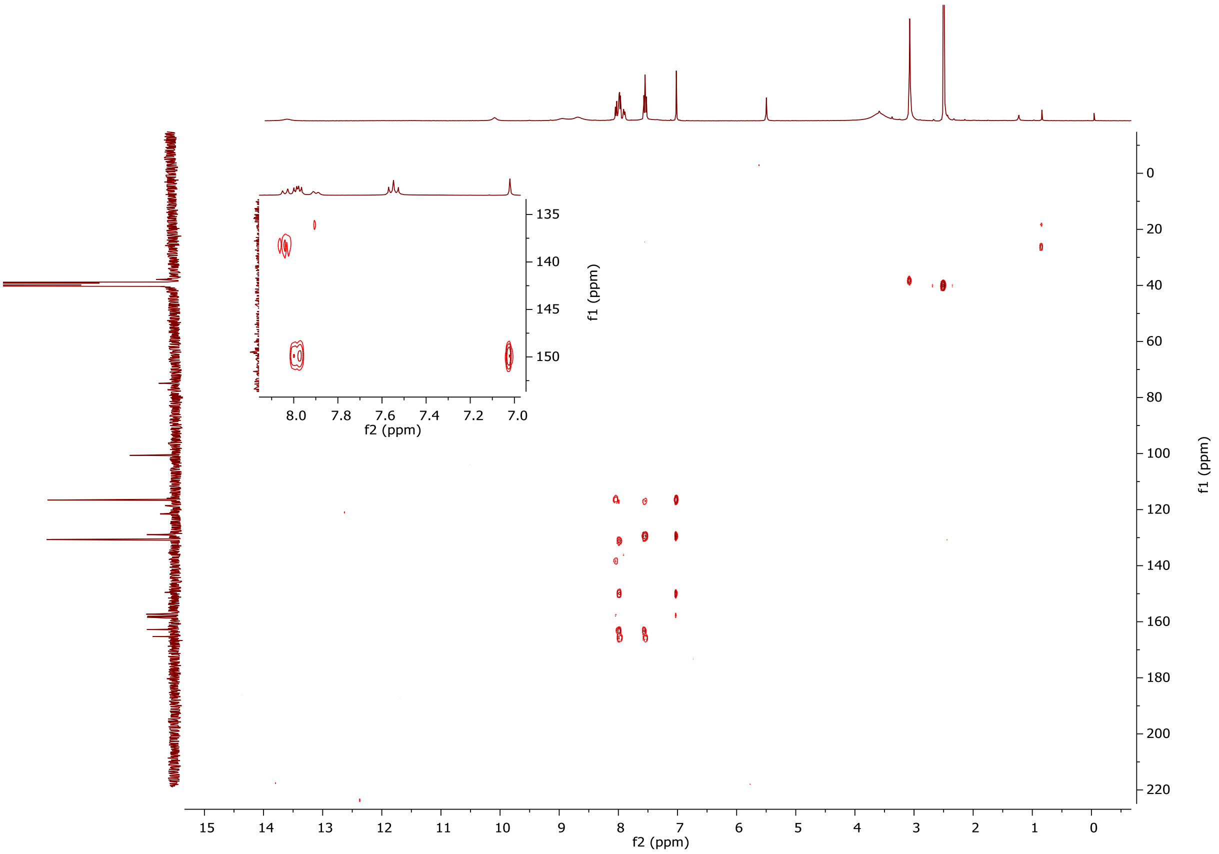

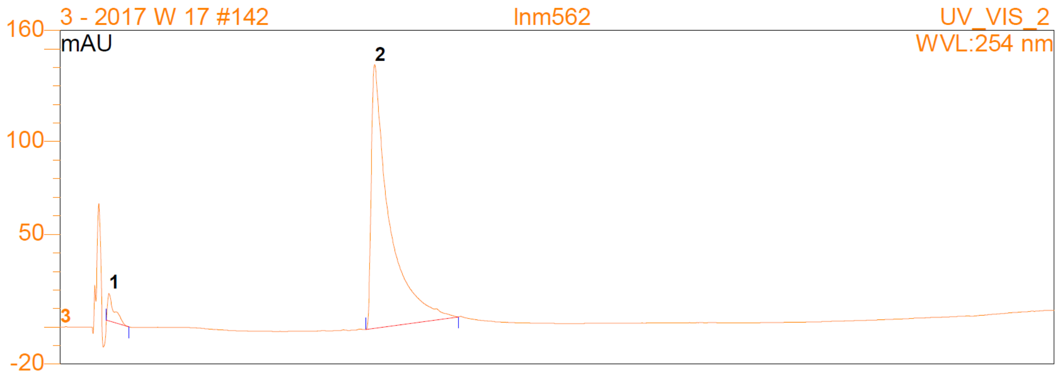

*2-((2-Amino-6-((4-amino-2-(4-fluorophenyl)quinolin-6-yl)amino)pyrimidin-4-yl)(methyl)amino)ethan-1-ol (**8l**).*

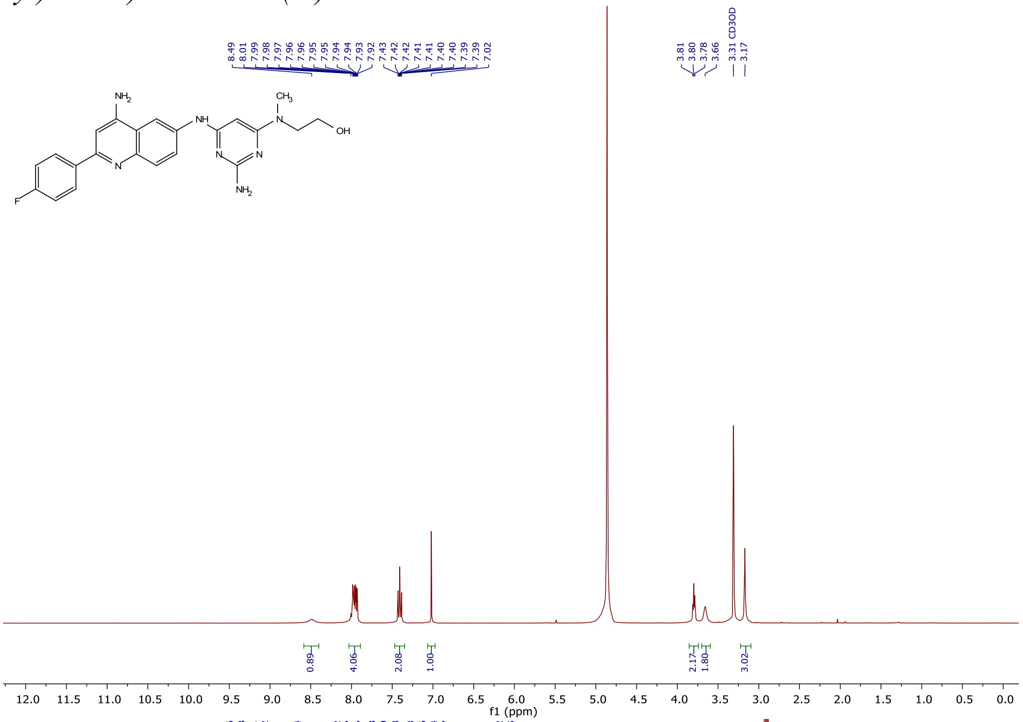

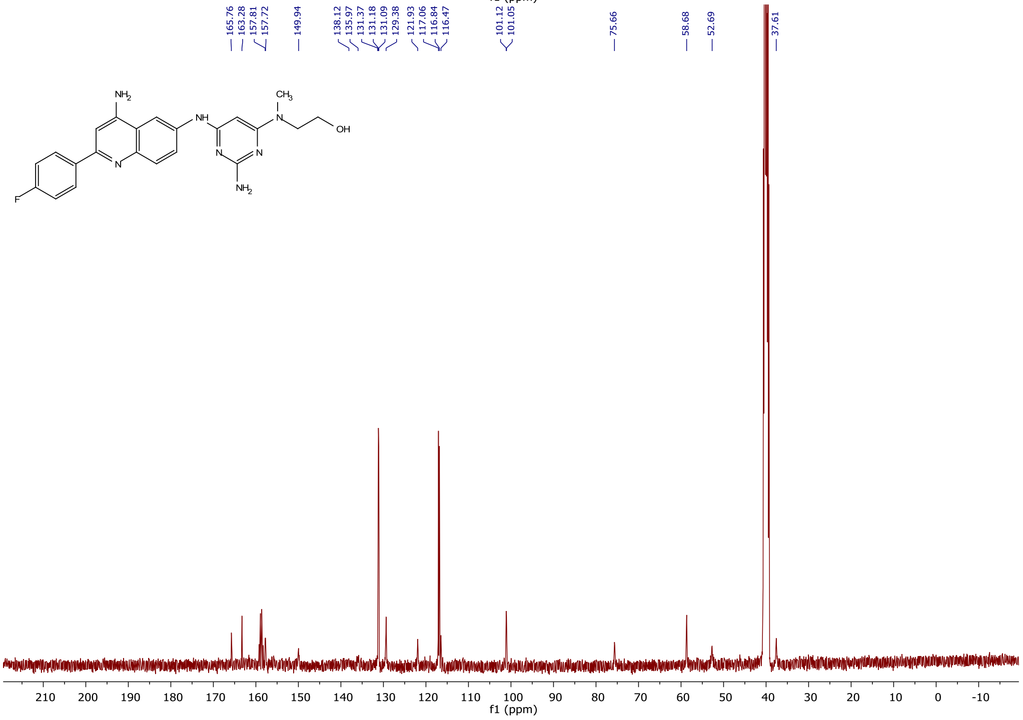

HMBC

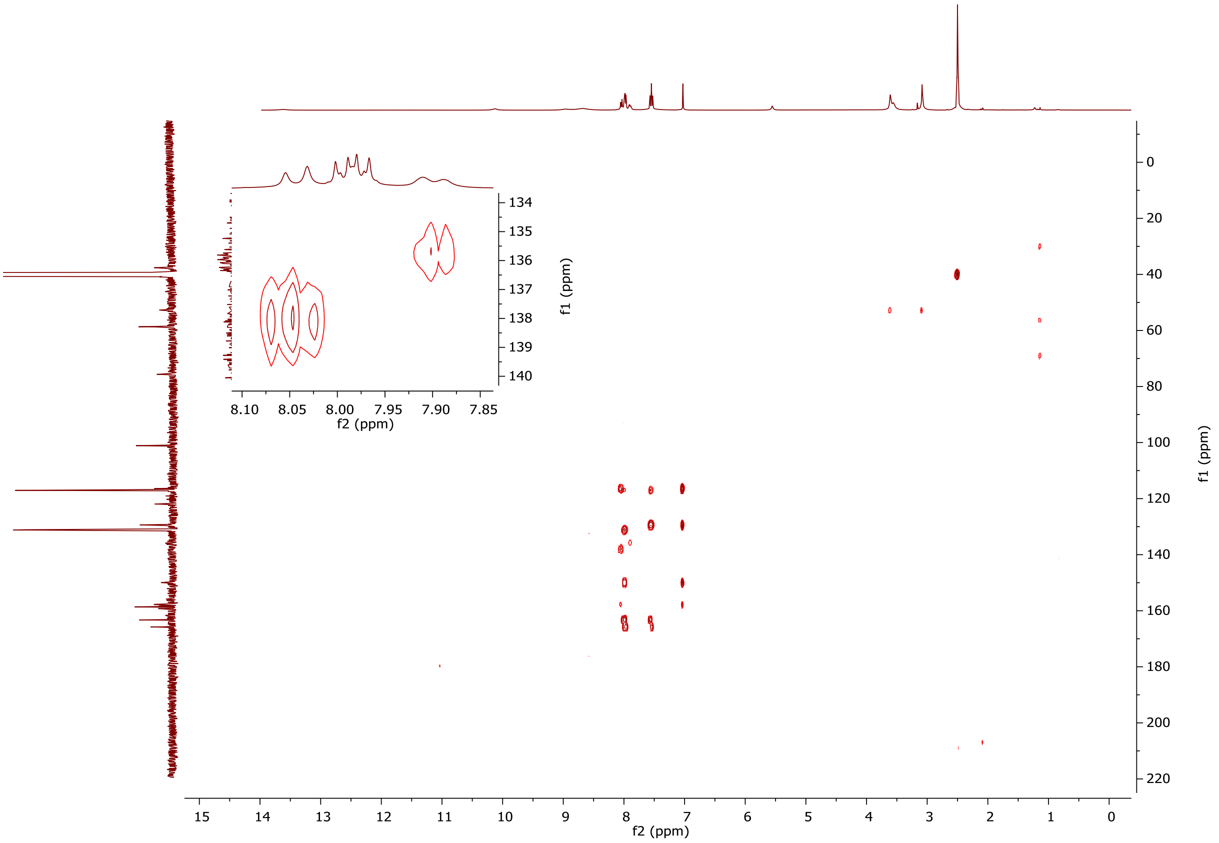

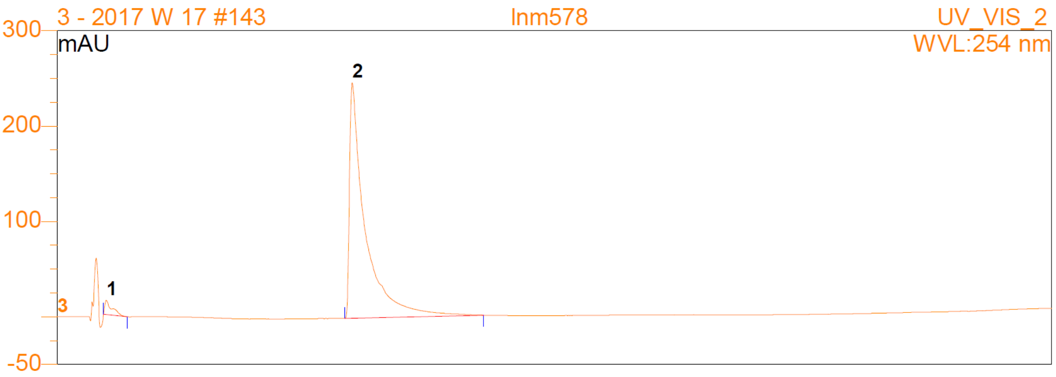

*N4-(4-amino-2-(4-fluorophenyl) quinolin-6-yl)-N6-methyl-N6-(2-(methylamino) ethyl) pyrimidine-2,4,6-triamine (**8m**).*

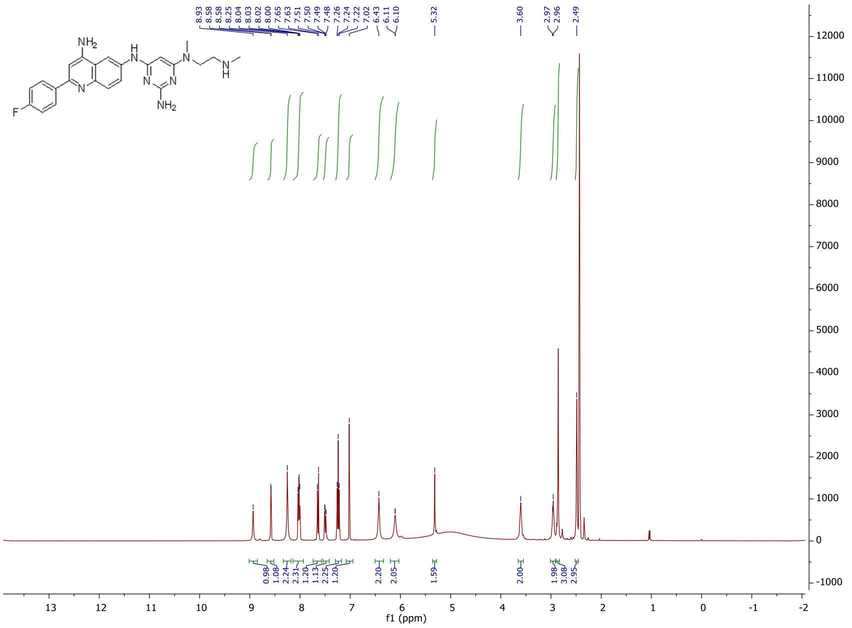

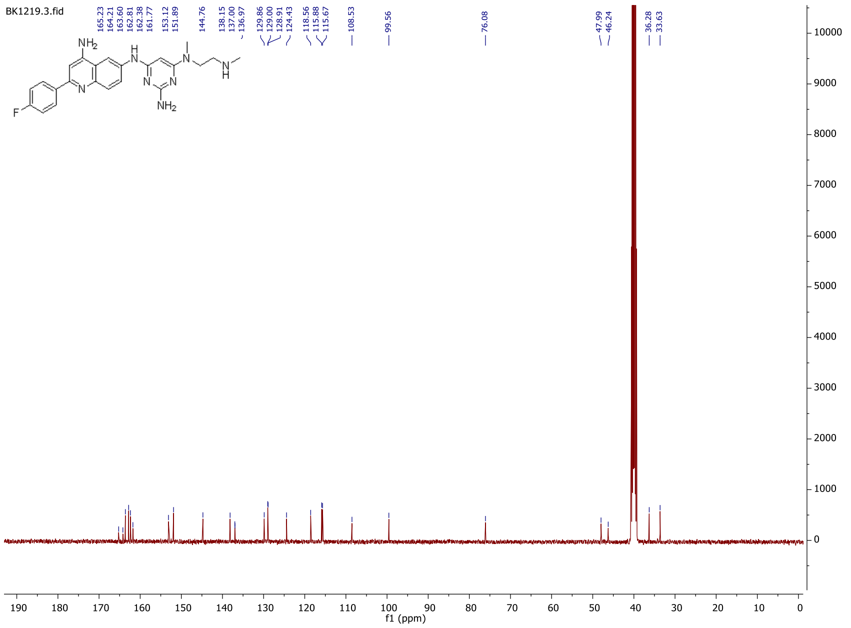

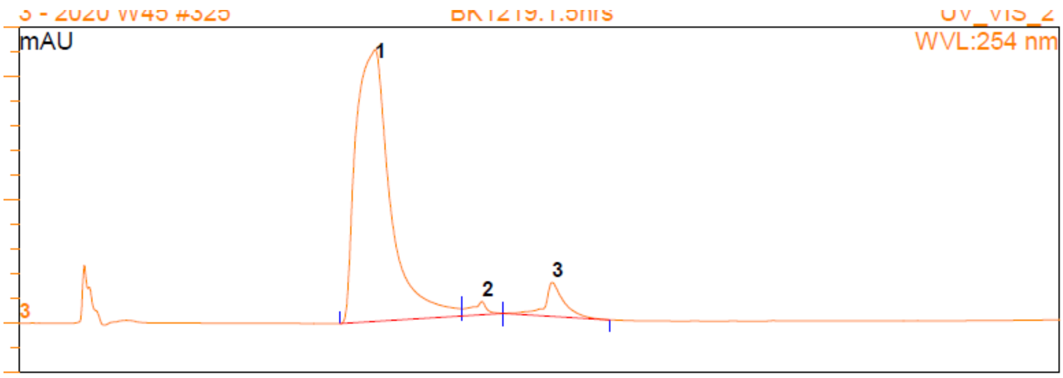

*(1-(2-amino-6-((4-amino-2-(4-fluorophenyl)quinolin-6-yl)amino)pyrimidin-4-yl)piperidin-4-yl)methanol (**8n**).*

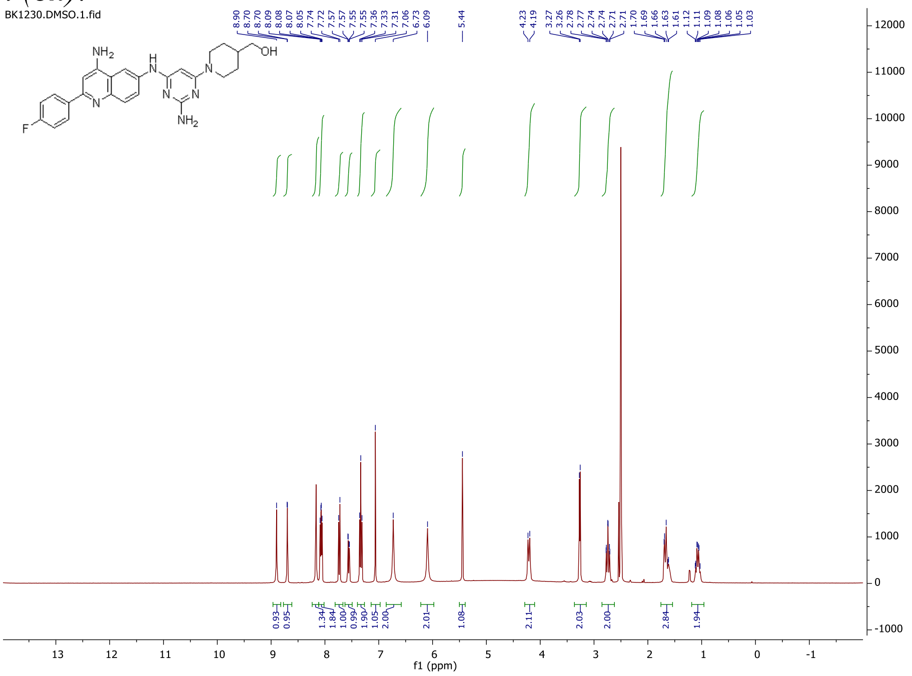

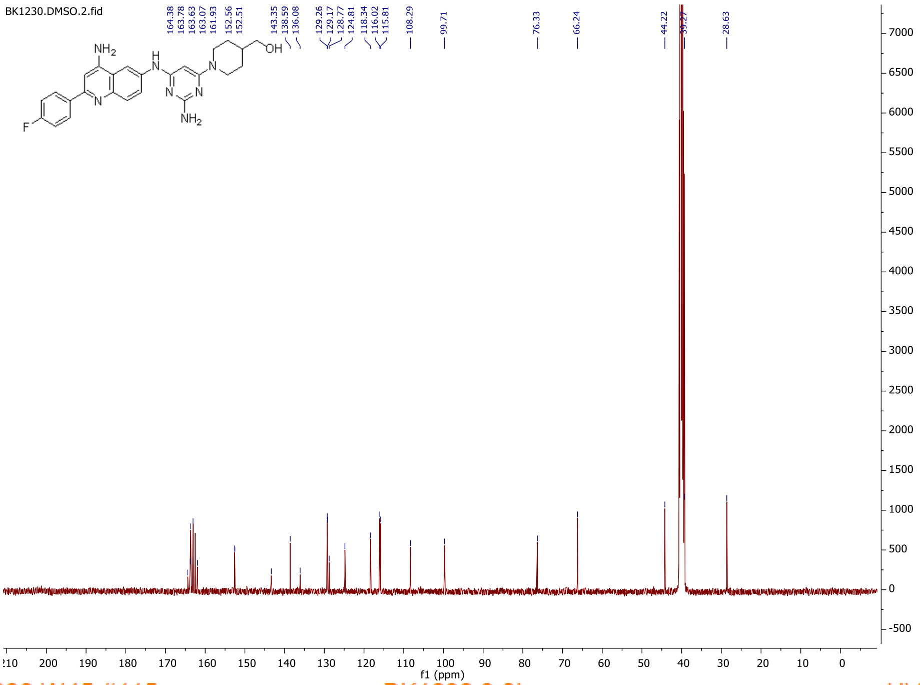

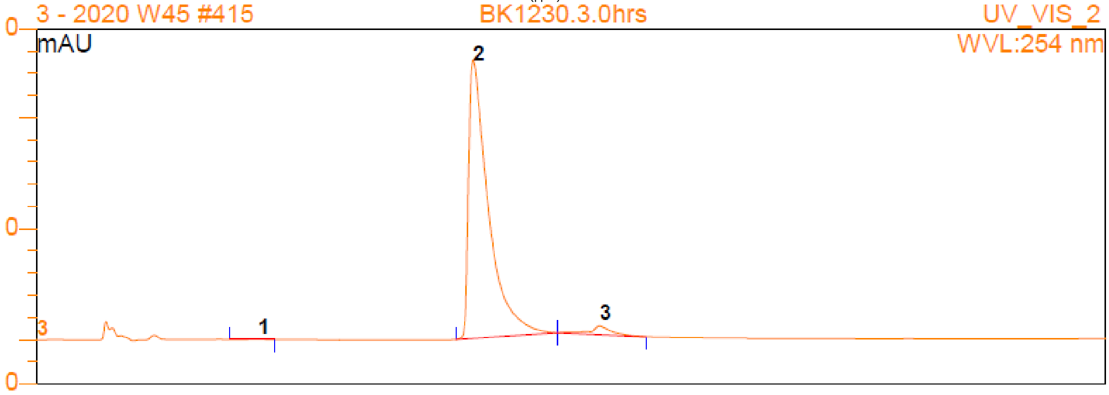

1-(2-amino-6-((4-amino-2-(4-fluorophenyl)quinolin-6-yl)amino)pyrimidin-4-yl)piperidin-3-ol ***(8o)***.

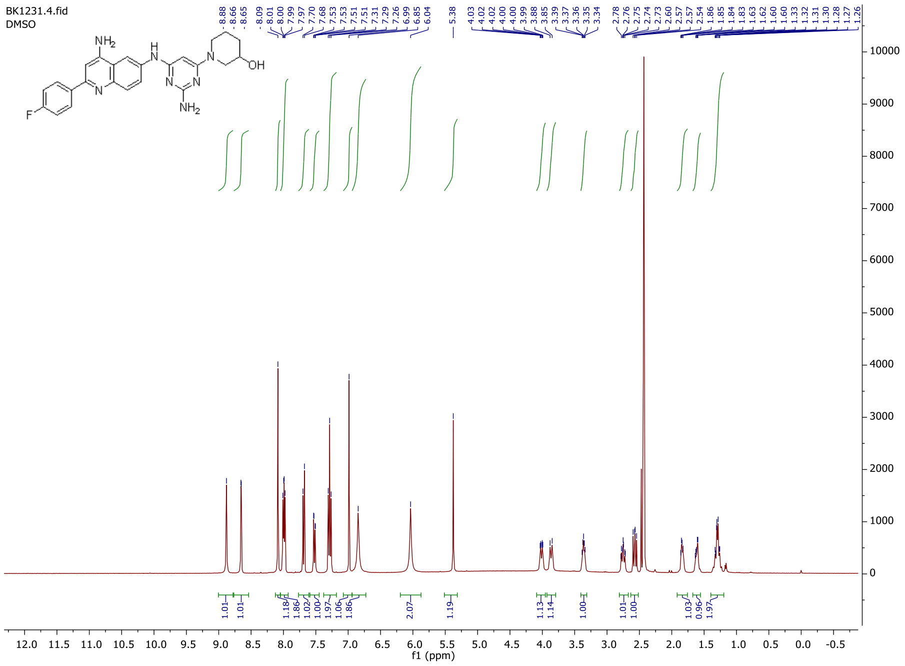

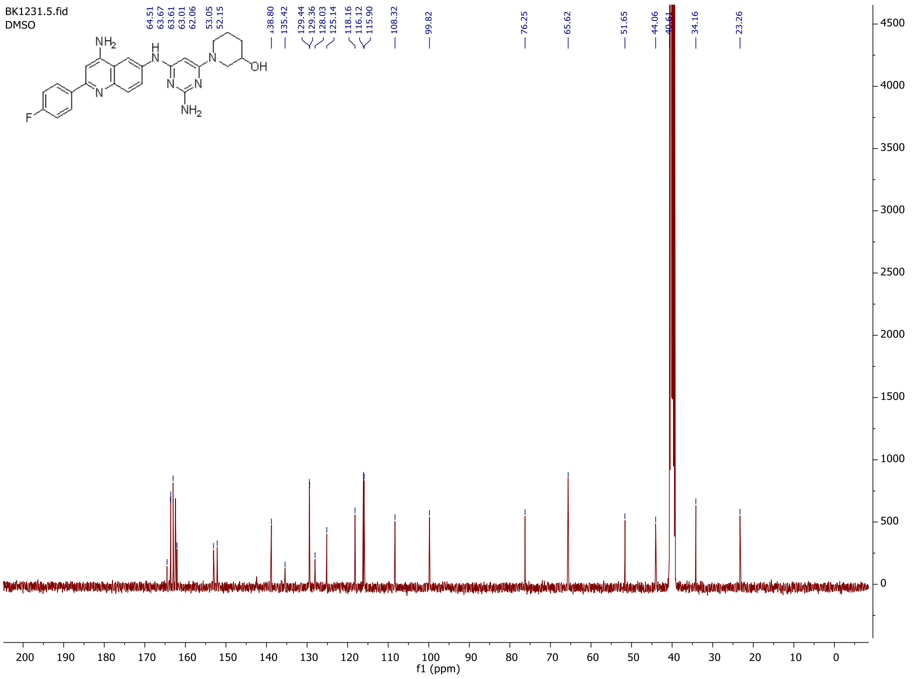

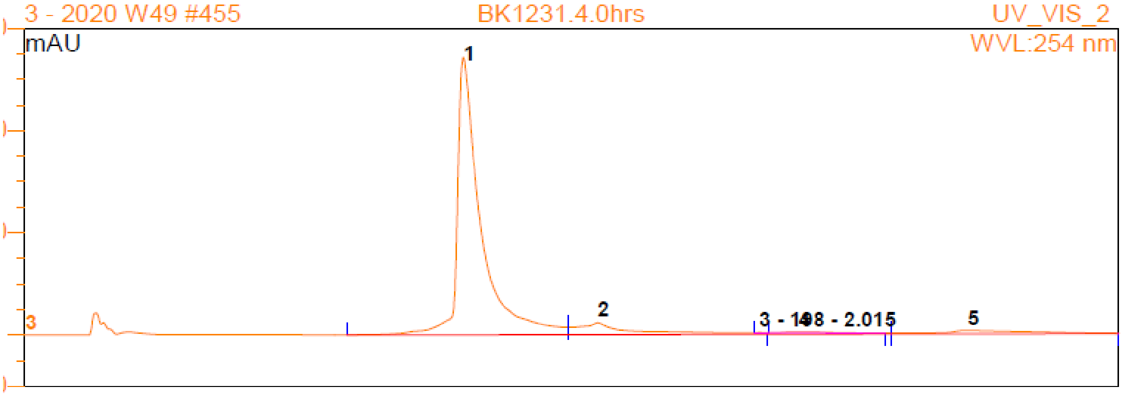

*1-(2-amino-6-((4-amino-2-(4-fluorophenyl)quinolin-6-yl)amino)pyrimidin-4-yl)piperidin-4-ol (**8p**).*

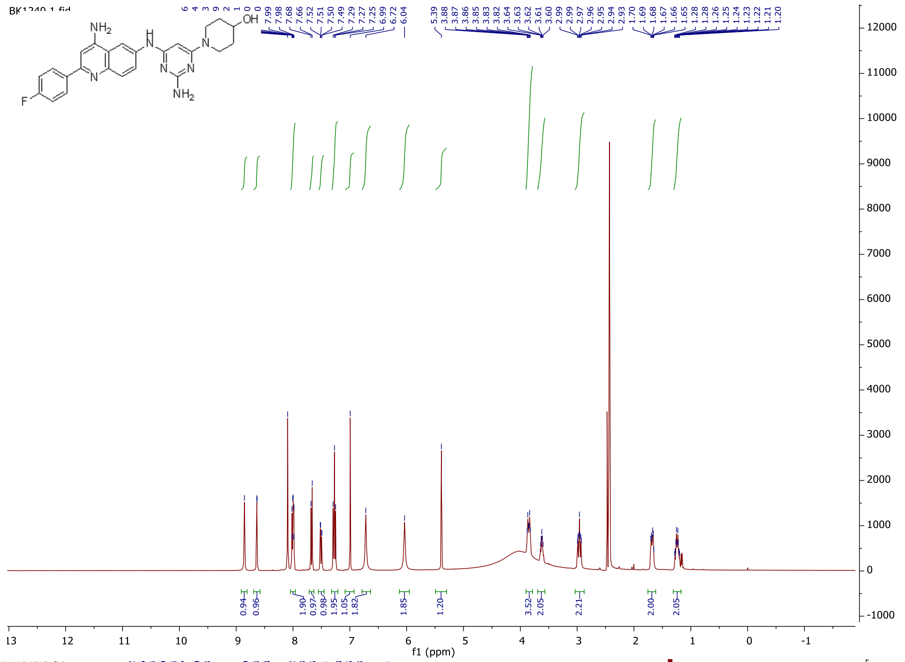

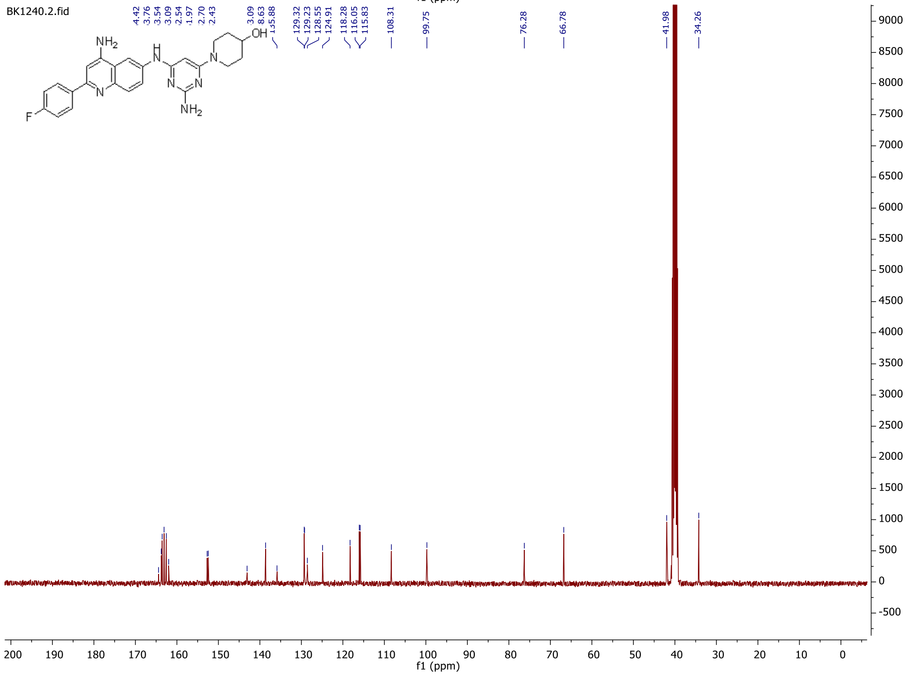

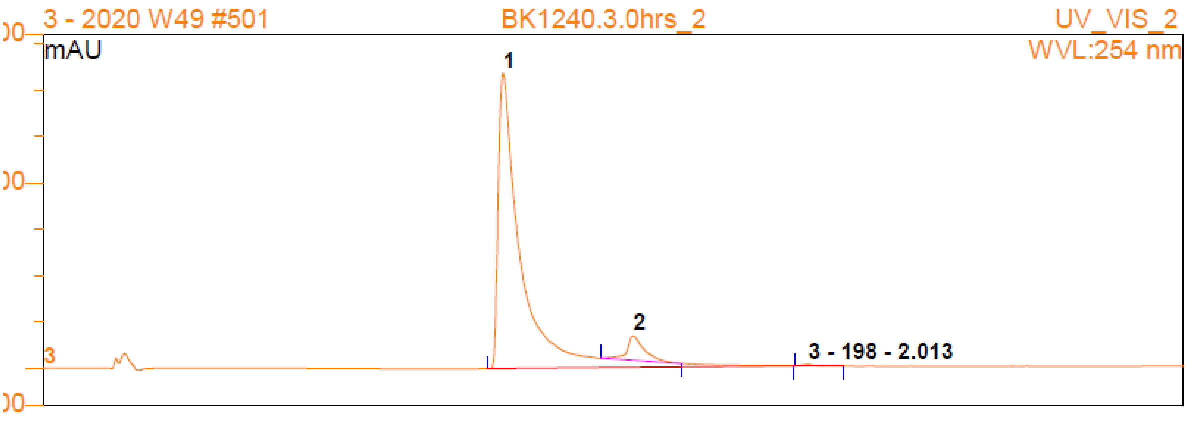

N6-*(2-Amino-6-ethoxypyrimidin-4-yl)-2-(4-fluorophenyl)quinoline-4,6-diamine* (***9a***).

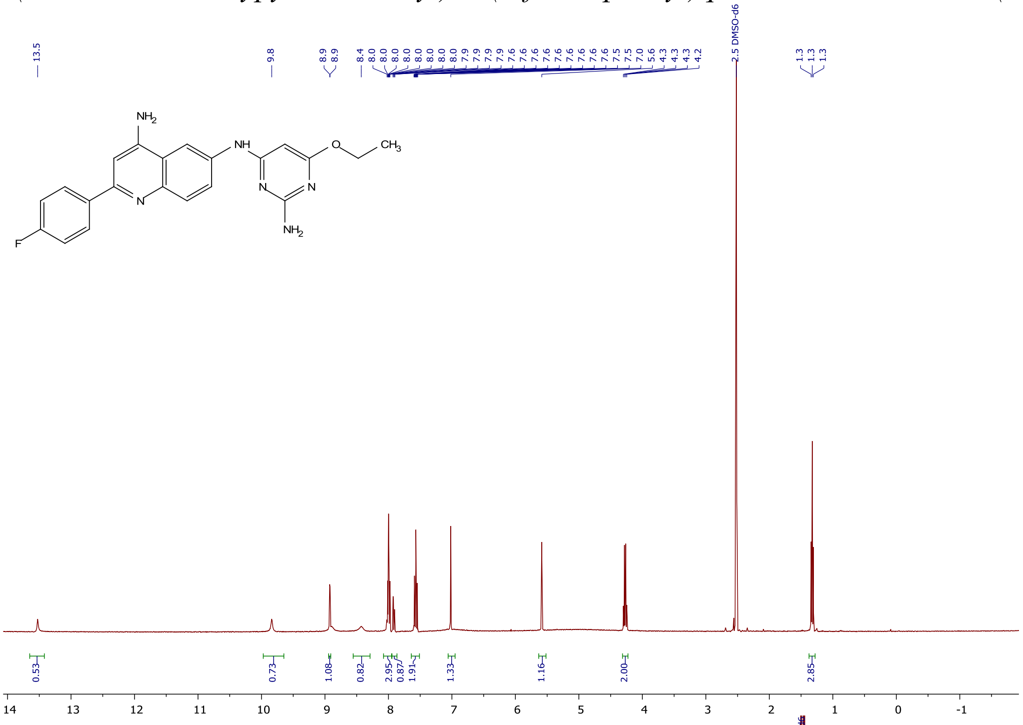

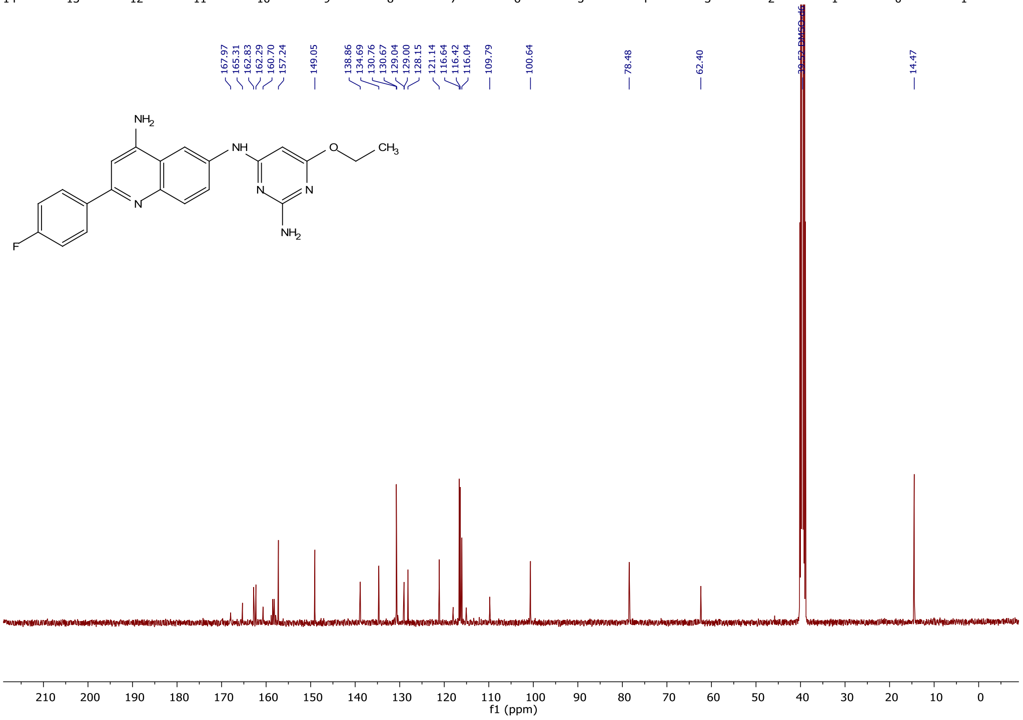

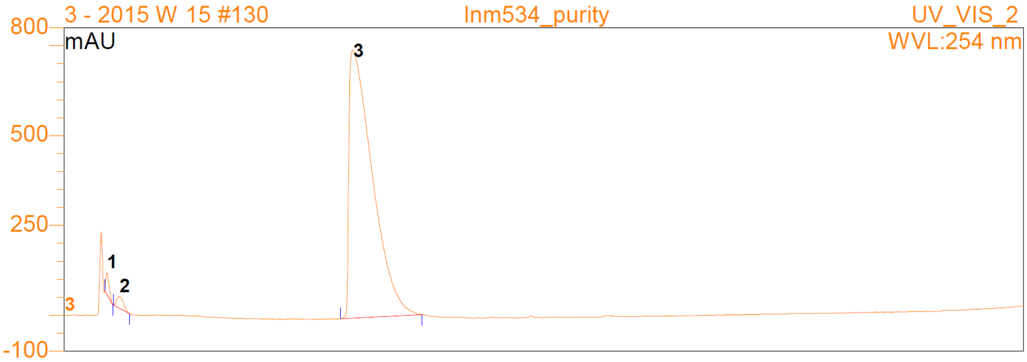

*2-((2-Amino-6-((4-amino-2-(4-fluorophenyl)quinolin-6-yl)amino)pyrimidin-4-yl)oxy)ethan-1-ol (**9b**).*

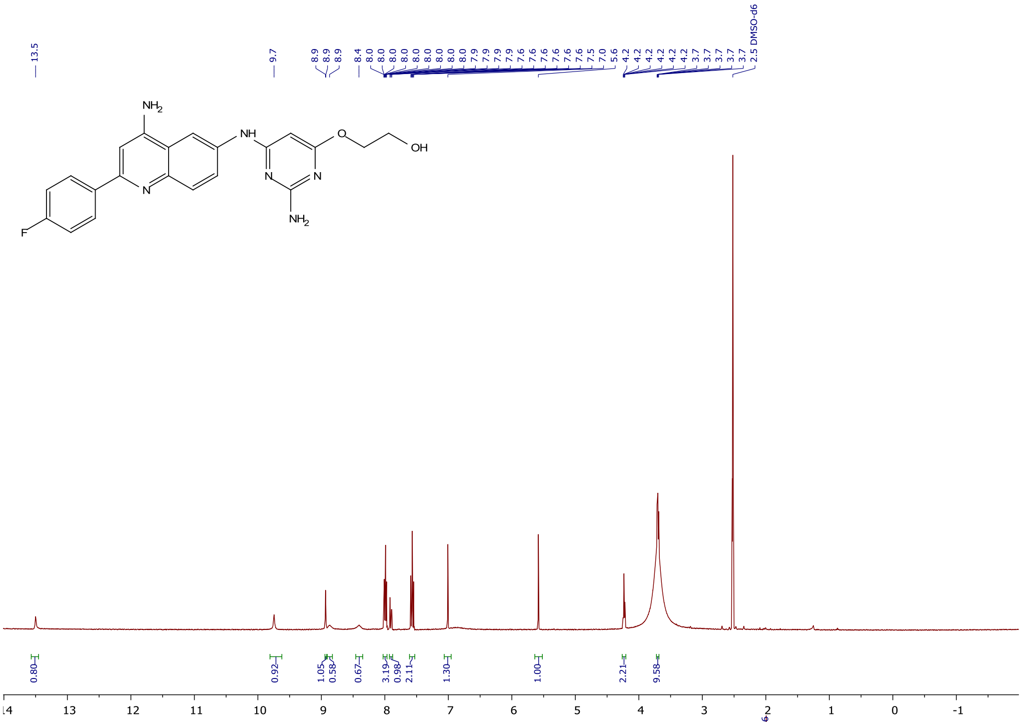

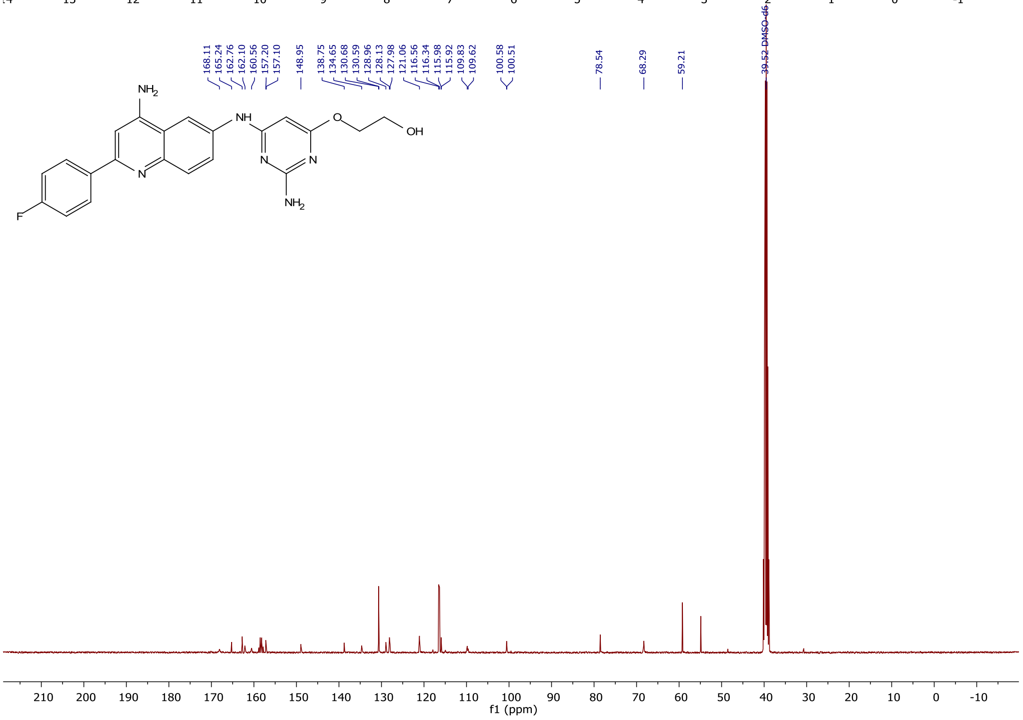

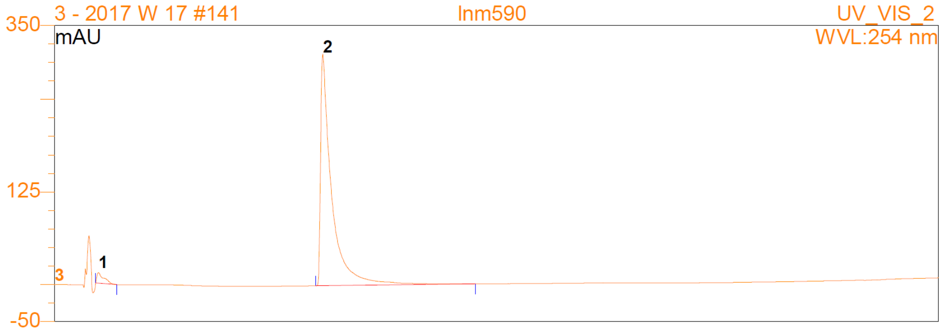

N*6-(2-Amino-6-((1-methylpiperidin-3-yl)methoxy)pyrimidin-4-yl)-2-(4-fluorophenyl)quinoline-4,6-diamine* (**9c**).

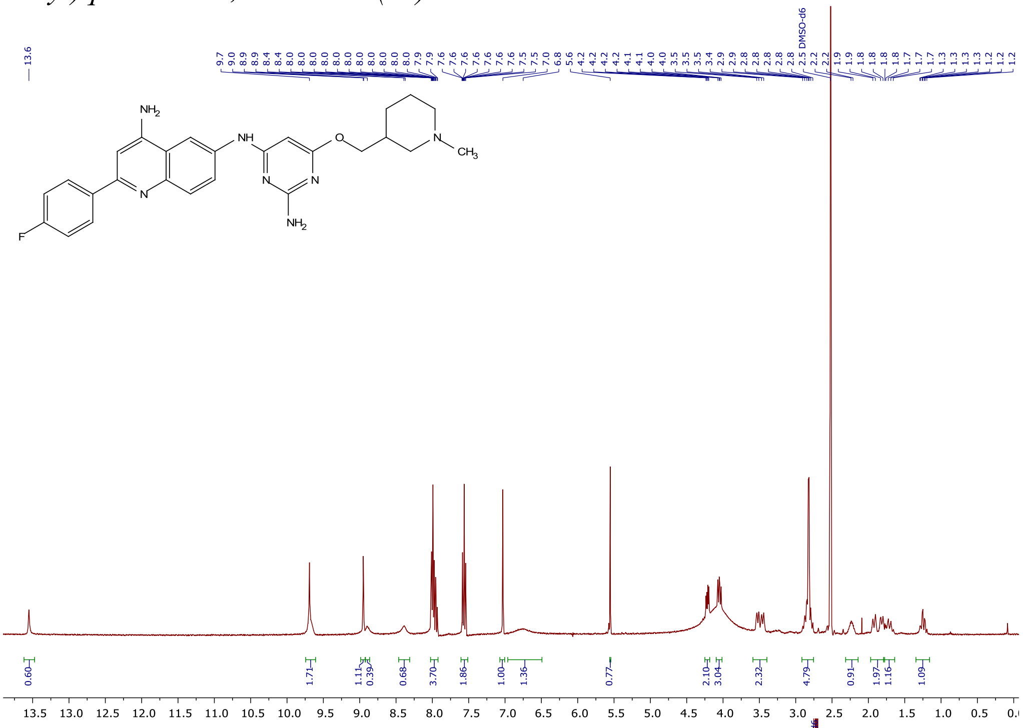

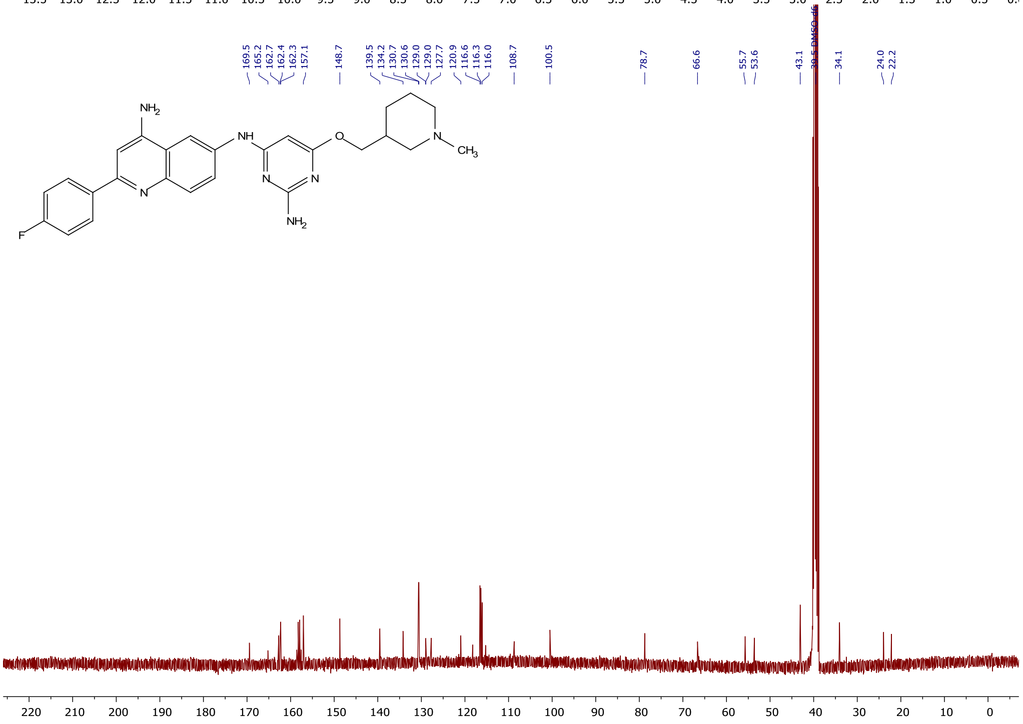

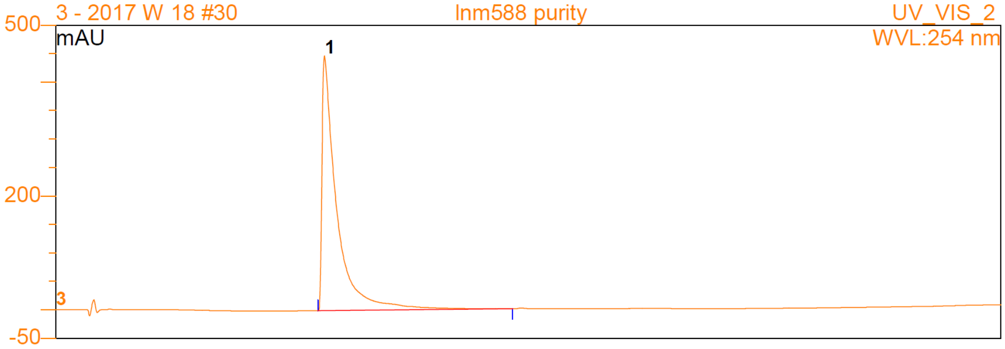

*2-Amino-6-chloropyrimidin-4(3*H*)-one (**11**).*

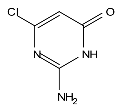

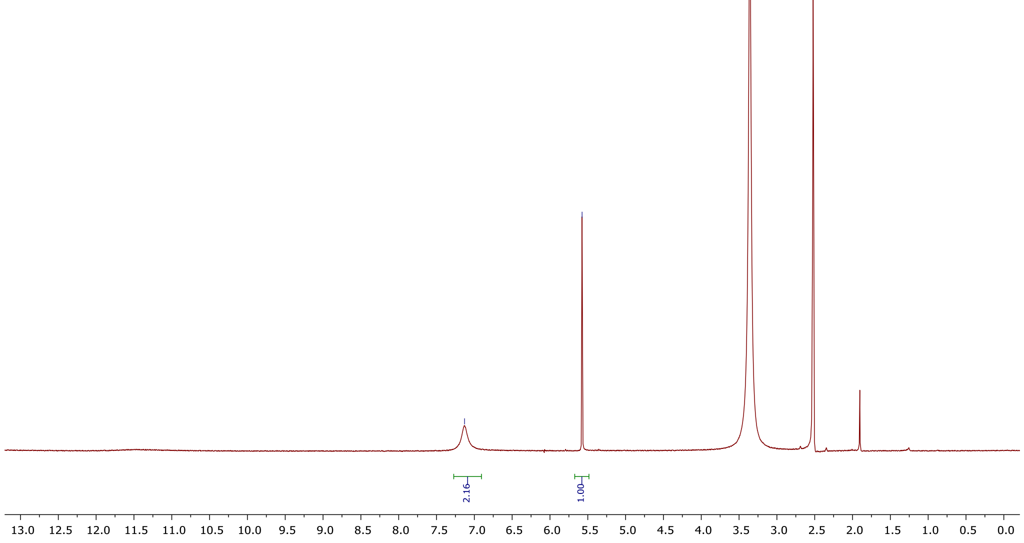

*2-Amino-6-((4-amino-2-(4-fluorophenyl)quinolin-6-yl)amino)pyrimidin-4(3H)-one (**12**).*

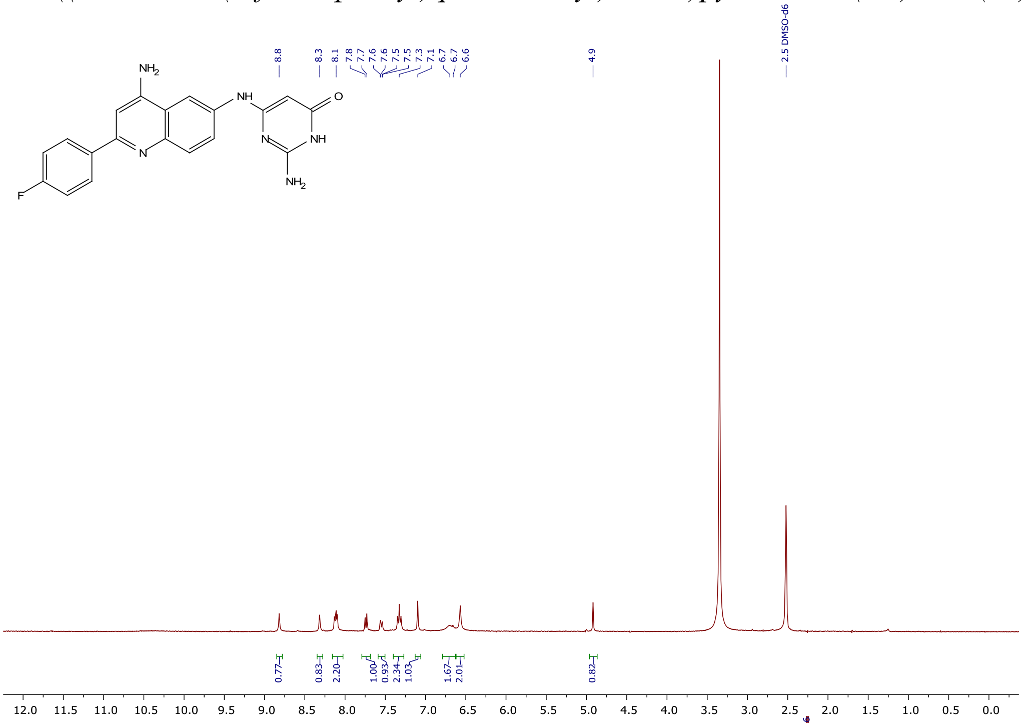

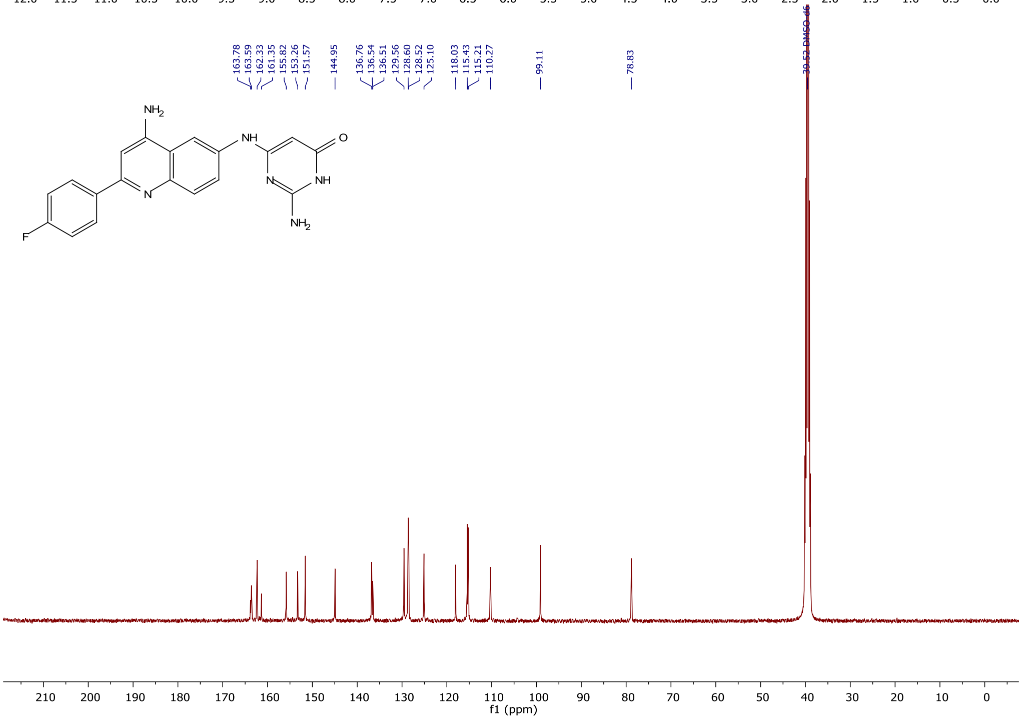

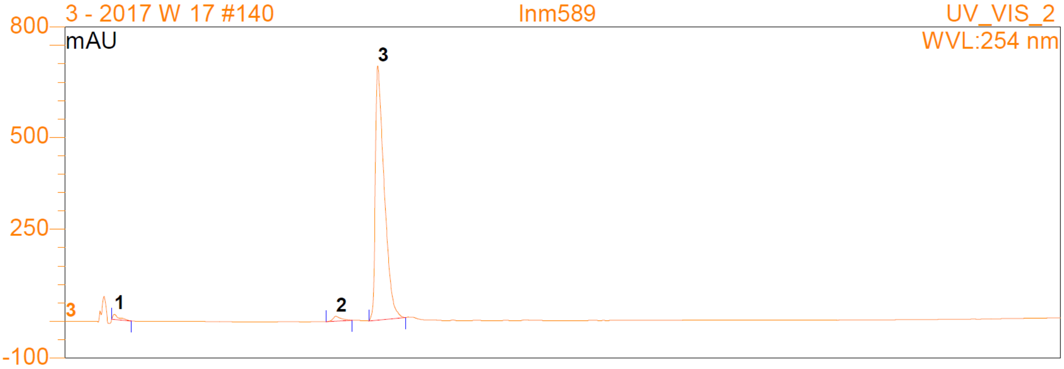

N6-*(6-Chloropyrimidin-4-yl)-2-(4-fluorophenyl)quinoline-4,6-diamine* (**14**).

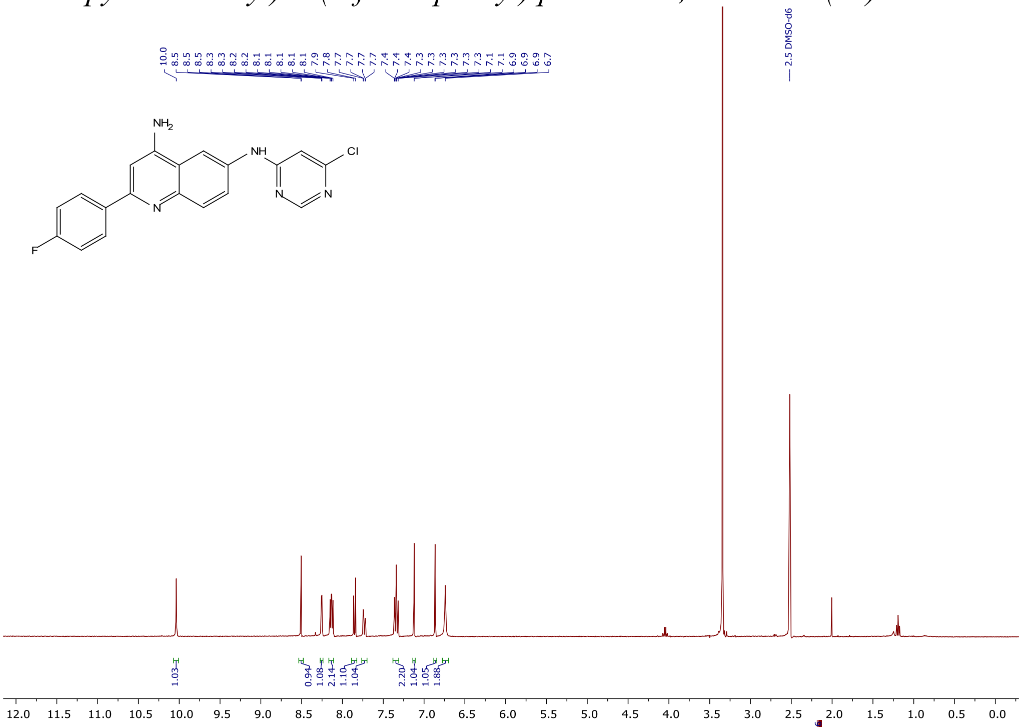

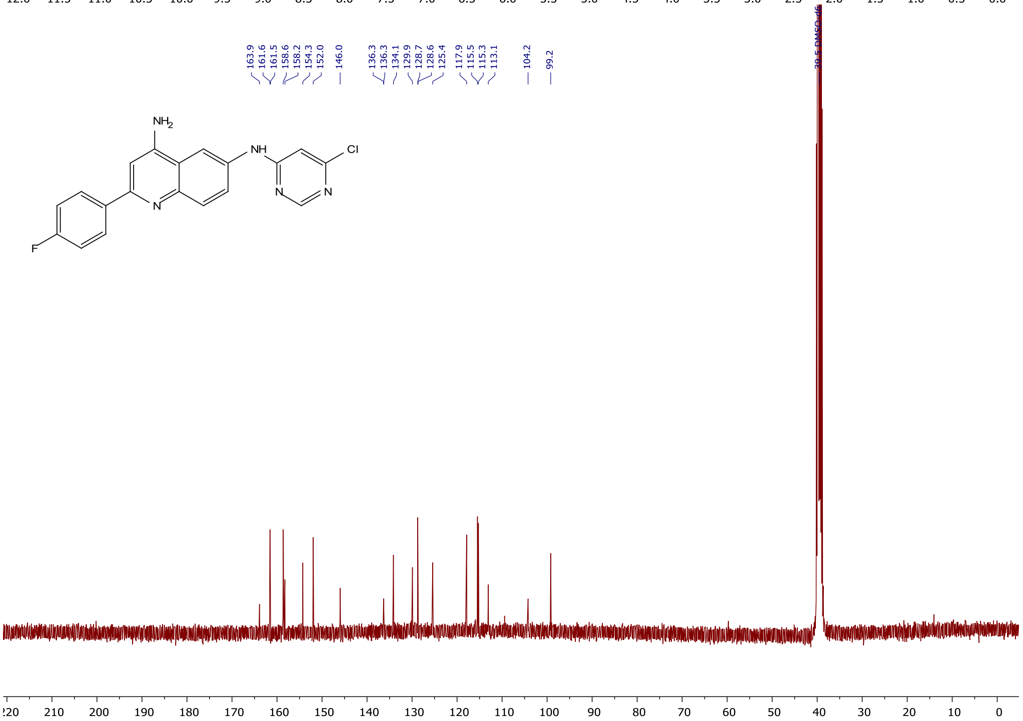

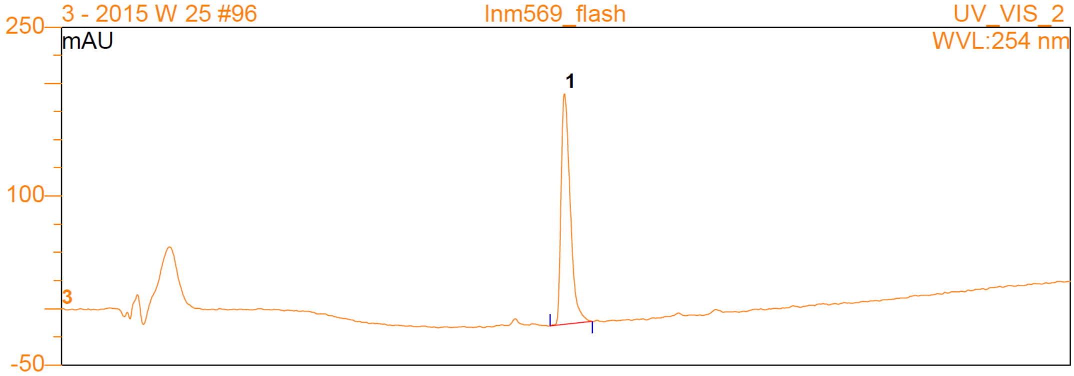

*2-(4-Fluorophenyl)-N6-(6-(piperazin-1-yl)pyrimidin-4-yl)quinoline-4,6-diamine (**15**).*

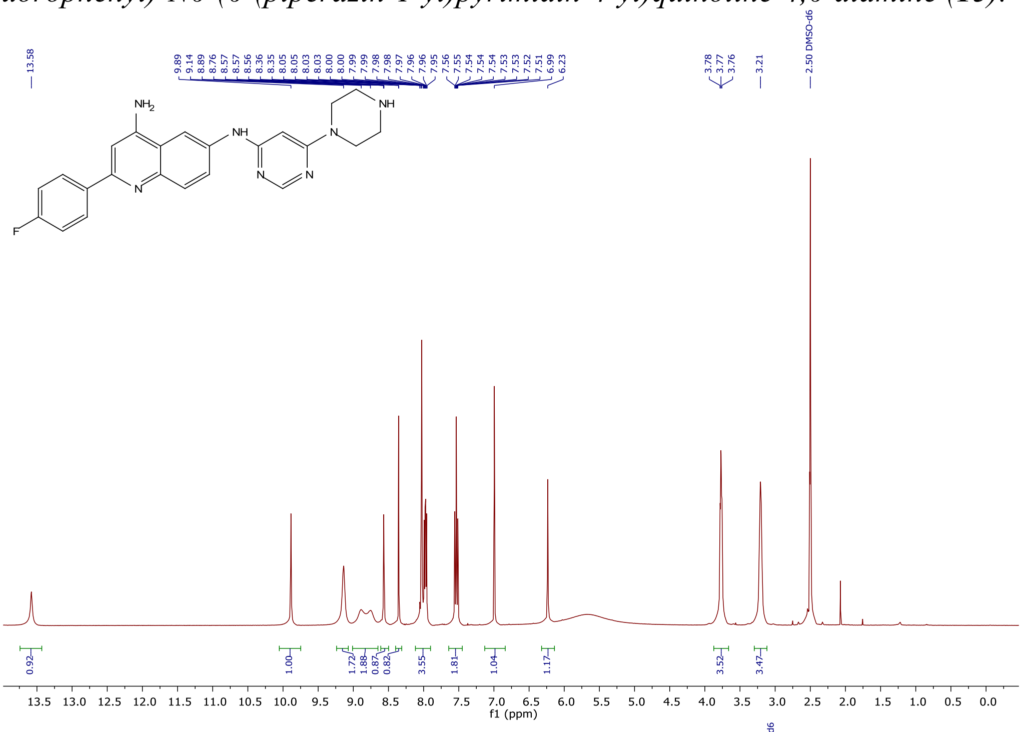

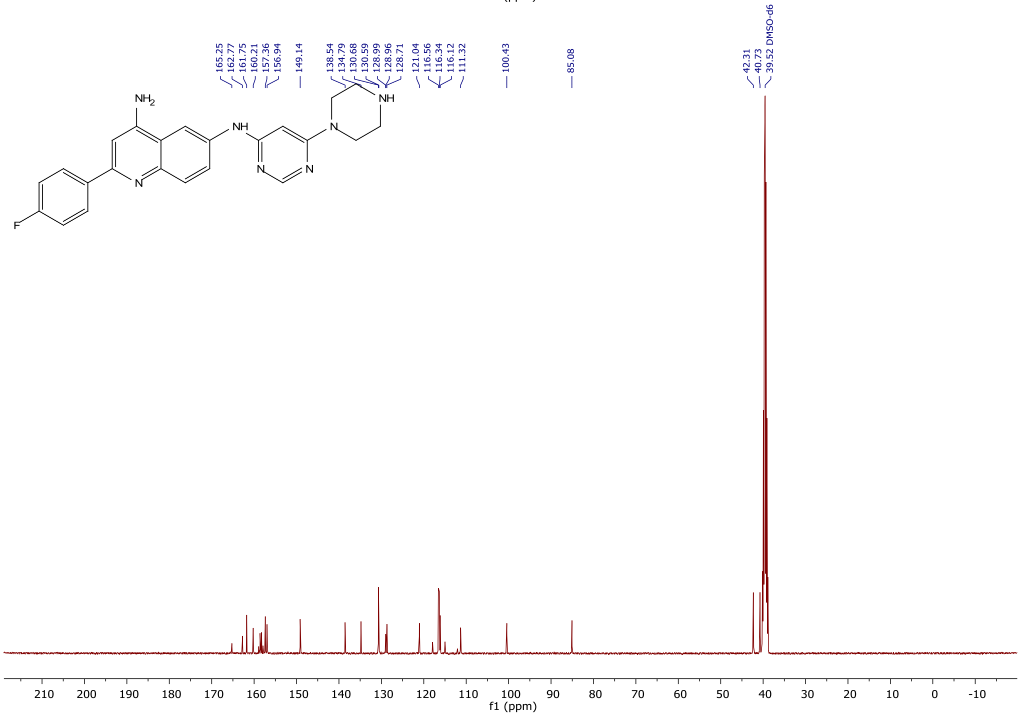

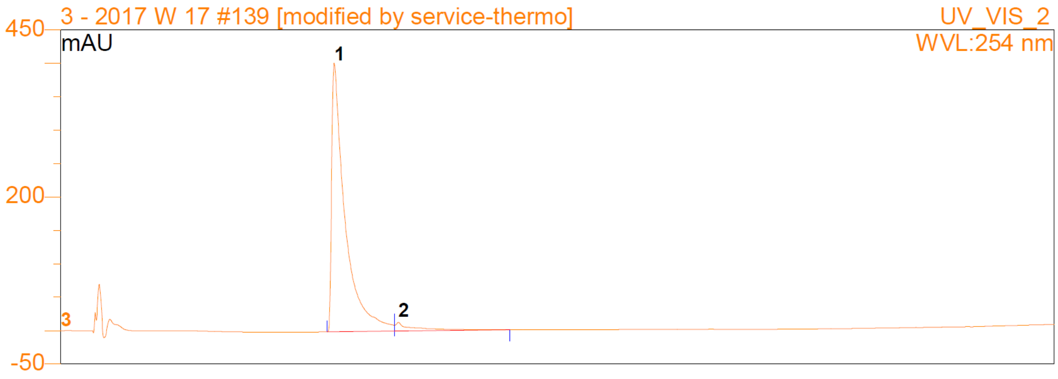

*2-(4-Fluorophenyl)-6-nitroquinoline (**18**).*

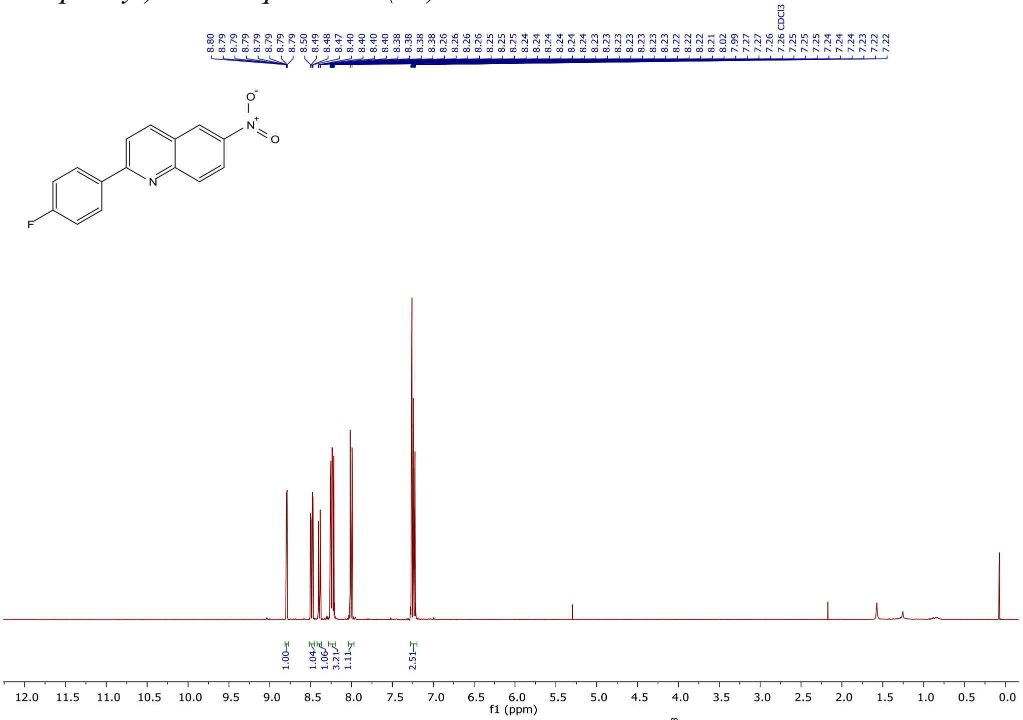

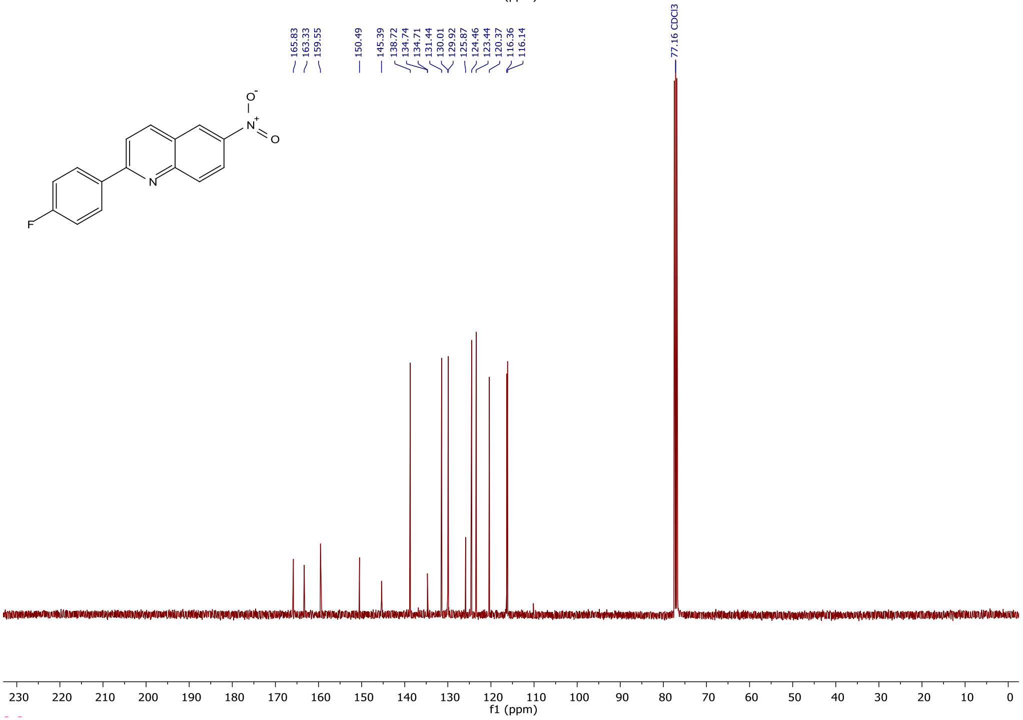

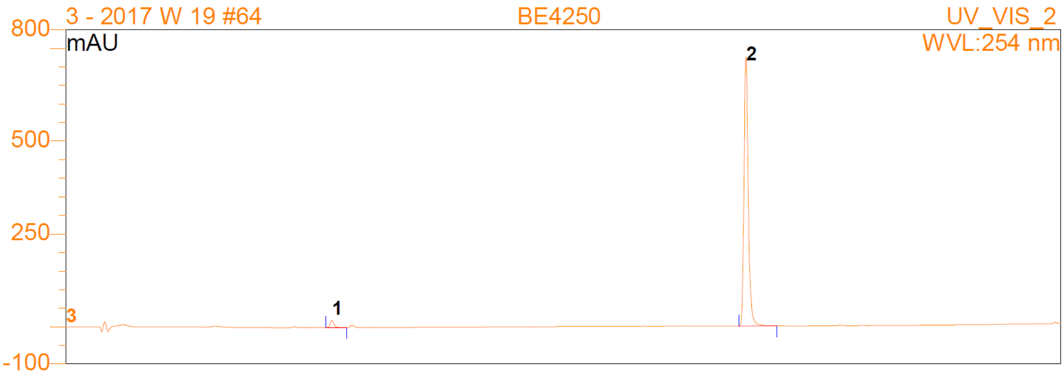

*2-(4-Fluorophenyl)quinolin-6-amine (**19**).*

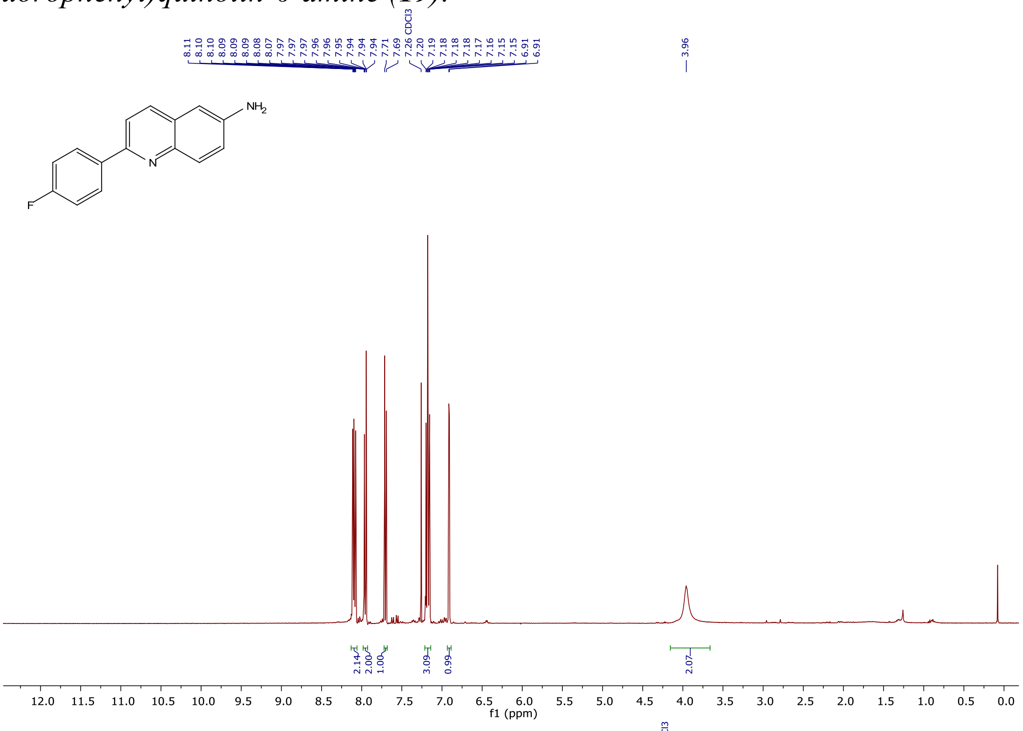

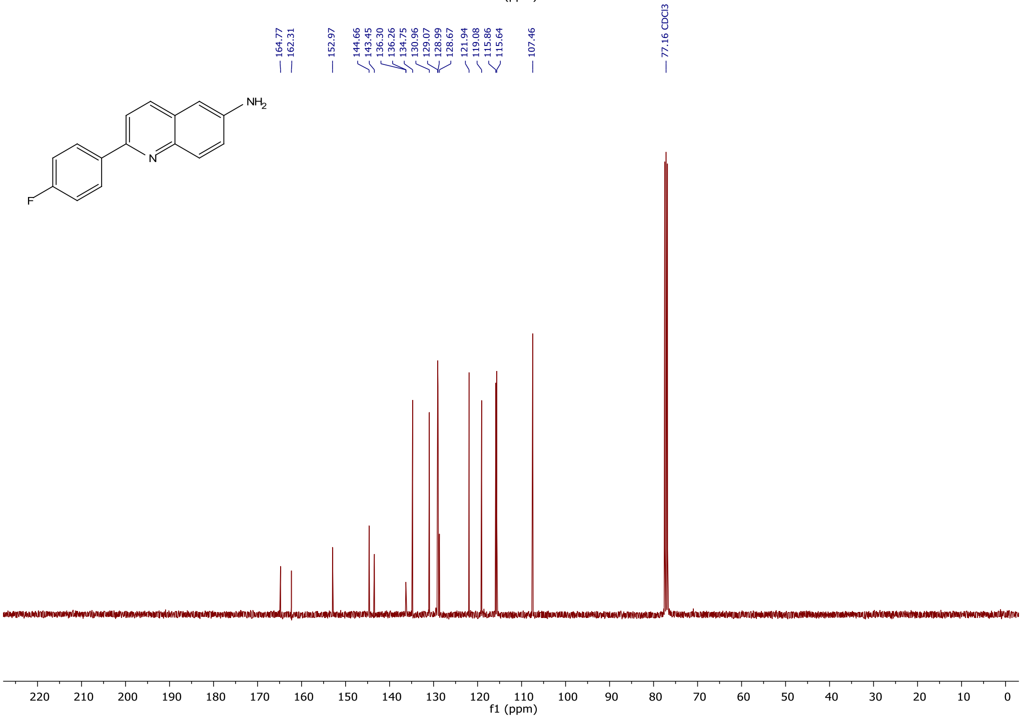

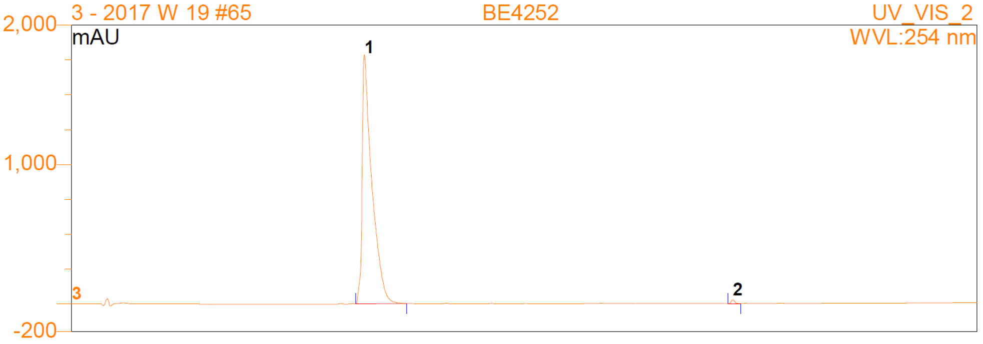

*6-Chloro-*N4*-(2-(4-fluorophenyl)quinolin-6-yl)pyrimidine-2,4-diamine* (**20**).

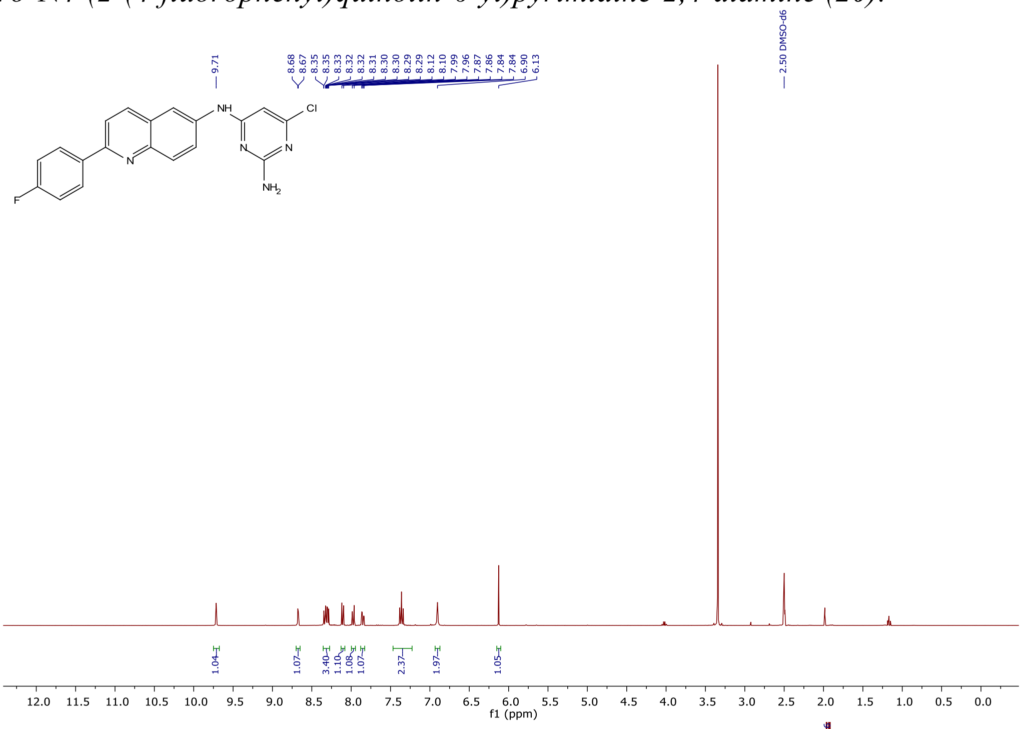

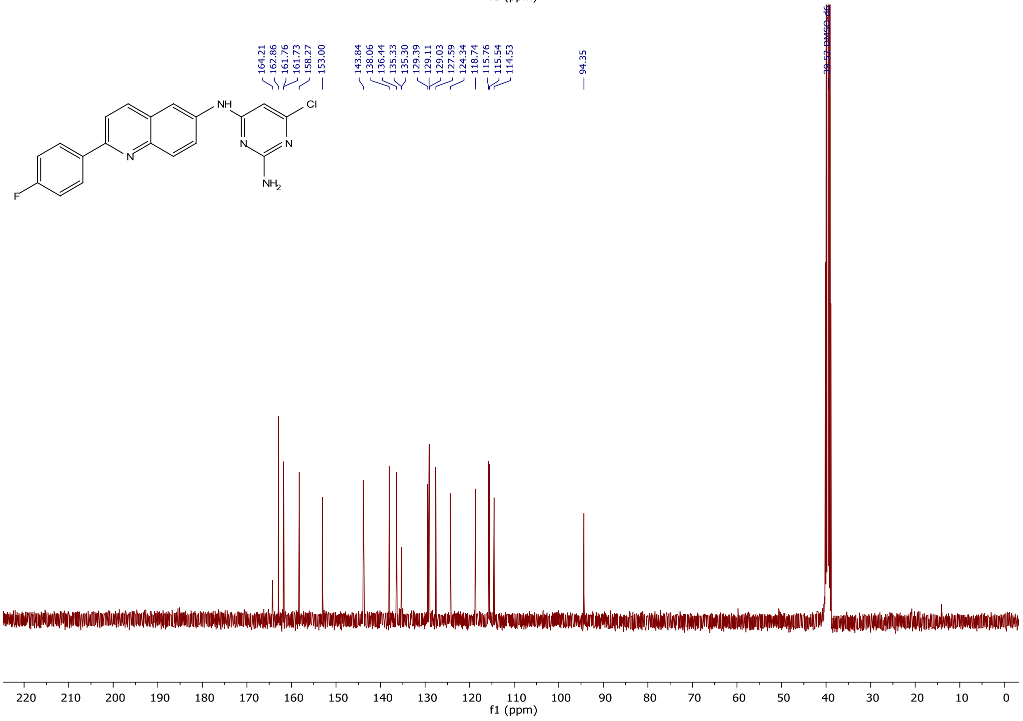

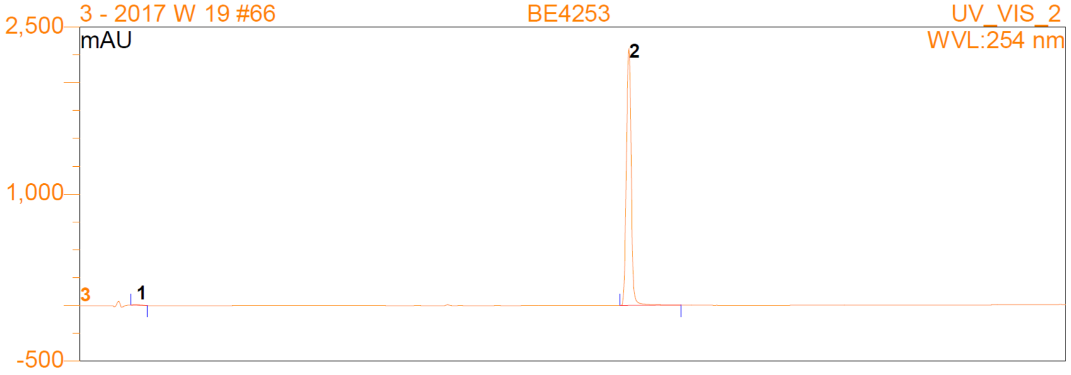

N*4-(2-(4-Fluorophenyl)quinolin-6-yl)-6-(piperazin-1-yl)pyrimidine-2,4-diamine* (**21**).

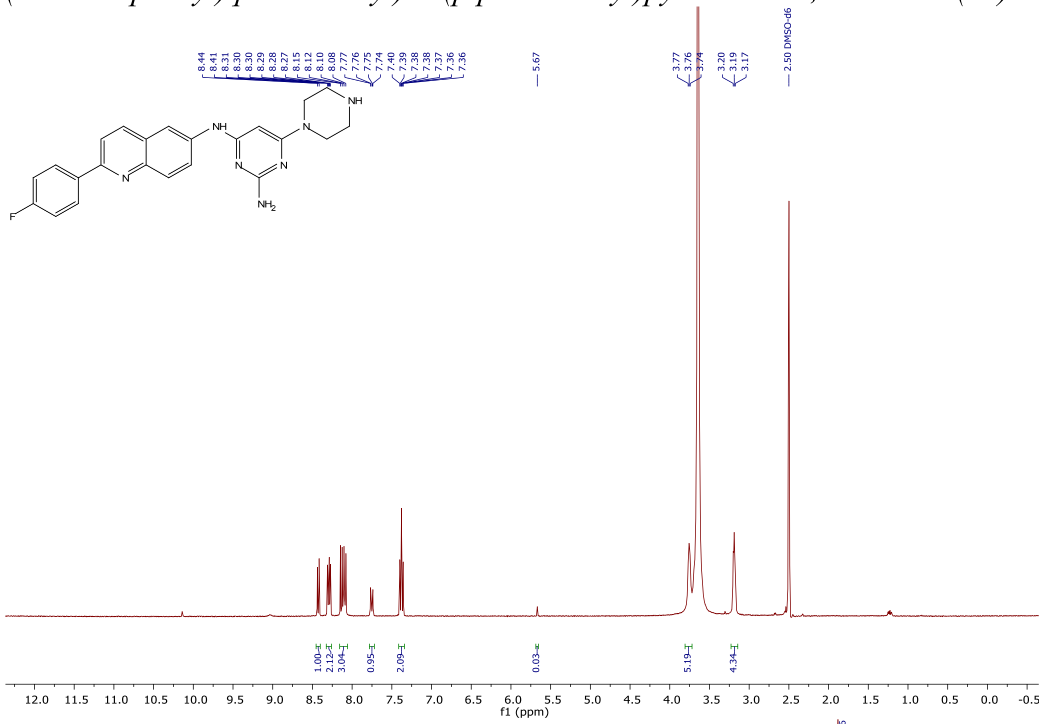

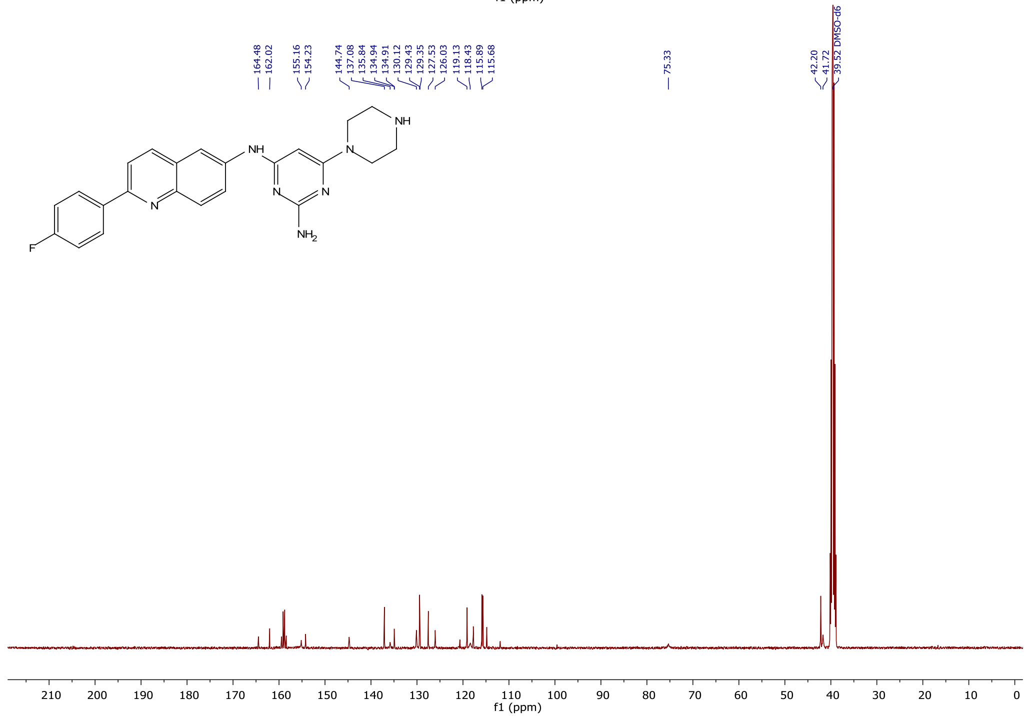

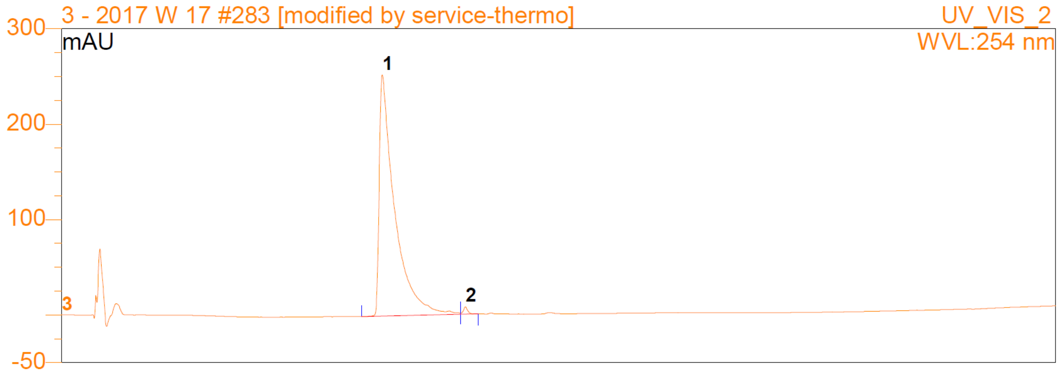

N-(6-Chloropyrimidin-4-yl)-2-(4-fluorophenyl)quinolin-6-amine (**22**).

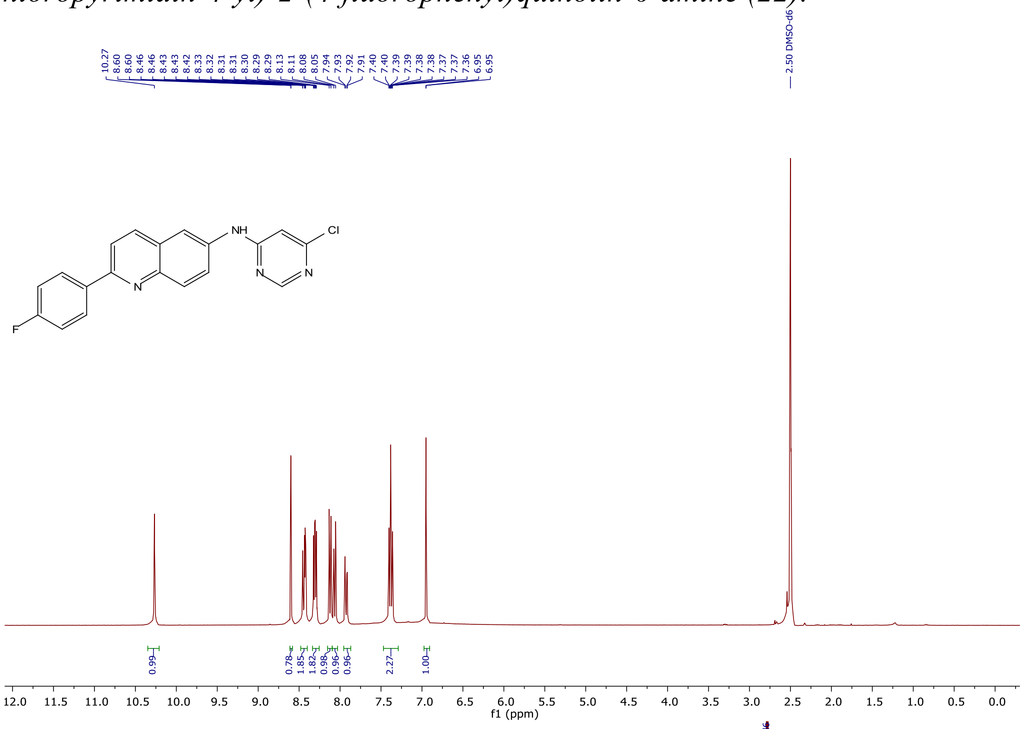

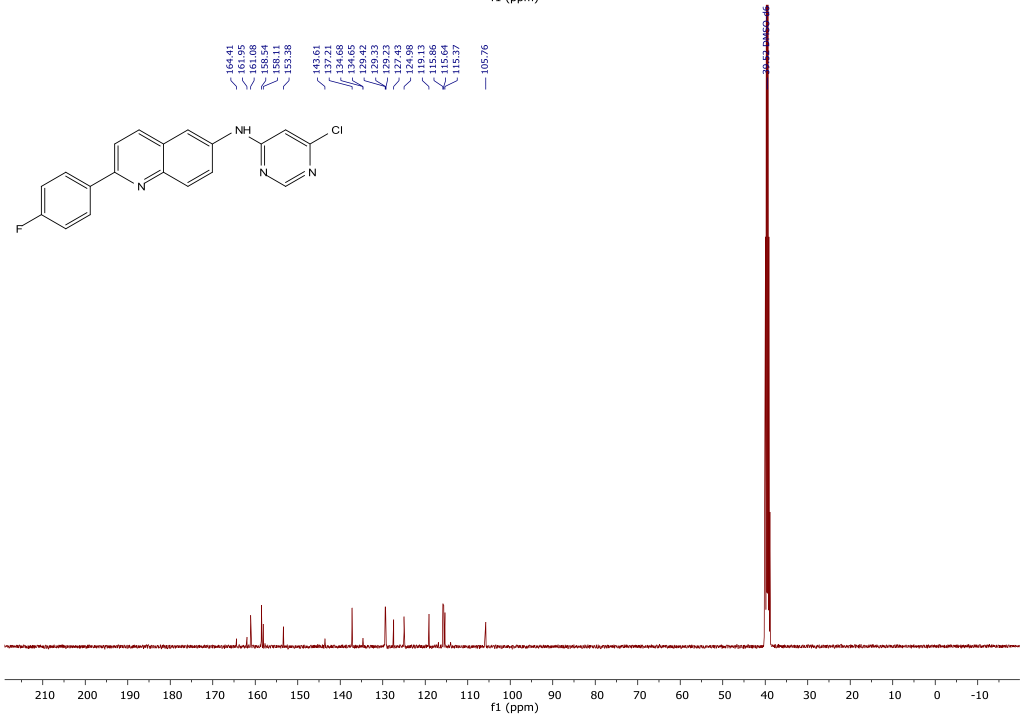

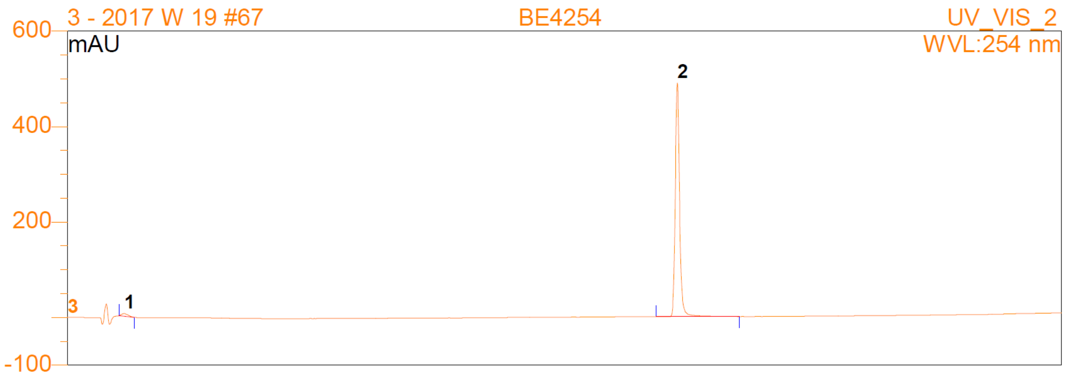

HSQC

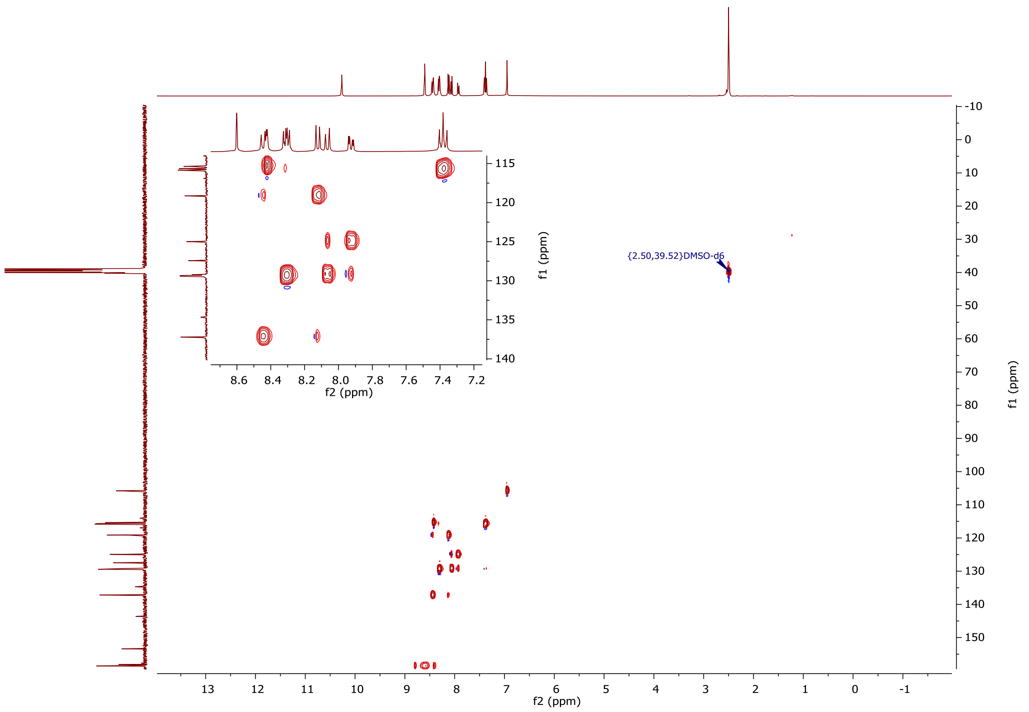

HMBC

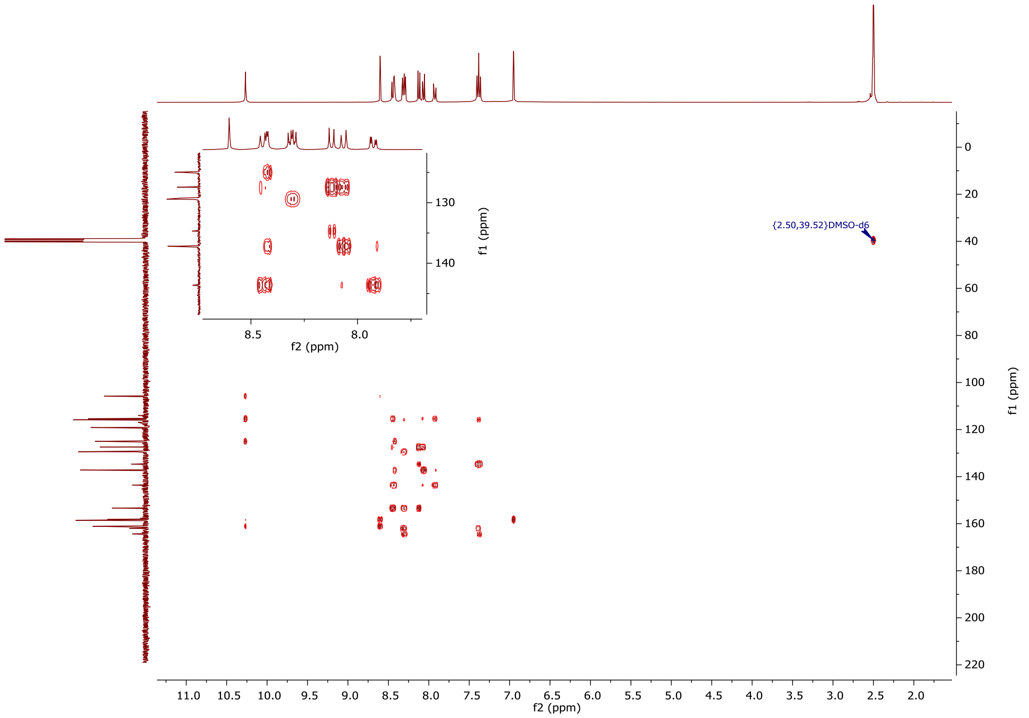

2-(4-Fluorophenyl)-N-*(6-(piperazin-1-yl)pyrimidin-4-yl)quinolin-6-amine* (***23***).

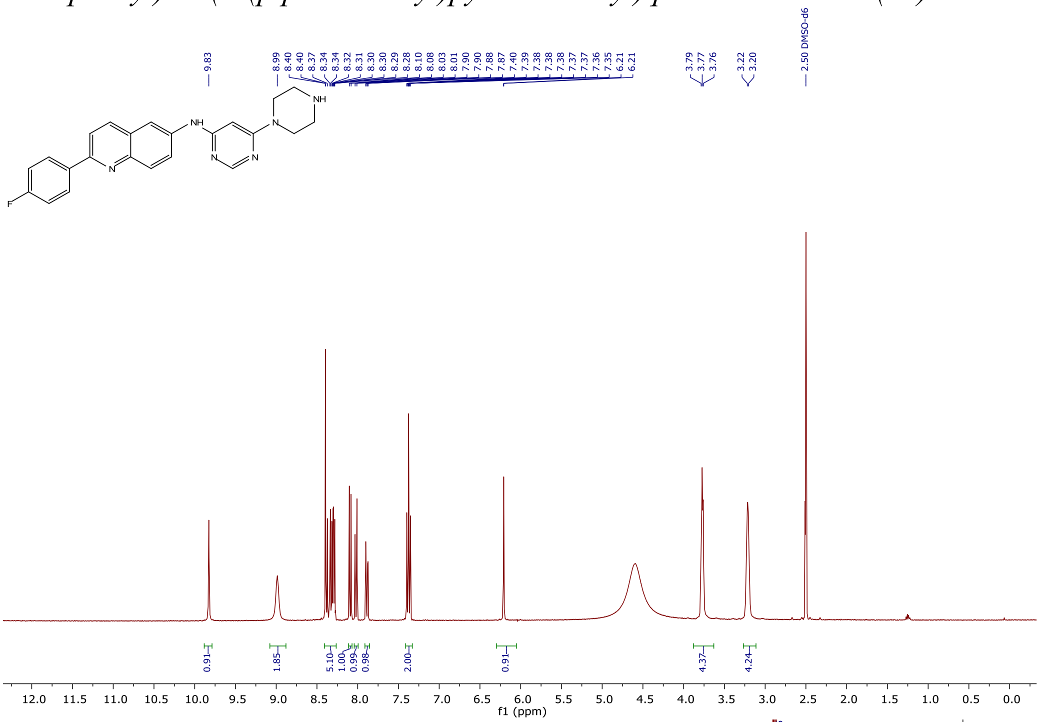

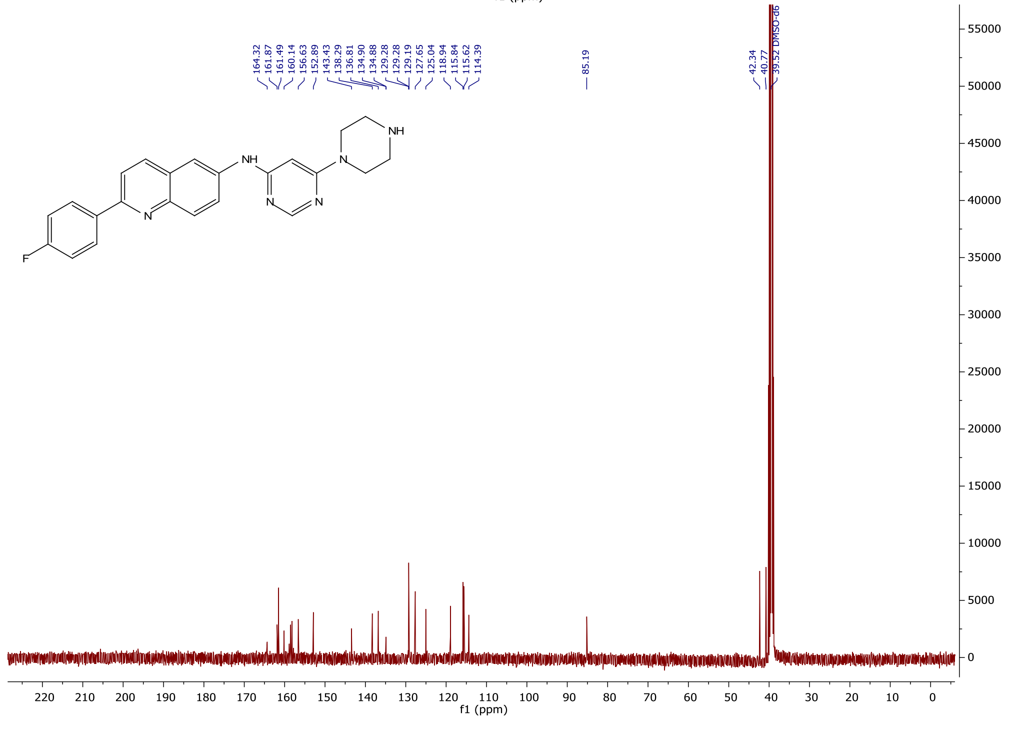

HSQC

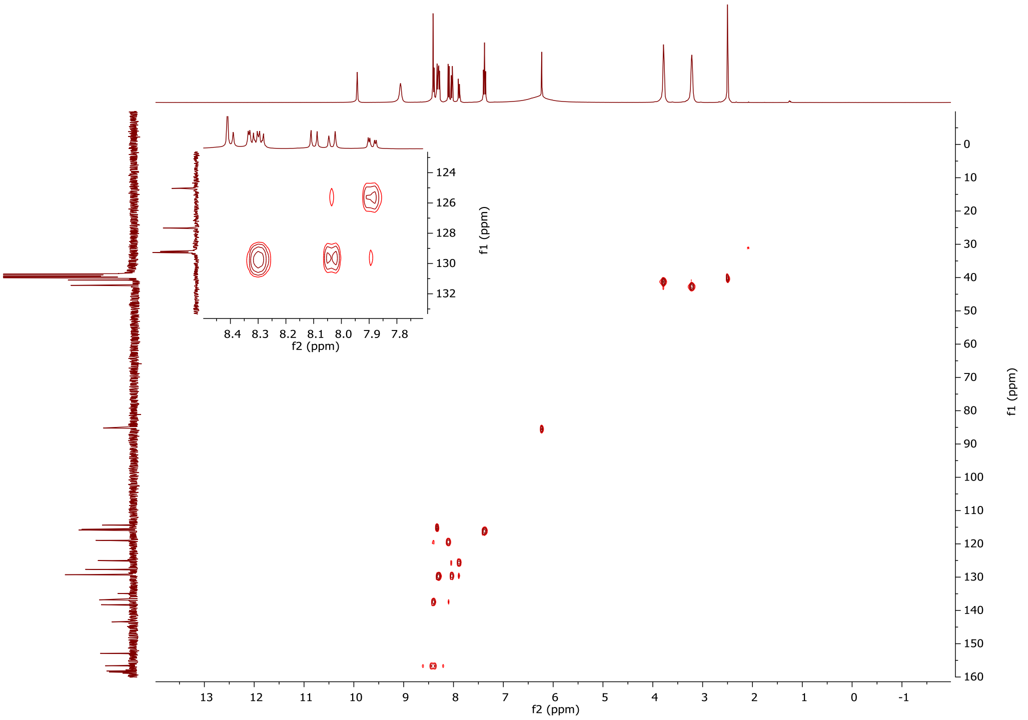

f2 (ppm)
f1 (ppm)

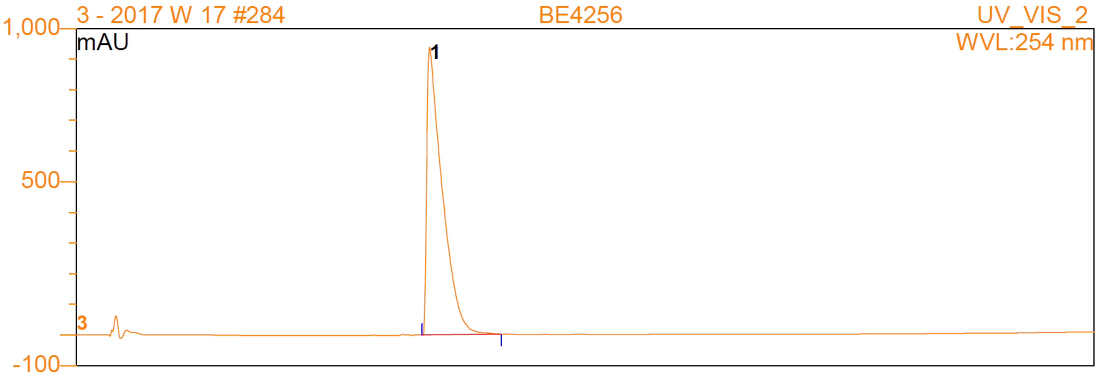

3 - 2017 W 17 #284
BE4256
UV_VIS_2
WVL:254 nm
mAU
1,000
500
-100

2. Biology

2.1. *In vitro* biology: Determination of half maximal inhibitor concentration (IC_{50})

2.1.1. Cloning, expression and purification of the NDH-2 proteins.

A C-terminal His[6]-tagged version of the full-length *M. tuberculosis* NDH-2 gene (*Mt*NDH-2, Rv1854c, Gene ID: 886430 from strain H37Rv) was amplified from pAZI9018-NDH-2 vector using a pair of primers, Forward- TTTTTCCATGGGCAGTCCCCAGCAAGAAC and Reverse-TTTGGATCCCTAATGATGATGATGATGATGGC, and subcloned into a mycobacteria shuttle vector pYUB28b between NcoI and BamHI restriction sites to prepare pYUB28b-*Mt*NDH-2 expression construct. First, the pYUB28b-*Mt*NDH-2 construct was transformed into *E. coli* Top10 cells and plated on LB-agar solid medium supplemented with 50 μg/ml hygromycin B to isolate the positive clones. The protein-coding region of the construct was verified by DNA sequencing. Finally, the pYUB28b-*Mt*NDH-2 construct was transformed into the expression host *M. smegmatis* strain $mc^2$4517 (kindly provided by Professor William R. Jacobs, Albert Einstein College of Medicine, USA) by electroporation. The electroporation was carried out using a Bio-Rad Gene Pulser instrument with the following parameter settings: R= 1000Ω, V = 2.5 kV, Q = 25 μF.[3] The positive clones were selected by plating on Middlebrook 7H9/agar/ ADC/Tween solid medium supplemented with 50 μg/ml of hygromycin B and kanamycin.

The expression of *Mt*NDH-2 was performed in autoinduction media (ZYM-5052, 2mM $MgSO_4$ and 0.2x trace elements)[4] supplemented with 0.05% Tween 80 and 50 μg/ml of hygromycin B and kanamycin for 3 days at 37 °C. The cells were harvested by centrifugation (Avanti J-26S XP, JLA9.100 rotor, Beckman Coulter) at 6000g for 15 min and washed with 0.1 M potassium phosphate, pH 6.8 buffer in the presence of 1 mM PMSF. The washed cells were resuspended in a lysis buffer (0.1 M potassium phosphate, pH 6.8, 1 mM PMSF) containing EDTA-free complete protease inhibitor cocktail (Roche) and sonicated on ice followed by detergent-solubilization for 2 hours at 4 °C in the presence of 2% (w/v) Big CHAP. The detergent-solubilized fraction containing His-tagged *Mt*NDH-2 was separated from the insoluble content by centrifugation (20000g for 30 min at 4 °C) and incubated with pre-equilibrated (0.1 M potassium phosphate, pH 6.8, 0.25% Big CHAP and 10 mM imidazole) TALON resin (Clontech) for 1 hour at 4 °C. After several washing steps with imidazole gradient, the *Mt*NDH-2 was eluted with elution buffer (0.1 M potassium phosphate, pH 6.8, 0.25% Big CHAP) containing 150 mM imidazole and buffer exchanged to 50 mM HEPES, pH 7.0, 300 mM KCl, 20% glycerol, 2 mM $MgCl_2$ and 0.2% (w/v) Big CHAP using a PD-10 column (GE Healthcare Biosciences). The protein concentration of *Mt*NDH-2 was determined by measuring the concentration of FAD co-factor using spectrophotometric flavin (FAD) analysis.[5]

A full-length NDH-2 gene from *M. smegmatis* (*Ms*NDH-2, MSMEG_3621, Gene ID: 4531957 from str. MC2 155) was amplified with His-tags from the genomic DNA and cloned into the pEXP5-NT vector (Invitrogen) using TOPO ligation.

Primers for introducing a C-terminal His_6-tag into *Ms*NDH-2 were:
forward: ATGAGCCATCCCGGAGCTACG;
reverse: CTAatgatgatgatgatgatgGGACGCGGCTTTCTCGGT.

For *Ms*NDH-2, a high temperature (64 °C) was required for denaturation, due to the high CG content. PCR product (17 μL) was mixed with 2 μL Taq buffer, 0.5 μL dNTPs and 0.2 μL Taq enzyme, and incubated at 72 °C for 10 min. A 1 μL sample of this mixture was added to 0.5 μL salt solution (from the pEXP5-NT/TOPO TA Expression Kit, Invitrogen), 1 μL water and 0.5 μL pEXP5-CT vector, and incubated at 22 °C for 20 min. Correct insertion of the desired sequence was confirmed by DNA sequencing. Plasmids were transformed into *E. coli* strain C43(DE3)[1,2] for expression. Experiments with other *E. coli* strains (BL21-AI, BL21(DE3), etc.) did not give satisfactory results.

The transformed cells were cultured in Luria-Bertani medium with 50 mg/L ampicillin at 37 °C. The expression of *Ms*NDH-2 was induced with 0.1 mM IPTG when the cultures reached an OD_{600} of 0.6-0.8. After growth at 22 °C for 21 h, cells were harvested by centrifugation (Avanti J-26S XP, JLA9.100 rotor, Beckman Coulter) at 9,300 g for 10 min, re-suspended and washed with SSP buffer (150 mM NaCl, 10 mM NaH_2PO_4, pH 7.4). The cells were pelleted by centrifugation at 3,000 g for 25 min (Multifuge 3 S-R, Heraeus). The biomass with *Ms*NDH-2 was lysed in lysis buffer (0.1 M potassium phosphate pH 7.0, 1 mM PMSF, plus a complete protease inhibitor cocktail pill (Roche)), by disruption with a continuous disruptor (TS series cabinet, Constant Systems Limited) at 20 Kpsi. After the removal of cell debris by centrifugation at 11,269 g for 10 min, the supernatant was ultracentrifuged at 139,000 g for 2 h. The pelleted membrane fraction was resuspended in 0.1 M phosphate buffer, pH 7.0, containing 15 mM CHAPS, at a protein concentration of 4 mg/ml (determined by Bradford assay). The mixture was incubated at 4 °C for 3 h with gentle stirring. The detergents dodecyl maltoside (DDM), β-octylglucoside and Brij-35 were also tested, but CHAPS gave the best result for *Ms*NDH-2. The supernatant after ultracentrifugation at 139,000 g for 1 h was mixed with 0.5 ml TALON resin (Clontech) pre-equilibrated with 0.1 M potassium phosphate buffer, pH 7.0, containing 8 mM CHAPS (buffer A), and incubated at 4 °C for 3 h with slow rotation.

The resin was then washed with buffer A containing 20 mM imidazole, pH 7.0. *Ms*NDH-2 was eluted from the column with buffer A containing 250 mM imidazole. Concentration of protein was carried out with a Vivaspin Turbo with a cutoff of 30 kDa. Final yield for *Ms*NDH-2 was 3.8 mg per L culture. The purified protein could be concentrated up to 5 mg/mL.

The *Ms*NDH-2 and *Mt*NDH-2 protein sequences are shown below with different amino acids from the wild-type indicated in lower case.

>MSMEG_3621
MSHPGATASDRHKVVIIGSGFGGLTAAKTLKRADVDVKLIARTTHHLFQPLLYQVATGIISEG
EIAPATRVILRKQKNAQVLLGDVTHIDLENKTVDSVLLGHTYSTPYDSLIIAAGAGQSYFGND
HFAEFAPGMKSIDDALELRGRILGAFEQAERSSDPVRRAKLLTFTVVGAGPTGVEMAGQIAE
LADQTLRGSFRHIDPTEARVILLDAAPAVLPPMGEKLGKKARARLEKMGVEVQLGAMVTDV
DRNGITVKDSDGTIRRIESACKVWSAGVSASPLGKDLAEQSGVELDRAGRVKVQPDLTLPGH
PNVFVVGDMAAVEGVPGVAQGAIQGGRYAAKIIKREVSGTSPKIRTPFEYFDKGSMATVSRF
SAVAKVGPVEFAGFFAWLCWLVLHLVYLVGFKTKIVTLLSWGVTFLSTKRGQLTITEQQAY
ARTRIEELEEIAAAVQDTEKAAShhhhhh

>M. tuberculosis H37Rv|Rv1854c| - PROBABLE NADH DEHYDROGENASE NDH
MgSPQQEPTAQPPRRHRVVIIGSGFGGLNAAKKLKRADVDIKLIARTTHHLFQPLLYQVATGII
SEGEIAPPTRVVLRKQRNVQVLLGNVTHIDLAGQCVVSELLGHTYQTPYDSLIVAAGAGQSY
FGNDHFAEFAPGMKSIDDALELRGRILSAFEQAERSSDPERRAKLLTFTVVGAGPTGVEMAG
QIAELAEHTLKGAFRHIDSTKARVILLDAAPAVLPPMGAKLGQRAAARLQKLGVEIQLGAMV
TDVDRNGITVKDSDGTVRRIESACKVWSAGVSASRLGRDLAEQSRVELDRAGRVQVLPDLSI
PGYPNVFVVGDMAAVEGVPGVAQGAIQGAKYVASTIKAELAGANPAEREPFQYFDKGSMA
TVSRFSAVAKIGPVEFSGFIAWLIWLVLHLAYLIGFKTKITTLLSWTVTFLSTRRGQLTITDQQ
AFARTRLEQLAELAAEAQGSAASAKVAShhhhhh

2.1.2. Activity assay.

The activity assay was set up in a plate format. The decrease in NADH concentration was monitored spectrophotometrically at 340 nm with a 2104 Multi-label Reader (Envision, PerkinElmer), using an NADH extinction coefficient of 6220 $cm^{-1}M^{-1}$.

The activity assay for *Mt*NDH-2 was performed using 1.25 nM enzyme. The enzyme and inhibitor were pre-mixed in the assay buffer containing 0.1M HEPES, pH 7.0, 10% DMSO, 0.008% Brij and 200 μM NADH. The substrate mix was prepared in 0.1 M HEPES, pH 7.0, 10% DMSO, 0.008% Brij and 50 μM menadione. The inhibitors were pre-incubated with the enzyme at room temperature for 10 min before adding the substrate mix to start the reaction.

The reaction mixture for the *Ms*NDH-2 assay contained 100 mM NaCl, 3 mM CHAPS, 5 μM FAD and 5% (v/v) DMSO in 50 mM HEPES buffer, pH 7.0.

To calculate kinetic parameters for a particular electron acceptor in the activity assay, NDH-2 was incubated with 0.2 or 0.4 mM NADH, then the reaction was started by adding the electron acceptor at a range of concentrations. The decrease in absorbance at 340 nm was measured for 0.5 or 2 h, and rates were determined from linear portions of each reaction curve. Kinetic parameters were calculated by non-linear fitting of the rates at different substrate concentrations to the Michaelis-Menten equation in GraphPad Prism®.

2.1.3. IC_{50} determination

Inhibitors were prepared as 10 mM stocks in 100% DMSO. Compounds were pre-incubated with NDH-2s in the reaction buffer at room temperature for 10 min before adding menadione to start the reaction. Inhibitors were added in a 2-fold dilution series (ranging from 100 or 20 μM down to 0.002 μM in final concentration). IC_{50} values were calculated by applying non-linear curve fitting to sigmoidal four-parameter dose-response regression in GraphPad Prism®.

2.2. In vitro biology: Minimum inhibitory concentration (MIC) assays on parasites

Synthesized compounds were tested against different parasite cell lines (*T. brucei*, *T. cruzi*, and *P. falciparum* K1) for the MIC determination.

1. *Trypanosoma cruzi* cells were maintained on MRC-5 human lung fibroblast cells. The assay was performed with 5% CO_2 at 37 °C in a 96-well microtiter plate. Each well contains 10 μl compound dilution and 190 μl parasite/MRC-5 cell inoculum (10:1 parasites : cells). Parasite burdens were evaluated with addition of substrate CPRG (50 μl/well stock solution with 15.2mg CPRG + 250 μl Nonidet in 100 ml PBS). After 4h incubation, the absorbance change was measured spectrophotometrically at 540 nm at 37 °C. The results were presented as % growth, compared with non-infected cells (0% growth) and untreated-infected controls (100 % growth) after 7-day incubation, and the IC_{50} is calculated. Benznidazole is used as the reference control with IC_{50} of 1.7 μM. The compound is classified as active when the IC_{50} is lower than **5** μM.
2. *Plasmodium falciparum K1* was isolated in Thailand, and has multidrug resistances to chloroquine, pyrimethamine and cycloguanil. Assay was performed in the environment of 3% O_2, 4% CO_2, 93% N_2 in a 96-well microtiter plate. Each well contains 10 μl compound dilutions and 190 μl malaria parasite inoculum, which has 1% parasitaemia, 2% HCT. Plates were frozen and stored at -20°C after incubated for 72 h. after Thawing, 20 μl of each well is mixed with 100 μl Malstat™ reagent, and 20 μl of 1/1 (v/v) mixture

of PES (phenazine ethosulfate, 0.1 mg/ml) and NBT (Nitro Blue Tetrazolium Grade III, 2 mg/ml) in a new plate. The absorbance change was measured at 655nm. The results were shown as % reduction in parasitemia, compared to control wells with no treatment. Chloroquine with IC50 of 0.21 μM is used as the reference compound. A compound with an IC_{50} lower than 2 μM is considered as active, carried on with evaluation in a secondary screening.

3. *Trypanosoma brucei* sample has $7*10^4$ parasites/ml in the suspension. Cultured for 3 days, the growth of the cells was assessed fluorimetrically with the addition of resazurin in a microtiter plate. The fluorescence intensity was measured after 24h at 37 °C with the excitation wavelength at 550nm and the emission wavelength at 590nm. The results were presented as % reduction in parasite growth, compared to reference wells with no treatment, followed by IC_{50} calculation. Suramine was used as the reference compound with the IC_{50} of 0.04 μM. A compound with IC_{50} higher than 5 μM is classified as the inactive, while a compound with IC50 lower than 1 μM is considered as active, and is subsequently evaluated in a secondary assay, with broader concentration range, additional references, and other species.

IC_{50} values are shown in Table S1, followed by semi-quantitative activity scores, and indications for specificity.

Table S1. IC_{50} results for parasites, as compared to results with the human cell line, MRC-5 (methods described in the main paper).

	IC-50 (µM)					Activity scores				
	1	*2*	*3*	*4*		1	2	3	4	
**Compound**	**MRC-5**	**T.cruz**	**Pf-K1**	**T.bruc**		**MRC-5**	**T.cruz**	**Pf-K1**	**T.bruc**	**Conclusion**
**1**	1.28	0.50	1.29	< 0.25		= 5	= 6	= 5	> 6	aspecific
**7**	1.13	0.51	0.56	0.51		= 5	= 6	= 5	= 5	aspecific
**8a**	24.44	6.45	7.65	8.23		= 2	= 3	= 3	= 3	inactive
**8g**	1.41	0.53	0.81	0.51		= 5	= 6	= 5	= 5	aspecific
**8h**	8.00	8.20	4.49	8.11		= 3	= 3	= 3	= 3	aspecific
**8i**	1.26	0.36	0.50	< 0.25		= 5	= 6	= 6	> 6	aspecific
**8j**	22.03	5.78	3.52	32.22		= 2	= 3	= 4	= 1	aspecific
**8k**	1.75	0.53	0.84	< 0.25		= 5	= 6	= 5	> 6	aspecific
**8l**	7.77	8.14	1.92	2.09		= 3	= 3	= 4	= 4	aspecific
**9a**	1.64	0.51	0.51	< 0.25		= 5	= 6	= 6	> 6	aspecific
**9b**	8.00	7.11	2.08	2.31		= 3	= 3	= 4	= 4	aspecific
**15**	1.73	1.96	1.39	2.04		= 5	= 4	= 5	= 4	aspecific
**21**	8.00	2.15	1.63	0.53		= 3	= 4	= 4	= 5	aspecific
**23**	7.89	2.22	6.17	0.47		= 3	= 4	= 3	= 5	aspecific

3. QSAR Modeling

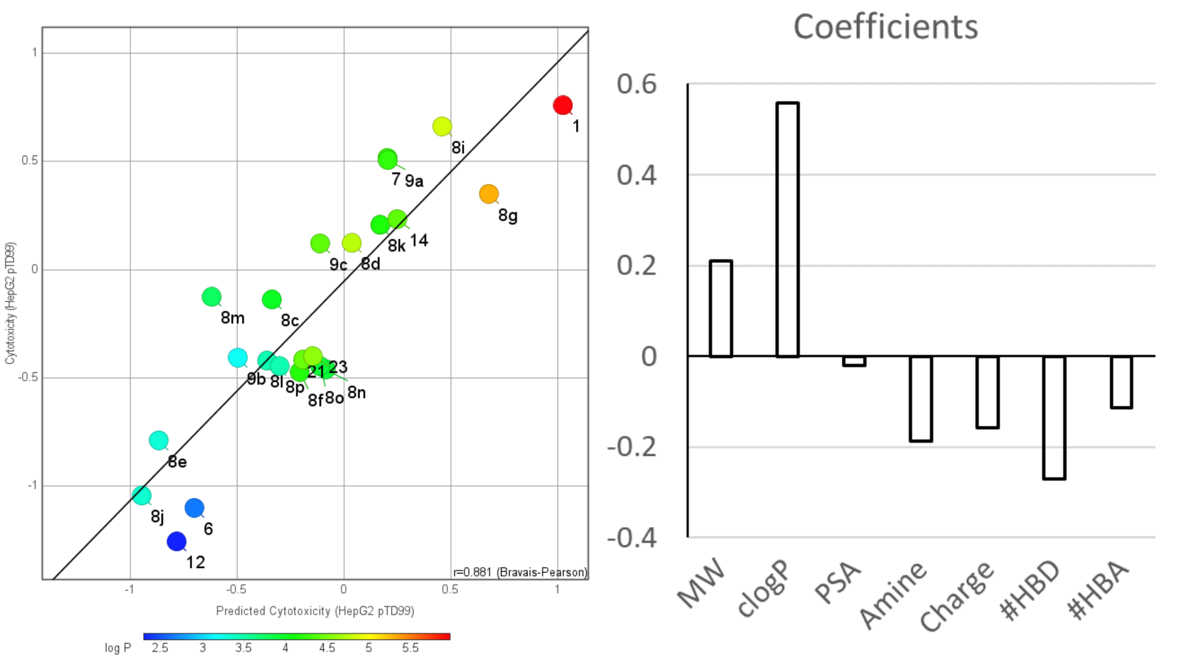

Figure S1. A partial least squares (PLS) model of cytotoxicity in HepG2 cells (pTD99 using mg/mL as unit) based on molecular descriptors (amine refers to the presence or absence of an aliphatic amine). The left panel shows the predicted TD99 *vs*. the experimental values; right panel shows the coefficients.

4. References

1. Miroux B, Walker JE. Over-production of Proteins in Escherichia coli : Mutant Hosts

that Allow Synthesis of some Membrane Proteins and Globular Proteins at High Levels. 1996:289-298.

2. Wagner S, Klepsch MM, Schlegel S, et al. Tuning Escherichia coli for membrane protein overexpression. 2008;105(38):14371-14376.

3. Cirillo, J. D., T. R. Weisbrod, et al. (1993) "Efficient electrotrans- formation of *Mycobacterium smegmatis*." Bio-Rad technical bulletin no. 1360.

4. Studier, F. W. (2005). "Protein production by auto-induction in high density shaking cultures." Protein Expr Purif **41**(1): 207-234.

5. Aliverti, A., B. Curti, et al. (1999). "Identifying and quantitating FAD and FMN in simple and in iron-sulfur-containing flavoproteins." Methods Mol Biol **131**: 9-23.
